# Targeting Discoidin Domain Receptors DDR1 and DDR2 overcomes matrix‐mediated tumor cell adaptation and tolerance to BRAF‐targeted therapy in melanoma

**DOI:** 10.15252/emmm.201911814

**Published:** 2021-12-27

**Authors:** Ilona Berestjuk, Margaux Lecacheur, Alexandrine Carminati, Serena Diazzi, Christopher Rovera, Virginie Prod’homme, Mickael Ohanna, Ana Popovic, Aude Mallavialle, Frédéric Larbret, Sabrina Pisano, Stéphane Audebert, Thierry Passeron, Cédric Gaggioli, Christophe A Girard, Marcel Deckert, Sophie Tartare‐Deckert

**Affiliations:** ^1^ Université Côte d’Azur INSERM, C3M Nice France; ^2^ Equipe labellisée Ligue Contre le Cancer Team MicroCan Nice France; ^3^ Université Côte d’Azur CNRS, INSERM, IRCAN Nice France; ^4^ Aix‐Marseille University CNRS, INSERM, Institut Paoli‐Calmettes, CRCM Marseille France; ^5^ Université Côte d’Azur Centre Hospitalier Universitaire de Nice, Department of Dermatology Nice France

**Keywords:** DDR, extracellular matrix, melanoma, NF‐κB2, therapeutic resistance, Cancer, Skin

## Abstract

Resistance to BRAF/MEK inhibitor therapy in BRAF^V600^‐mutated advanced melanoma remains a major obstacle that limits patient benefit. Microenvironment components including the extracellular matrix (ECM) can support tumor cell adaptation and tolerance to targeted therapy; however, the underlying mechanisms remain poorly understood. Here, we investigated the process of matrix‐mediated drug resistance (MMDR) in response to BRAF^V600^ pathway inhibition in melanoma. We demonstrate that physical and structural cues from fibroblast‐derived ECM abrogate anti‐proliferative responses to BRAF/MEK inhibition. MMDR is mediated by drug‐induced linear clustering of phosphorylated DDR1 and DDR2, two tyrosine kinase collagen receptors. Depletion and pharmacological targeting of DDR1 and DDR2 overcome ECM‐mediated resistance to BRAF‐targeted therapy. In xenografts, targeting DDR with imatinib enhances BRAF inhibitor efficacy, counteracts drug‐induced collagen remodeling, and delays tumor relapse. Mechanistically, DDR‐dependent MMDR fosters a targetable pro‐survival NIK/IKKα/NF‐κB2 pathway. These findings reveal a novel role for a collagen‐rich matrix and DDR in tumor cell adaptation and resistance. They also provide important insights into environment‐mediated drug resistance and a preclinical rationale for targeting DDR signaling in combination with targeted therapy in melanoma.

## Graphical abstract

Resistance to MAPK targeted therapy remains a major challenge in melanoma management. This study shows that BRAF/MEK inhibitor combination induces collagen remodeling that fosters activation of Discoidin Domain Receptors (DDR) and turns on a drug tolerant pathway. Targeting DDR overcomes this pathway.

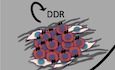

**The paper explained**

**Problem**

Despite novel targeted therapies for BRAF mutant advanced melanoma, therapy‐driven resistance remains a major hurdle that limits patient benefit. Emerging evidence supports that tumor microenvironment components such as the surrounding extracellular matrix play a key role in creating resistant niches by allowing melanoma cells to rapidly adapt and tolerate anti‐cancer drugs. However, how the extracellular matrix contributes to drug resistance remains poorly understood. Thus, a better understanding of the complexities of this tumor–host interaction may offer new therapeutic strategies to obviate melanoma resistance to treatment targeting the BRAF oncogenic pathway.

**Results**

To model the contribution of the extracellular matrix in melanoma cell response to BRAF‐targeted therapy, we generated cell‐derived 3D matrices from fibroblasts isolated from patient‐derived biopsies and analyzed their effectiveness to protect melanoma cells against the anti‐proliferative effect of oncogenic BRAF inhibition. We demonstrate that melanoma cells cultured on matrices adapt to BRAF/MEK inhibition by turning on a drug‐tolerant pathway that is initiated by the tyrosine kinase receptors for collagens DDR1 and DDR2. Clustered DDR activate a non‐canonical NF‐κB2 (p52/RelB)‐resistant pathway that is therapeutically targetable with clinically approved compounds such as imatinib or with a preclinically tested inhibitor of the NIK/NF‐κB2 pathway. In melanoma cell‐derived xenografts, targeting DDR by imatinib enhances BRAF inhibitor efficacy, counteracts therapy‐induced fibrillar collagen remodeling, and delays tumor relapse.

**Impact**

This study outlines the critical interaction between melanoma cells and the extracellular matrix in mediating tolerance to BRAF/MEK inhibitor therapy and reveals that targeting DDR signaling may represent an attractive salvage strategy to overcome collagen‐mediated drug resistance. It provides a rationale for designing clinical trials with clinically approved drugs such as imatinib in BRAF mutant melanoma patients treated with targeted therapies.

## Introduction

One of the hallmarks of cancer cells is their remarkable ability to adapt to microenvironmental influences, such as the nature of the stroma including the extracellular matrix (ECM) and therapeutic stress (Pickup *et al*, 2014). This is particularly true for malignant cutaneous melanoma, which is one of the most aggressive and refractory human cancers (Shain & Bastian, 2016). Approximately 50% of melanoma carries activating mutations in the *BRAF* oncogene, leading to the activation of the mitogen‐activated protein kinase (MAPK)/ERK pathway. Inhibition of the BRAF^V600E/K^ oncoprotein by BRAF inhibitors (BRAFi) such as vemurafenib or dabrafenib has markedly improved clinical outcome of patients (Flaherty *et al*, 2012). Despite this, durable responses are rare as most patients relapse within a year of beginning the treatment. Significant prolonged benefit can be achieved by combining BRAFi and MEK (MAPK/ERK kinase) inhibitors (MEKi) such as cobimetinib or trametinib, yet the development of drug resistance remains the most common clinical outcome (Robert *et al*, 2019). Acquired resistance to targeted therapies involves genetic alterations in key intracellular regulators of the MAPK signaling pathway. This leads to the restoration of the pathway and non‐genetic alterations that are commonly associated with transcriptional reprogramming and phenotype switching from a melanocytic to an invasive undifferentiated mesenchymal‐like cell state, which is characterized by lower expression levels of MITF and SOX10 and higher levels of AXL (Muller *et al*, 2014; Rambow *et al*, 2019). Such adaptive responses to BRAF oncogenic pathway inhibition are thought to precede mutation‐driven acquired resistance (Smith *et al*, 2016).

However, in addition to mechanisms of resistance intrinsic to cancer cells, dynamic, *de novo* mechanisms exist, which are orchestrated by the tumor microenvironment and occur during the cancer cell’s adaptation to therapy. Environment‐mediated drug resistance (EMDR) thus appears as an important contributor to how cancer cells escape therapies (Meads *et al*, 2009). This process has initially been described in multiple myeloma and other hematopoietic malignancies and was shown related to minimal residual disease. This phenomenon is gaining importance in the field of melanoma with several studies reporting the involvement of stroma‐derived factors in adaptive response and resistance to targeted therapies (Straussman *et al*, 2012; Fedorenko *et al*, 2015; Hirata *et al*, 2015; Kaur *et al*, 2016; Young *et al*, 2017). Given the key role of EMDR enabling the emergence of genetic resistance, an understanding and further identification of EMDR mechanisms in melanoma may assist with the development of more effective therapeutic strategies, thereby increasing the efficacy of targeted therapies.

Tumors are complex and adaptive ecosystems that are affected by numerous stromal components, which enhance tumor phenotypes and therapy resistance. Cancer‐associated fibroblasts (CAFs) are activated fibroblasts and the primary producers of ECM. The ECM is a highly dynamic structural framework of macromolecules, providing both biochemical and biomechanical cues, which are required for tumor progression (Kalluri, 2016). The ECM is primarily composed of fibrillar and non‐fibrillar collagens, hyaluronic acid, proteoglycans, and adhesive glycoproteins such as fibronectin, thrombospondins, and SPARC. It also contains matrix‐remodeling enzymes and other ECM‐associated proteins and acts as a reservoir for cytokines and growth factors (Hynes & Naba, 2012; Mouw *et al*, 2014). ECM composition, fiber orientation, and physical characteristics are profoundly altered in the vast majority of solid tumors (Pickup *et al*, 2014). Interactions between cells and the ECM elicit intracellular signaling pathways and regulate gene transcription, mainly through cell‐surface adhesion receptors including integrins and discoidin domain receptors (DDR). DDR1 and DDR2 belong to a unique subfamily of receptor tyrosine kinases and have been identified as non‐integrin collagen receptors (Shrivastava *et al*, 1997; Vogel *et al*, 1997; Leitinger, 2014). They are distinguished from each other by their relative affinity for different types of collagens, as DDR1 is activated by both fibrillar and non‐fibrillar collagens, whereas DDR2 is only activated by fibrillar collagens. Furthermore, their expression and function are associated with fibrotic disease and cancer (Valiathan *et al*, 2012; Leitinger, 2014). DDR1 or DDR2 is known to control tumor cell proliferation and invasion, depending on the tumor type and the nature of the microenvironment (Valiathan *et al*, 2012). However, the functional role of DDR activity in mediating sensitivity to anti‐cancer therapies and tumor resistance is poorly documented.

Adhesion of tumor cells to the ECM is a key component of EMDR. However, the influence of matrix‐mediated drug resistance (MMDR) in response to targeted therapies and the nature of ECM receptors driving the MMDR phenotype in melanoma have not yet been addressed in detail. To model the contribution of the ECM in melanoma cell responses to BRAF and MEK inhibition, we generated fibroblast‐derived 3D ECM from melanoma‐associated fibroblasts (MAFs), which were isolated from patient‐derived biopsies and analyzed the MMDR mechanism with the aim to identify novel opportunities for microenvironment‐targeted therapies. Here, we show that DDR1 and DDR2 are key mediators of MMDR in melanoma, through the pro‐survival non‐canonical NF‐κB2 pathway. Our findings reveal a novel role for these collagen‐activated tyrosine kinase receptors, in mediating BRAF inhibitor tolerance. These data therefore support the rationale to inhibit DDR1 and DDR2 signaling, to disrupt the therapy‐resisting properties conferred by the ECM in the microenvironment. We propose that the use of DDR inhibitors as a novel combinatorial therapeutic strategy may be beneficial for melanoma patients in overcoming resistance to MAPK‐targeted therapy.

## Results

### Fibroblast‐derived 3D ECM confers drug‐protective action to melanoma cells against anti‐BRAF^V600E^ therapies

**Figure 1:**
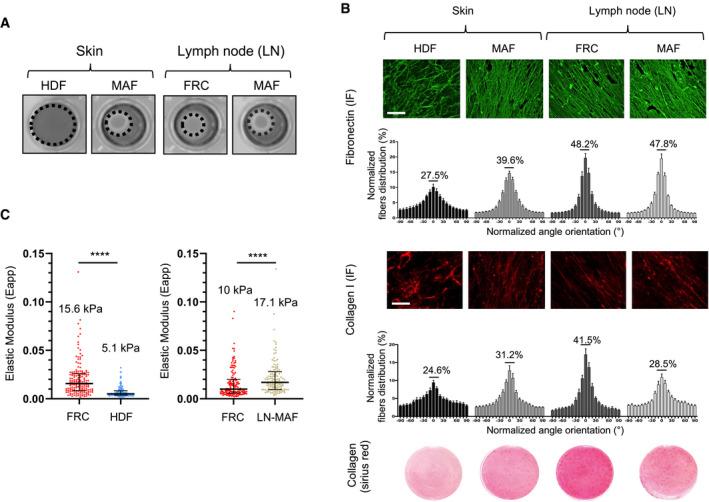
Composition, topology, and mechanical properties of fibroblast‐derived 3D ECMs Images show collagen matrix gel contraction by HDF (human dermal fibroblasts), skin‐MAF (melanoma‐associated fibroblasts isolated from skin lesions), LN‐FRC (lymph node fibroblast reticular cells), and LN MAF (melanoma‐associated fibroblasts isolated from metastatic lymph node). Dashed circles represent the diameter of the gel. Data are representative of *n* = 3 independent experiments.Immunofluorescence analysis of fibronectin (green) and collagen (red) fibers on de‐cellularized ECM produced by human fibroblasts. Fiber orientation was quantified using ImageJ software. Percentages indicate oriented fibers accumulated in a range of ± 21° around the modal angle. Data are represented as mean ± s.d. (*n* = 10 random fields from 2 independent determinations). Scale bar, 50 µm. A representative image of picrosirius red staining from 10 analyzed images is shown for each condition.Atomic force microscopy (AFM) measurement of the elastic properties (apparent Young’s modulus, Εapp) of fibroblast‐derived ECMs. Each dot represents a specific Young's modulus obtained by fitting the corresponding individual force curve acquired on a determined point of the sample. A representative experiment from 2 independent experiments is shown. Scatter plots show mean ± SEM. The black bars represent the median and the interquartile range. ****P < 0.0001, two‐tailed Mann–Whitney test.

**Figure 2:**
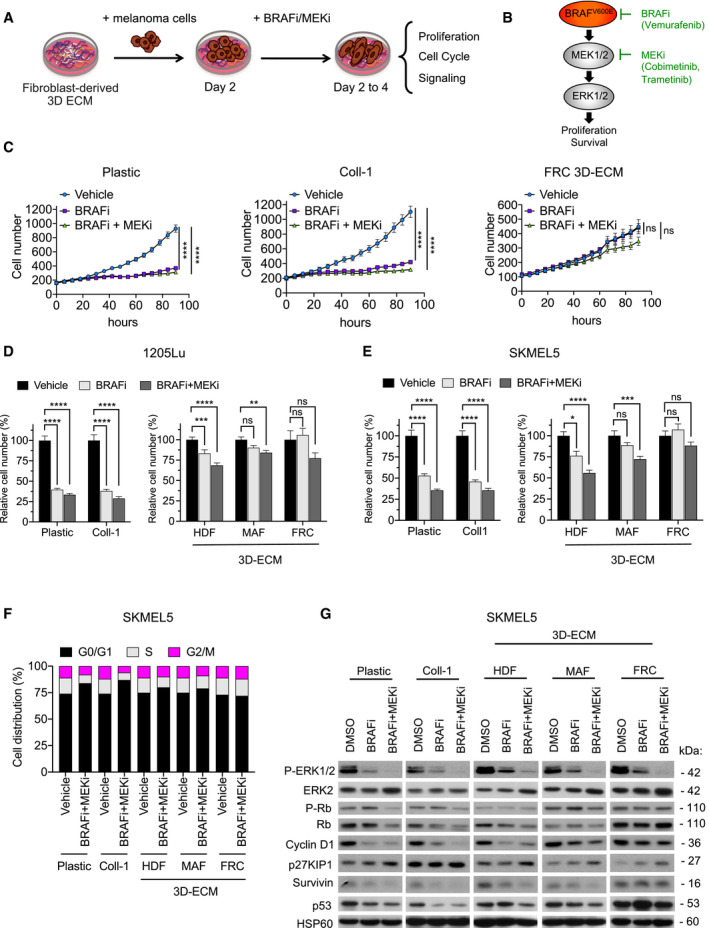
Fibroblast‐derived 3D ECM confers drug‐protective action to melanoma cells against anti‐BRAF^V600^ therapies AScheme of the ECM‐mediated drug‐protective assay.BIllustration of the BRAF^V600E^ pathway and the MAPK pathway inhibitors used in the study.CTime‐lapse imaging of proliferation of NucLight‐labeled 1205Lu cells plated on plastic (left panel), Coll‐1 (collagen 1; middle panel), or FRC‐derived ECM (right panel) and treated with vehicle, 5 µM BRAFi, or 2 µM BRAFi plus 0.01 µM MEKi using the IncuCyte ZOOM system. Each data point represents the mean of NucLight red nuclear objects per field ± SEM. ****P < 0.0001, two‐way ANOVA followed by Dunnett’s multiple comparisons test. Data are representative of *n* = 3 independent experiments.D, EQuantification of proliferation of 1205Lu (D) and SKMEL5 (E) cells plated for 48 h on plastic, Coll‐1, or the indicated fibroblast‐derived ECMs prior to a 96‐h treatment with vehicle, 5 µM BRAFi, or 2 µM BRAFi plus 0.01 µM MEKi. Cells were counted by Hoechst‐labeled nuclei staining. Data are represented as bar plots with mean ± SEM normalized to vehicle of 3 independent experiments. (D) **P = 0.015, ***P = 0.0009, and ****P < 0.0001; and (E) *P = 0.0204, ***P = 0.0006, and ****P < 0.0001, the Kruskal–Wallis test followed by Dunn’s multiple comparisons test.FFlow cytometry analysis of cell cycle distribution of SKMEL5 cells cultured on plastic, Coll‐1, or the indicated fibroblast‐derived ECMs and treated with vehicle or 2 µM BRAFi combined with 0.01 µM MEKi. The percentage of cells in different phases of the cell cycle is indicated.GImmunoblotting of protein extracts from SKMEL5 cells cultivated as described above on plastic, Coll‐1, or the indicated fibroblast‐derived ECMs in the presence or not of BRAFi or BRAFi/MEKi for 96 h, using antibodies against P‐ERK1/2, ERK2, or cell cycle markers (P‐Rb, Rb, cyclin D1, p27KIP1, survivin, and p53). HSP60, loading control. Source data are available online for this figure.

**Figure EV1:**
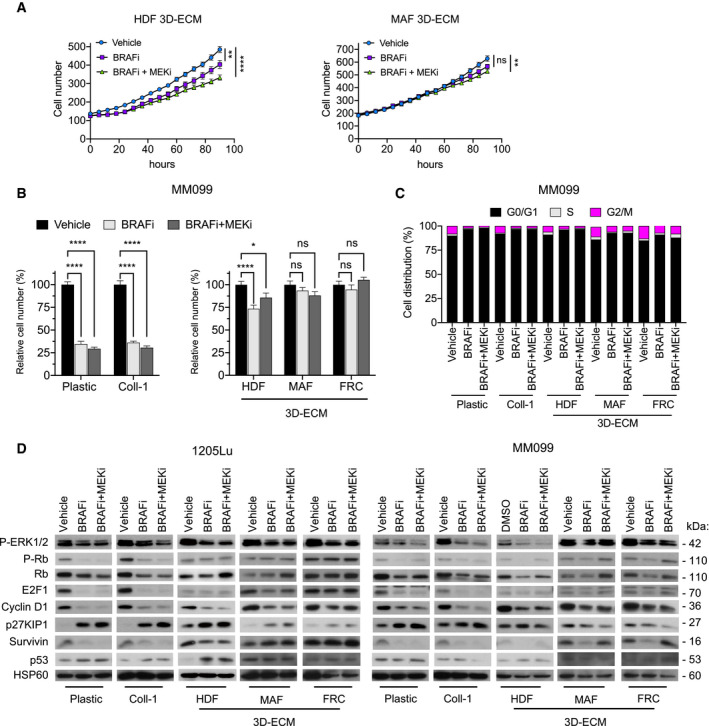
Fibroblast‐derived 3D ECM confers drug‐protective action to melanoma cells against anti‐BRAF^V600E^ therapies Time‐lapse imaging of proliferation of NucLight‐labeled 1205Lu cells using the IncuCyte ZOOM system. Cells were plated for 48 h on HDF‐ or MAF‐derived ECMs prior to a 96‐h treatment with vehicle, 5 µM BRAFi, or 2 µM BRAFi plus 0.01 µM MEKi. Each data point represents the mean of NucLight red nuclear objects per field ± SEM. **P = 0.0079 (left panel), **P = 0.0012 (right panel), and ****P < 0.0001, two‐way ANOVA followed by Dunnett’s multiple comparisons test. Data are representative of *n* = 3 independent experiments.Quantification of proliferation of MM099 short‐term melanoma cell cultures plated for 48 h on plastic, Coll‐1, or the indicated fibroblast‐derived ECMs prior to a 96‐h treatment with vehicle, 5 µM BRAFi, or 2 µM BRAFi plus 0.01 µM MEKi. Cells were counted by Hoechst‐labeled nucleus staining. Data are represented as bar plots with mean ± SEM normalized to vehicle of 3 independent experiments. *P = 0.0416 and ****P < 0.0001, two‐way ANOVA followed by Dunnett’s multiple comparisons test.Flow cytometry analysis of cell cycle distribution of MM099 cells cultured on indicated substrates and treated with vehicle, 5 µM BRAFi, or 2 µM BRAFi plus 0.01 µM MEKi. The percentage of cells in different phases of the cell cycle is indicated.Immunoblotting of protein extracts from 1205Lu cells (left panel) and MM099 cells (right panel) cultivated on indicated substrates in the presence or not of 5 µM BRAFi or 2 µM BRAFi plus 0.01 µM MEKi for 96 h, using antibodies against P‐ERK1/2, ERK2, P‐Rb, Rb, E2F1, survivin, p27KIP1, cyclin D1, and p53. HSP60, loading control.

To investigate the potential contribution of MMDR to targeted therapy in BRAF‐mutated melanoma cells, we employed an *in vitro* model based on live cell‐derived 3D ECMs. These matrices mimic many structural and biomolecular features, which are typically found *in vivo* (Cukierman *et al*, 2001). We selected human primary fibroblasts obtained from healthy individuals or MAFs isolated from patient metastatic melanoma biopsies (MAFs) in either the skin or lymph nodes (LN). The different fibroblast cultures were functionally tested in a 3D collagen matrix contraction assay, which showed that unlike human dermal fibroblasts (HDF), skin and LN MAF displayed actomyosin contractile activity. Similar to MAFs, LN normal fibroblasts known as fibroblastic reticular cells (FRC) are myofibroblast‐like cells (Fletcher *et al*, 2015), which also showed a high propensity to contract collagen (Fig 1A). Cell‐derived matrices were then generated and de‐cellularized, and their composition, architecture, and rigidity were analyzed using proteomic and microscopic approaches. In our experimental conditions, compared to HDF, skin and LN MAF, as well as FRC, produced and assembled a dense 3D ECM composed of oriented collagen and fibronectin fibers, as shown by picrosirius red and immunofluorescence staining of the ECMs (Fig 1B). Proteomic analysis of the different fibroblast‐derived ECMs further documented the molecular composition of these matrices, showing enrichment for several types of collagens and core matrisome components including glycoproteins, proteoglycans, and ECM regulators and ECM‐associated proteins (Appendix Fig S1). Atomic force microscopy (AFM) analysis of ECM stiffness revealed values for MAF and HDF matrices that were within the range of previous observations (Kaukonen *et al*, 2016; Fig 1C). We noticed that matrices generated from FRC and MAF were stiffer than HDF‐derived ECM. Collectively, these observations validate the use of our experimentally derived matrices for functional studies. Next, we tested the effectiveness of the fibroblast‐derived ECMs generated from HDF, FRC, or MAF to protect BRAF^V600E^‐mutated melanoma cells against the anti‐proliferative effect of MAPK pathway inhibition. We therefore developed a drug‐protective assay based on the culture of melanoma cells that stably expressed a fluorescent nuclear label, cultured on top of fibroblast‐derived ECMs (Fig 2A). Tumor cells were then treated with drugs targeting the mutant BRAF/MAPK pathway using BRAFi alone or in combination with MEKi (Fig 2B). Cell proliferation was monitored using live cell time‐lapse imaging and quantified by counting the number of fluorescent nuclei. Cell growth inhibition induced by BRAFi alone (vemurafenib) or in combination with MEKi (trametinib) was abrogated when 1205Lu melanoma cells were cultured on top of fibroblast‐derived ECMs, in sharp contrast to standard cell culture conditions where cells were plated either on plastic or on purified collagen 1 (Coll‐1) (Figs 2C and D, and EV1A). In line with the organization of the 3D matrices depicted in Fig 1, drug‐protective assays against BRAFi or BRAFi/MEKi combo‐therapy revealed that MAF‐ and FRC‐derived matrices display higher protective abilities compared to HDF‐derived ECMs and conferred increased protection (Figs 2D and EV1A). Similar protective effects were observed with the SKMEL5 cell line and the MM099 patient‐derived short‐term melanoma culture when these cells were plated on the different experimental ECM settings (Figs 2E and EV1B). These data suggest that MMDR relies on the topological and molecular features of the ECM. Cell cycle analysis in SKMEL5 and MM099 cells further showed that experimentally produced ECM from HDF, MAF, or FRC prevented the G0/G1 cell cycle arrest, induced by the BRAFi/MEKi cocktail in contrast to the cell culture conditions on plastic or Coll‐1‐plated dishes (Figs 2F and EV1C). The ECM therefore transmits signals that prevent the cytostatic action of MAPK pathway inhibitors. At the molecular level, ECM‐mediated therapeutic escape of SKMEL5, 1205Lu, and MM099 cells from BRAF pathway inhibition was associated with sustained levels of the proliferation markers phosphorylated Rb, cyclin D1, and survivin, and lower levels of the cell cycle inhibitor p27KIP1, although ERK1/2 phosphorylation was similarly decreased in the presence of the targeted drugs in all culture conditions (Figs 2G and EV1D). Immunoblot analysis also suggested that upon BRAF inhibition, the levels of proliferative markers were higher in melanoma cells on MAF‐ and FRC‐derived ECMs than on HDF‐derived ECMs. In contrast, no significant changes in p53 levels were observed. Together, these results indicate that fibroblasts assembled and remodeled matrices that provide a drug‐tolerant environment for BRAF mutant melanoma cell lines and short‐term cultures.

### Expression of the collagen receptors DDR1 and DDR2 in melanoma

Previous studies have demonstrated the critical role of ECM receptors belonging to the integrin family in drug resistance (Seguin *et al*, 2015). Moreover, BRAF inhibition has been described to generate a drug‐protective stroma with high β1 integrin/FAK signaling, as a result of the paradoxical action of BRAFi on MAF (Hirata *et al*, 2015). Yet, in our experimental conditions, we were unable to show a significant implication of the β1 integrin/FAK axis in drug protection conferred by fibroblast‐derived ECMs. Indeed, the addition of blocking β1 integrin antibodies and the depletion of FAK both failed to prevent the protective properties of fibroblast‐derived ECM against the growth and survival inhibitory signals induced by BRAFi (Appendix Fig S2).

**Figure 3:**
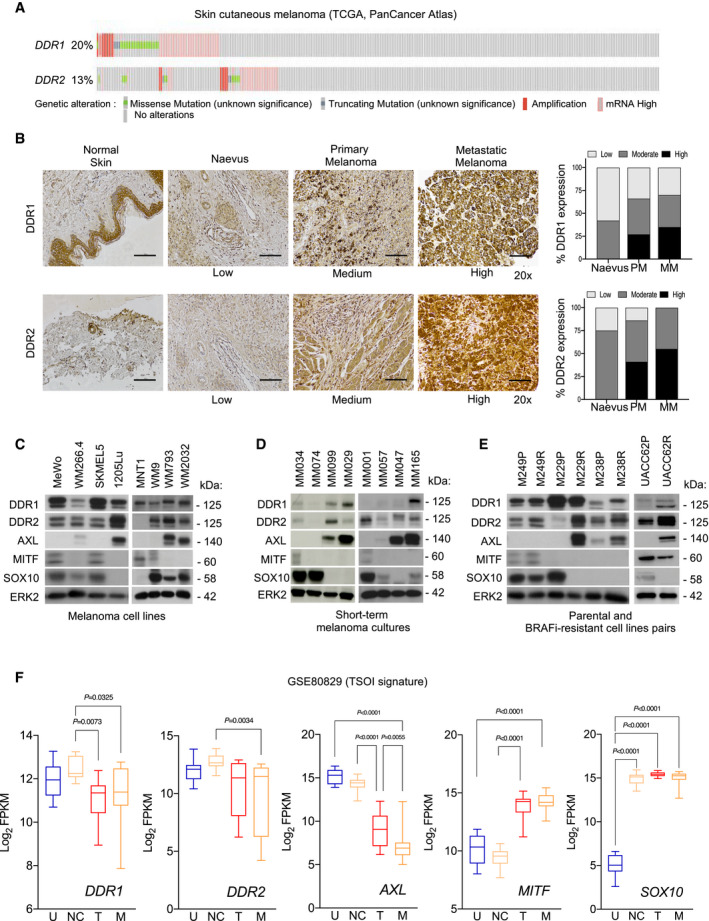
Expression of DDR1 and DDR2 in human melanoma AMeta‐analysis of 363 cutaneous melanoma from TCGA (skin cutaneous melanoma, PanCancer Atlas) (http://www.cbioportal.org/ ) showing the percentage of samples with genetic alterations in *DDR1* and *DDR2*. Cases with missense (green) and truncating (blue) mutations, amplification (red), and mRNA overexpression (pink) are indicated; gray, individual cases.BImmunohistochemical analysis of DDR1 and DDR2 levels on human melanoma tissue microarrays. Representative IHC images and quantification (right bar histograms) of DDR1 and DDR2 expression in normal skin, nevus, primary melanoma (PM), and lymph node melanoma metastases (MM). Scale bar, 100 µm. Histological scoring of the samples was performed in a blinded fashion. Samples were scored as low, medium, or high for DDR1 or DDR2 expression (nevus, *n* = 12; PM, *n* = 30; and MM, *n* = 20).C–EImmunoblotting of equal amounts of protein extracts from melanoma cell lines (C), patient‐derived short‐term cell cultures (D), or isogenic pairs of parental‐sensitive and BRAFi‐resistant cell lines (E) using antibodies against DDR1, DDR2, or markers of the melanoma cell differentiation AXL, MITF, or SOX10. ERK2, loading control.F
*DDR1* and *DDR2* levels increase in de‐differentiated melanoma cells. Box‐and‐whisker plots show *DDR1*, *DDR2*, *MITF*, *AXL,* and *SOX10* expression among four differentiation melanoma cell states (U, undifferentiated, *n* = 10; NC, neural crest‐like, *n* = 14; T, transitory, *n* = 12; and M, melanocytic, *n* = 17) (GSE80829). The expression of *AXL*, *MITF*, and *SOX10* is shown as control markers of cell differentiation. *n*, number of cell lines representative of each cell state. Central bars represent the median, and the whiskers, the 10^th^ to 90^th^ percentile of the boxplot. Multiple comparisons were performed using ordinary one‐way ANOVA. Source data are available online for this figure.

This finding prompted us to interrogate the contribution of other ECM receptors in targeted therapy resistance. Keeping in mind the elevated levels of fibrillar collagens found in fibroblast‐derived ECMs (Fig 1B; Appendix Fig S1), we examined the functional implication of the collagen tyrosine kinase receptors DDR1 and DDR2 (Shrivastava *et al*, 1997; Vogel *et al*, 1997). The analysis of TCGA datasets for cutaneous melanoma showed that the *DDR1* and *DDR2* genes were genetically altered in 20% and 13% of melanoma cases, respectively. Interestingly, a significant fraction of melanomas was found to be associated with an amplification of DNA copy number and higher mRNA levels of *DDR1* and *DDR2* (respectively 13% and 10% of samples) (Fig 3A). This is consistent with the notion that these collagen receptors may play an important role in melanoma pathogenesis. Immunohistochemical analysis of DDR1 and DDR2 expression in benign nevi and malignant primary and metastatic melanocytic skin lesions further showed that DDR1 and DDR2 levels significantly increased during melanoma progression, indicating that DDR1 and DDR2 may represent novel prognostic factors for melanoma (Fig 3B; Appendix Fig S3). We next examined the levels of DDR1 and DDR2 in a collection of melanoma cell lines and short‐term melanoma cultures in relation to the cell state differentiation markers MITF, SOX10, and AXL. DDR1 and DDR2 were both expressed in melanoma cell lines regardless of their differentiation of cell phenotype (Fig 3C). In patient‐derived short‐term melanoma cultures, higher DDR1 and DDR2 protein levels were detected in BRAF mutant MM099 and MM029 and NRAS mutant MM165 cells with the MITF^low^, SOX10^low^, and AXL^high^ de‐differentiated phenotype signature (Fig 3D). Moreover, higher levels of DDR2 were found to be associated with lower levels of the melanocytic marker MITF and higher levels of the drug‐resistant marker AXL in de‐differentiated mesenchymal‐like BRAFi‐resistant M229R, M238R, and UACC62R cells compared to their parental counterparts (Nazarian *et al*, 2010; Girard *et al*, 2020; Misek *et al*, 2020) (Fig 3E). The examination of public gene expression datasets of the melanoma differentiation signature confirmed that *DDR1* and *DDR2* levels were increased in the undifferentiated (U) and neural crest‐like (NC) cell subpopulations from the TSOI signature (Fig 3F; Tsoi *et al*, 2018) and in the invasive MITF^low^ cells from the HOEK signature (Appendix Fig S4; Widmer *et al*, 2012). De‐differentiated/undifferentiated melanoma cells display intrinsic resistance to MAPK pathway inhibition (Muller *et al*, 2014; Tsoi *et al*, 2018; Rambow *et al*, 2019). In line with this notion, we found that DNA amplification and elevated mRNA levels of *DDR1* negatively correlated to the activity of BRAF and MEK inhibitors in melanoma cell lines from the GDSC (Genomic of Drug Sensitivity in Cancer) (Appendix Fig S5). Together, these observations associate DDR expression with melanoma progression and with the invasive and therapy‐resistant phenotype.

### Targeting DDR impairs ECM‐mediated resistance to oncogenic BRAF pathway inhibition

**Figure EV2:**
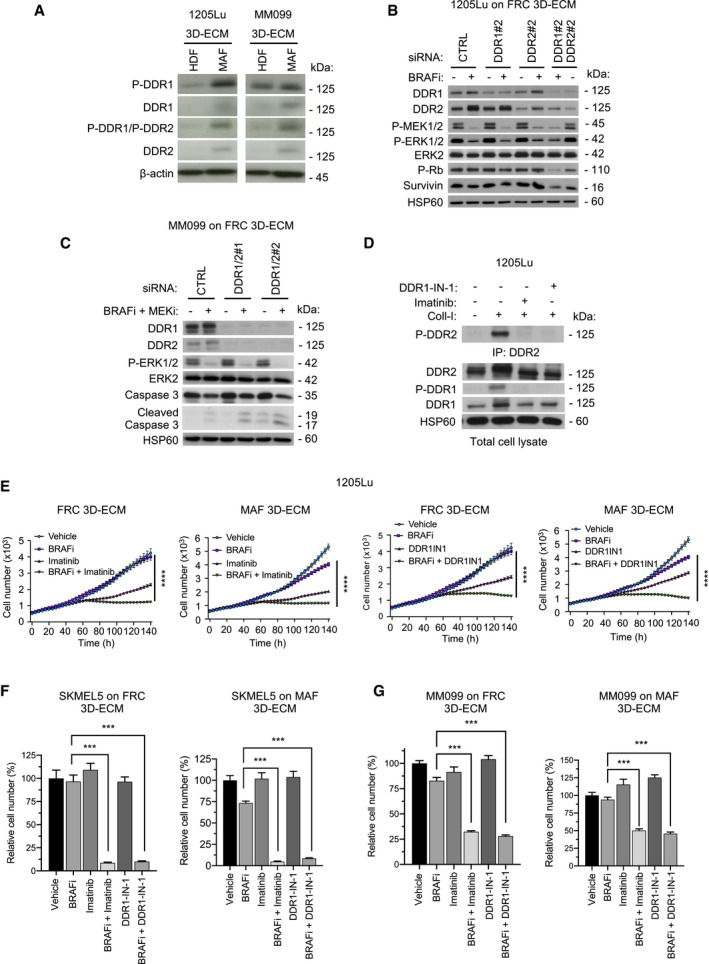
Knockdown and pharmacological inhibition of DDR1 and DDR2 abrogate ECM‐mediated resistance to BRAF^V600E^ pathway inhibition Immunoblotting of protein extracts from 1205Lu and MM099 cells cultivated on HDF‐ or MAF‐derived matrices using antibodies against P‐DDR1, P‐DDR1/P‐DDR2, DDR1, and DDR2. β‐actin, loading control.Immunoblotting of protein extracts of siCTRL‐, siDDR1#2‐, siDDR2#2‐, or siDDR1#2/siDDR2#2‐transfected 1205Lu cells plated on FRC‐derived ECM and treated or not with 5 µM BRAFi for 96 h, using antibodies against DDR1, DDR2, P‐MEK1/2, P‐ERK1/2, ERK2, P‐Rb, and survivin. HSP60, loading control.Immunoblotting of protein extracts of siCTRL‐, siDDR1#1/siDDR2#1, or siDDR1#2/siDDR2#2‐transfected MM099 melanoma short‐term cultures plated on FRC‐derived ECM and treated with vehicle or 2 µM BRAFi combined with 0.01 µM MEKi for 96 h, using antibodies against DDR1, DDR2, P‐ERK1/2, ERK2, and cleaved caspase‐3. HSP60, loading control.Immunoblot analysis of collagen I‐induced DDR1 and DDR2 tyrosine phosphorylation. 1205Lu cells were incubated with 10 µg/ml of Coll‐I (collagen I) in the presence or not of 7 µM imatinib or 1 µM DDR1‐IN‐1 for 18 h. After cell lysis, DDR2 phosphorylation was analyzed with anti‐P‐DDR2 following immunoprecipitation (IP) with anti‐DDR2 antibodies. DDR1 phosphorylation was analyzed in total cell lysates with anti‐P‐DDR1. HSP60, loading control.Time‐lapse imaging of proliferation of NucLight‐labeled 1205Lu cells plated for 48 h on FRC‐ or MAF‐derived ECMs prior to treatment with 5 µM BRAFi in the presence or not of 7 µM imatinib (left panels) or 1 µM DDR1‐IN‐1 (right panels) for the indicated times. Each data point represents the mean of NucLight red nuclear objects per field ± SEM. One experiment representative of 3 independent experiments is shown. ****P < 0.0001, 2‐way ANOVA followed by Dunnett’s multiple comparisons test.Quantification of proliferation of SKMEL5 cells plated for 48 h on FRC‐ (left panel) or MAF‐derived ECMs (right panel) prior to a 96‐h treatment with vehicle or 5 µM BRAFi in the presence or not of 10 µM imatinib or 5 µM DDR1‐IN‐1. Cells were counted by Hoechst‐labeled nucleus staining. Data are represented as bar plots with mean ± SEM normalized to vehicle. ***P = 0.0002, the Mann–Whitney test (*n* = 3).Quantification of proliferation of MM099 melanoma short‐term cultures plated for 48 h on FRC‐ (left panel) or MAF‐derived ECMs (right panel) prior to a 96‐h treatment with vehicle or 5 µM BRAFi in the presence or not of 10 µM imatinib or 3 µM DDR1‐IN‐1. Cells were counted by Hoechst‐labeled nucleus staining. Data are represented as bar plots with mean ± SEM normalized to vehicle. ***P = 0.0002, the Mann–Whitney test (*n* = 3).

**Figure 4:**
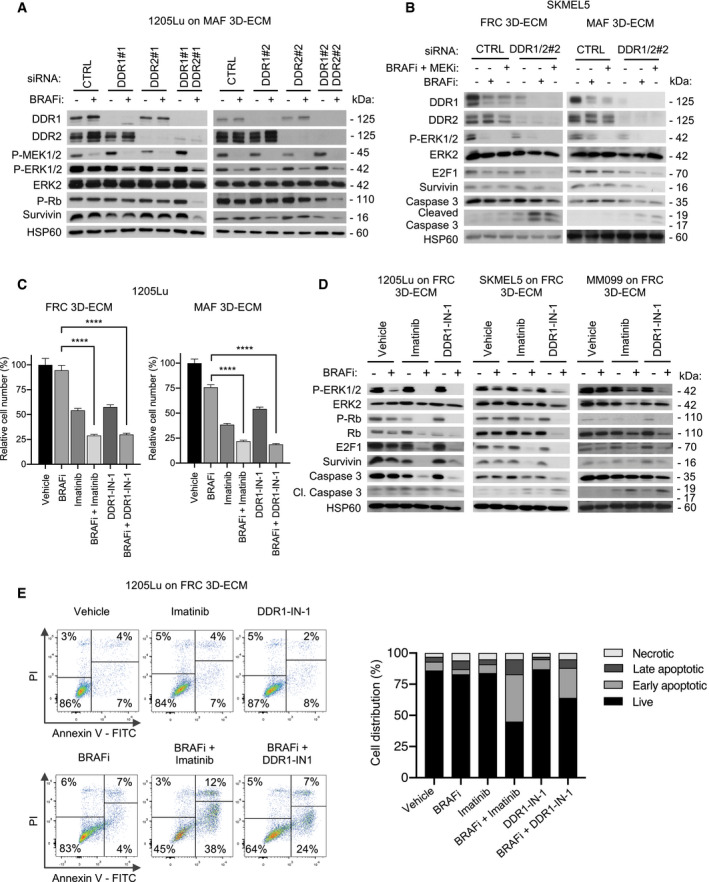
Inhibition of DDR1 and DDR2 by genetic or pharmacological approaches abrogates ECM‐mediated resistance to BRAF^V600^ pathway inhibition Immunoblotting of protein extracts from 1205Lu cells transfected with a siRNA control (CTRL) or two different sequences of siRNA (#1 and #2) directed against DDR1 or DDR2 alone or in combination prior to being cultivated on MAF‐derived ECM and treated or not with 5 µM BRAFi for 96 h, using antibodies against the indicated proteins. HSP60, loading control.Immunoblotting of protein extracts from SKMEL5 cells transfected with siCTRL or the combination of siDDR1#2 and siDDR2#2 prior to being cultivated on FRC‐ or MAF‐derived ECMs (left and right panels, respectively) and treated with vehicle, 5 µM BRAFi, or 2 µM BRAFi plus 0.01 µM MEKi, using antibodies against the indicated proteins. HSP60, loading control.Quantification of the time‐lapse imaging of the proliferation of NucLight‐labeled 1205Lu cells using the IncuCyte ZOOM system. Cells were plated for 48 h on FRC‐ or MAF‐derived ECMs prior to a 96‐h treatment with vehicle or 5 µM BRAFi in the presence or not of 7 µM imatinib or 1 µM DDR1‐IN‐1. The bar plots represent the mean normalized to vehicle of NucLight red nuclear objects per field ± SEM from 3 independent experiments performed in triplicate. ****P < 0.0001, two‐way ANOVA followed by Sidak’s multiple comparisons test.Immunoblotting of protein extracts from 1205Lu, SKMEL5, and MM099 cells plated for 48 h on FRC‐derived ECM prior to a 96‐h treatment with vehicle or 5 µM BRAFi in the presence or not of imatinib (7 µM for 1205Lu, 10 µM for SKMEL5 and MM099) or DDR1‐IN‐1 (1 µM for 1205Lu, 5 µM for SKMEL5, and 3 µM for MM099), using antibodies against the indicated proteins. HSP60, loading control.Flow cytometry analysis of cell death (Annexin V/PI labeling) in 1205Lu cells plated on FRC‐derived ECM and treated as above. Right bar plots show the distribution of cells (% of total) across the different forms of death. Source data are available online for this figure.

**Figure EV3:**
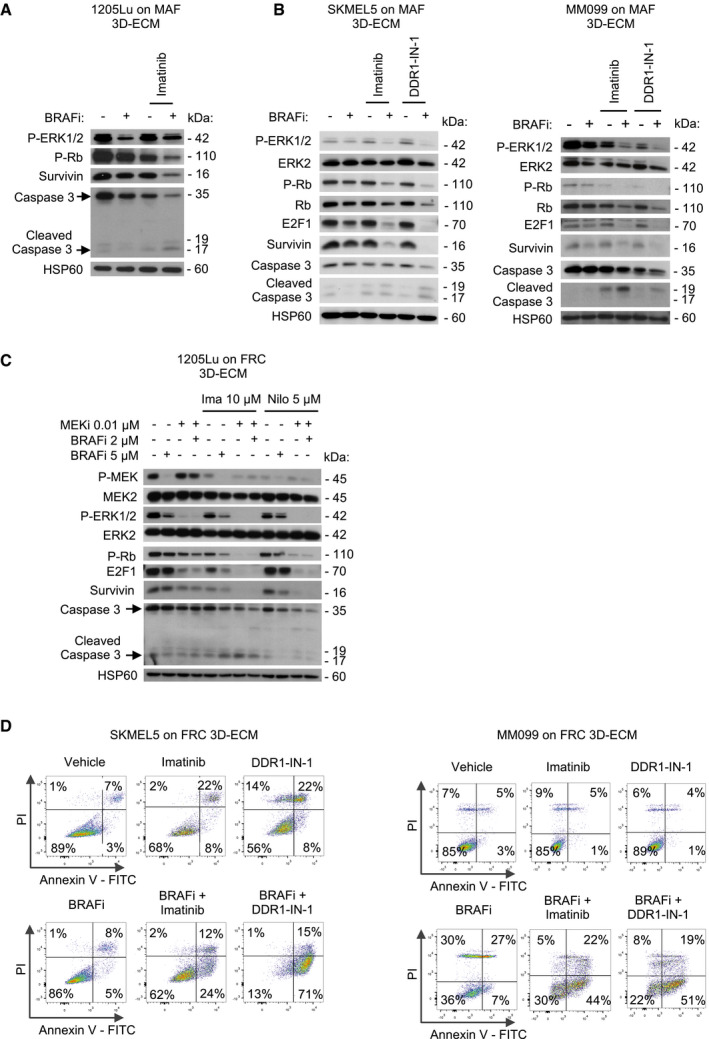
Pharmacological inhibition of DDR abrogates ECM‐mediated resistance to BRAF^V600E^ pathway inhibition and induces cell death Immunoblotting of protein extracts from 1205Lu cells cultivated on MAF‐derived ECM, treated or not with 5 µM BRAFi and/or 7 µM imatinib using antibodies against P‐Rb, P‐ERK1/2, survivin, or cleaved caspase‐3. HSP60, loading control.Immunoblotting of protein extracts from SKMEL5 cells (left panel) and MM099 melanoma short‐term cultures (right panel) cultivated on MAF‐derived ECM, treated or not with 5 µM BRAFi in combination with imatinib (10 µM) or DDR1‐IN‐1 (5 µM for SKMEL5 and 3 µM for MM099) using antibodies against P‐ERK1/2, ERK2, P‐Rb, Rb, E2F1, survivin, or cleaved caspase‐3. HSP60, loading control.Immunoblotting of protein extracts from 1205Lu cells cultivated on FRC‐derived ECM for 96 h in the presence of 5 µM BRAFi, 0.01 µM MEKi, or the combination of 2 µM BRAFi and 0.01 µM MEKi, in the presence or not of 10 µM imatinib or 5 µM nilotinib using anti‐P‐MEK1/2, P‐ERK1/2, P‐Rb, E2F1, survivin, or cleaved caspase‐3 antibodies (*n* = 2). HSP60, loading control.Flow cytometry analysis of cell death (Annexin V/PI labeling) in SKMEL5 cells (left) and MM099 cells (right) plated on FRC‐derived ECM and treated by the indicated drugs as described above. Data show the percentage of the different forms of cell death based on Annexin V/PI positivity.

Next, we compared how fibroblast‐derived ECMs modulate DDR phosphorylation, an event linked to their activity (Shrivastava *et al*, 1997; Vogel *et al*, 1997). Immunoblot analysis of lysates from 1205Lu and MM099 cells plated on MAF‐ and FRC‐derived ECMs indicated that MAF‐derived matrices have a stronger ability to increase the levels and phosphorylation of DDR1 and/or DDR2 compared to HDF‐derived matrices (Fig EV2A), supporting a functional implication of DDR in melanoma drug tolerance. Interestingly, while BRAF and/or MEK inhibition had no significant effect on DDR phosphorylation in 1205Lu cells cultivated on MAF‐derived ECM, MEK inhibition seemed to increase DDR1 expression levels (Appendix Fig S6). To address the contribution of DDR in MMDR, a siRNA approach was then used to target DDR1, DDR2, or both in melanoma cells cultured on MAF‐ or FRC‐derived ECMs, in the presence of BRAFi alone or in combination with MEKi. Immunoblot analysis showed specific DDR1 and DDR2 protein reduction after siRNA transfection using two different targeted sequences in BRAFi‐treated 1205Lu cells cultured on MAF‐ or FRC‐derived ECMs (Figs 4A and EV2B). Compared to the single knockdown, the simultaneous knockdown of DDR1 and DDR2 overcame MMDR to BRAF‐targeted therapy as revealed by decreased levels of cell proliferation markers, including phosphorylated Rb, survivin, and E2F1, in 1205Lu and SKMEL5 cells (Fig 4A and B). Importantly, depletion of both DDR1 and DDR2 enhanced the cytotoxic activity of co‐targeting BRAF/MEK as shown by the increased cleavage of apoptotic caspase‐3 that was detected in SKMEL5 and MM099 melanoma cells (Figs 4B and EV2C). DDR are druggable receptors targeted by imatinib, a tyrosine kinase inhibitor (TKI) initially developed as an ABL inhibitor, which also inhibits DDR activity with high efficacy (Day *et al*, 2008). Imatinib belongs to therapeutic molecules used in the clinic for the treatment of chronic myeloid leukemia and acute lymphoblastic leukemia with the Philadelphia chromosome (Druker *et al*, 2001). Enzymatic activities of DDR were also inhibited by other small molecules including DDR1‐IN‐1 (Kim *et al*, 2013). We first confirmed that imatinib and DDR1‐IN‐1 efficiently inhibited type I collagen‐induced DDR1 and DDR2 tyrosine phosphorylation in 1205Lu cells (Fig EV2D). Inhibition of DDR1/2 kinases by imatinib or DDR1‐IN‐1 suppressed the protective action of MAF‐ and FRC‐derived ECMs against BRAF inhibition, as evidenced by the significant decrease in 1205Lu cell proliferation, which was observed after co‐treatment with BRAFi and DDR1/2 inhibitors (Figs 4C and EV2E). Similar anti‐proliferative effects of DDR inhibitors were observed during drug‐protective assays performed in SKMEL5 and MM099 cells on MAF‐ or FRC‐derived ECMs (Fig EV2F and G). Mutant BRAF and DDR1/2 co‐targeting in the three melanoma cell lines plated on FRC‐ or MAF‐derived ECMs decreased levels of phosphorylated Rb, E2F1, and survivin, and induced caspase‐3 cleavage (Figs 4D and EV3A and B). Similar biochemical events were promoted by BRAFi or with the combined BRAFi and MEKi treatment, in the presence of nilotinib, a next‐generation BCR‐ABL inhibitor that is approved for the treatment of imatinib‐resistant patients and targeting DDR1/2 (Day *et al*, 2008; Fig EV3C). Induction of apoptosis in co‐treated melanoma cells was further confirmed using flow cytometry analysis of cell death markers (Figs 4E and EV3D).

**Figure 5:**
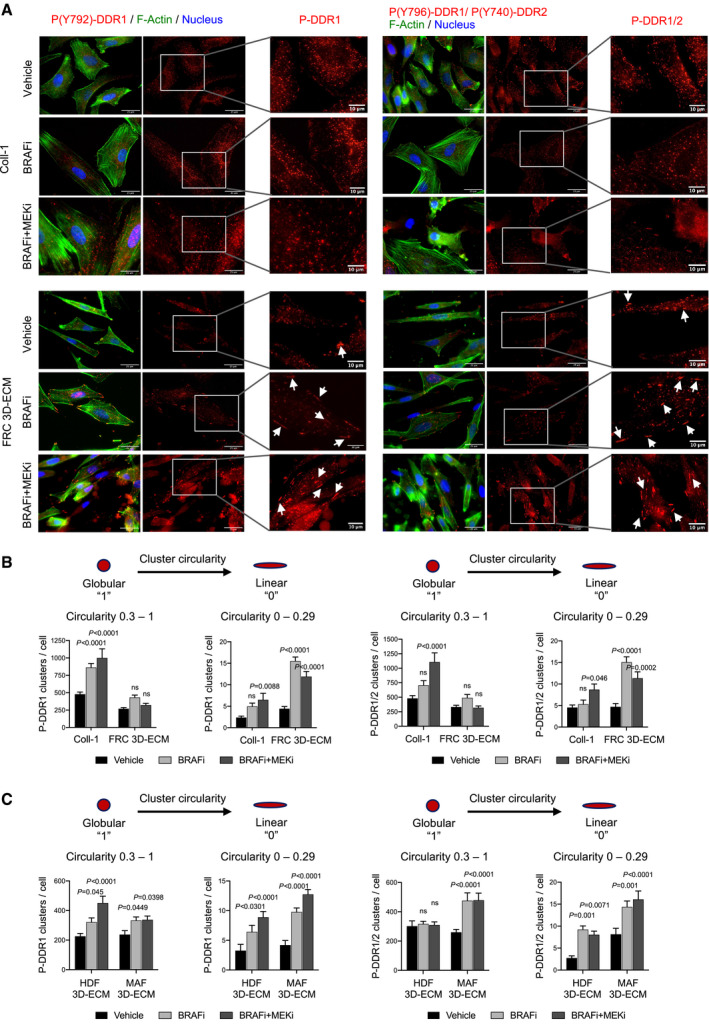
Interaction of melanoma cells with 3D ECMs induces the linear clustering of phosphorylated DDR upon BRAFi/MEKi treatment Representative images of 1,205 cells cultivated on collagen I (Coll‐I) or FRC‐derived ECM for 48 h prior to treatment with vehicle or 5 µM BRAFi or 2 µM BRAFi plus 0.01 µM MEKi for 96 h. Immunofluorescence for phospho‐DDR1 (P(Y792)‐DDR1) (red; left panels) and phospho‐DDR1/2 (P(Y796)‐DDR1/P(Y740)‐DDR2) (red; right panels), F‐actin (green), and nuclei (blue) is shown. Enlarged images of P‐DDR1 and P‐DDR1/2 immunostaining are shown. White arrows indicate P‐DDR1 and P‐DDR1/2 cell membrane linear clustering. Scale bar, 25 µm (enlarged images: scale bar, 10 µm).Quantification of globular versus linear clusters of phospho‐DDR1 (left panels) and phospho‐DDR1/2 (right panels) from immunofluorescence staining shown in (A) using ImageJ software. Prior to the quantification of DDR clusters, a “subtract background” function of ImageJ has been applied to all images. In order to quantify clusters, the IsoData threshold has been used. Clusters with circularity 0.3–1 have been defined as “globular”, and clusters with circularity 0–0.29 have been defined as “linear”. Data are from > 20 individual cells (*n* = 3). Error bars reflect mean ± s.d. Values for each treated condition are compared to the vehicle control. 2‐way ANOVA followed by Dunnett’s multiple comparisons test.Quantification of globular *versus* linear clusters of Phospho‐DDR1 and Phospho‐DDR1/2 from immunofluorescence staining shown in Fig EV4A of 1205Lu cells cultivated on HDF‐ or MAF‐derived ECM and treated with the indicated targeted drugs as described in (A). Data are from > 20 individual cells. Error bars reflect mean ± s.d. Values for each treated condition are compared to the vehicle control. 2‐way ANOVA followed by Dunnett’s multiple comparisons test. Source data are available online for this figure.

Recent studies described that collagen binding to DDR leads to their activation and clustering into filamentous membrane structures that are associated with collagen fibrils (Yeung *et al*, 2019). We thus examined the clustering and spatial distribution of phosphorylated DDR in melanoma cells plated on collagen I‐coated plastic dishes or on 3D ECMs, in response to oncogenic BRAF pathway inhibition. To detect DDR1 and/or DDR2, we used an antibody that specifically recognizes phosphorylated DDR1 at Y792 and an antibody that not only recognizes phosphorylated DDR2 at Y740 but also cross‐reacts with the phosphorylated DDR1 (Yeung *et al*, 2019). Immunofluorescence staining of P‐DDR1 and P‐DDR1/2 revealed that 1205Lu cells, which were cultured on purified collagen I, displayed a globular dot‐like distribution of these two receptors, whereas cells seeded on FRC‐derived 3D ECMs exhibited a fraction of P‐DDR distributed into linear membrane clusters (Fig 5A and B). Importantly, cell exposure to BRAFi or to BRAFi/MEKi combo‐therapy dramatically increased the proportion of P‐DDR1 and P‐DDR1/2 containing linear clusters, on FRC‐derived 3D ECM, as well as F‐actin remodeling (Fig 5A and B).

**Figure EV4:**
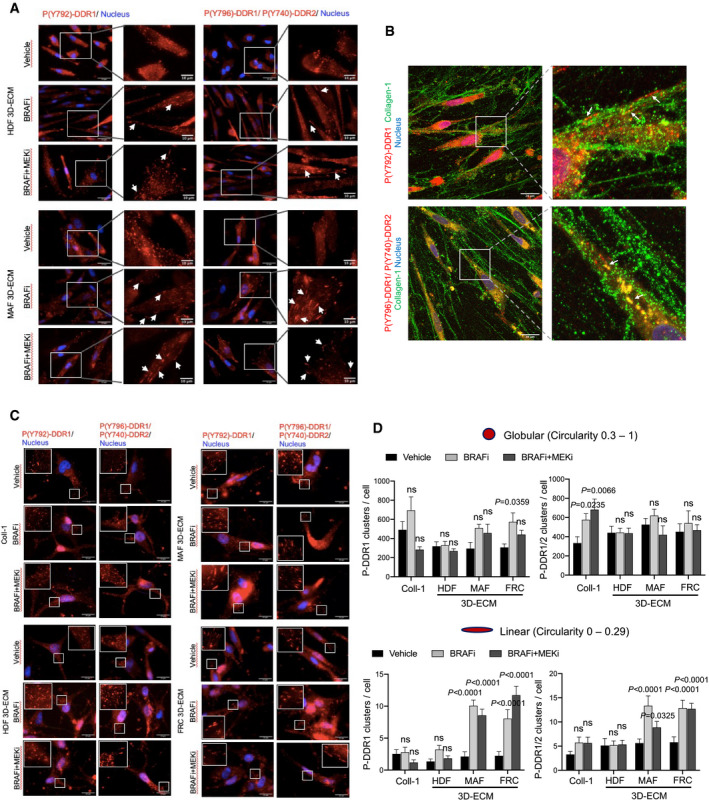
Interaction of melanoma cells with 3D ECM induces the clustering of phosphorylated DDR upon BRAFi or BRAFi/MEKi treatment Representative images of 1205Lu cells cultivated on HDF‐ or MAF‐derived ECM for 48 h prior to treatment with vehicle, 5 µM BRAFi, or 2 µM BRAFi combined with 0.01 µM MEKi for 96 h. Immunofluorescence for phospho‐DDR1 (P(Y792)‐DDR1) (red; left panels) or phospho‐DDR1/2 (P(Y796)‐DDR1/P(Y740)‐DDR2) (red; right panels) is shown. Nuclei (blue) were stained with DAPI. Enlarged images of P‐DDR1 and P‐DDR1/2 immunostaining are shown. White arrows indicate P‐DDR1 and P‐DDR1/2 cell membrane linear clustering. Scale bar = 25 µm (enlarged images, scale bar = 10 µm).Analysis of co‐localization of phospho‐DDR with collagen 1 in 1205Lu cells cultivated on MAF‐derived ECM and treated with BRAFi/MEKi. Immunofluorescence for phospho‐DDR1 (P(Y792)‐DDR1) (red; upper panels) and phospho‐DDR1/2 (P(Y796)‐DDR1/P(Y740)‐DDR2) (red; lower panels), collagen 1 (green), and nuclei (blue) is shown. Enlarged images are shown. White arrows indicate co‐localization (yellow fluorescence). Images were captured on Nikon Eclipse Ti confocal microscope at 60x magnification. Scale bar, 20 µm.Representative images of SKMEL5 cells cultivated on collagen I (Coll‐I) or on indicated fibroblast‐derived ECMs for 48 h prior to treatment with vehicle or 5 µM BRAFi or 2 µM BRAFi combined with 0.01 µM MEKi for 96 h. Immunofluorescence for phospho‐DDR1 (P(Y792)‐DDR1) (red; left panels) or phospho‐DDR1/2 (P(Y796)‐DDR1/P(Y740)‐DDR2) (red; right panels) is shown. Nuclei (blue) were stained with DAPI. Enlarged images of P‐DDR1 and P‐DDR1/2 immunostaining are shown. Scale bar = 25 µm (enlarged images, scale bar = 10 µm).Quantification of globular (left panels) and linear (right panels) clusters of phospho‐DDR1 and phospho‐DDR1/2 from immunofluorescence staining shown in (B) using ImageJ software. Prior to the quantification of DDR clusters, a “subtract background” function of ImageJ has been applied to all images. In order to quantify clusters, the IsoData threshold has been used. Clusters with circularity 0.3–1 have been defined as “globular”, and clusters with circularity 0–0.29 have been defined as “linear”. Data are from > 20 individual cells. Error bars reflect mean ± s.d. Values for each treated condition are compared to the vehicle control. 2‐way ANOVA followed by Dunnett’s multiple comparisons test.

Notably, the formation of linear clusters of P‐DDR1 and P‐DDR1/2 upon MAPK pathway inhibition is also significantly increased in cells plated on MAF‐derived 3D ECMs compared to cells cultured on HDF‐derived ECMs (Figs 5C and EV4A), and these clusters co‐localized with collagen fibers (Fig EV4B). Similar observations on phosphorylated DDR clustering were obtained with SKMEL5 melanoma cells (Fig EV4C and D). These data suggest that therapy‐induced cytoskeletal changes drive a linear clustering of phosphorylated DDR along collagen fibers and subsequently cause MMDR. These findings therefore suggest that DDR1 and DDR2 determine BRAF mutant melanoma cell responsiveness to BRAF‐targeted therapy, with regard to ECM features and that the drug‐tolerant action of DDR is dependent on their enzymatic activities and correlates with the formation of filamentous membrane structures.

### Pharmacological inhibition of DDR by imatinib improves targeted therapy efficacy, counteracts drug‐induced collagen remodeling, and delays tumor relapse

**Figure 6:**
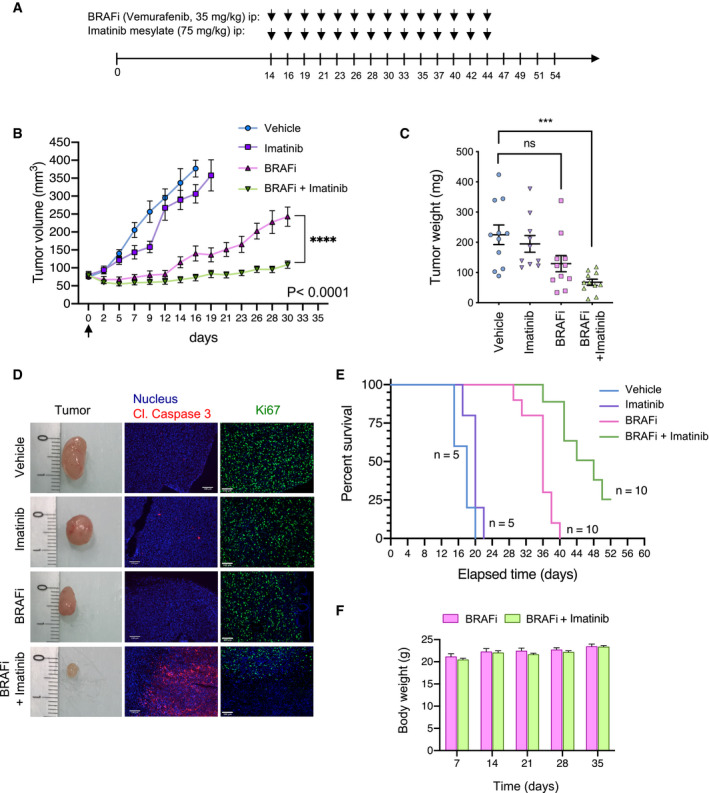
Targeting DDR by imatinib sensitizes melanoma tumors to BRAF^V600E^ inhibition Outline of the experimental setup and treatment regimens.1205Lu cells were s.c.‐inoculated into nude mice, and when tumors reached 75 mm^3^, mice were treated with the indicated mono‐ or combo‐therapy for 30 days. Graphs show tumor growth following treatment by indicated drugs. Data shown are mean ± SEM of tumor volume. Vehicle and imatinib groups, *n* = 10 tumors from 5 mice; vemu and vemu/imatinib groups, *n* = 20 tumors from 10 mice). ****P < 0.0001, two‐way ANOVA followed by Tukey's multiple comparisons test.Scatter plot graphs showing the tumor weight upon treatment by the indicated mono‐ or combo‐therapy. Error bars show mean ± SEM of tumor weight (*n* = 10–11 tumors from 6 mice per condition). ***P = 0.0002, the Kruskal–Wallis test followed by Dunn’s multiple comparisons test; ns, non‐significant.Immunofluorescence staining using anti‐cleaved caspase‐3 (red), anti‐Ki67 (green), and DAPI in tumor sections of 1205Lu‐derived xenografts from (B). Scale bar, 100 µm. A microphotograph of the tumor size in each treated group is shown (representative of *n* = 5–10 mice).Kaplan–Meier survival curves of mice treated with vehicle, imatinib, BRAFi, or BRAFi plus imatinib. Median time to progression was 18, 20, 36, and 48 days, respectively. Log rank (Mantel–Cox) for BRAFi *vs* BRAFi/imatinib mesylate. ****P < 0.0001 and hazard ratio (log rank): 0.2403 (95% CI of ratio, 0.08123–0.7106).Mouse body weight was measured at the indicated day. Data shown are mean ± SEM (*n* = 5–10 mice).

**Figure 7:**
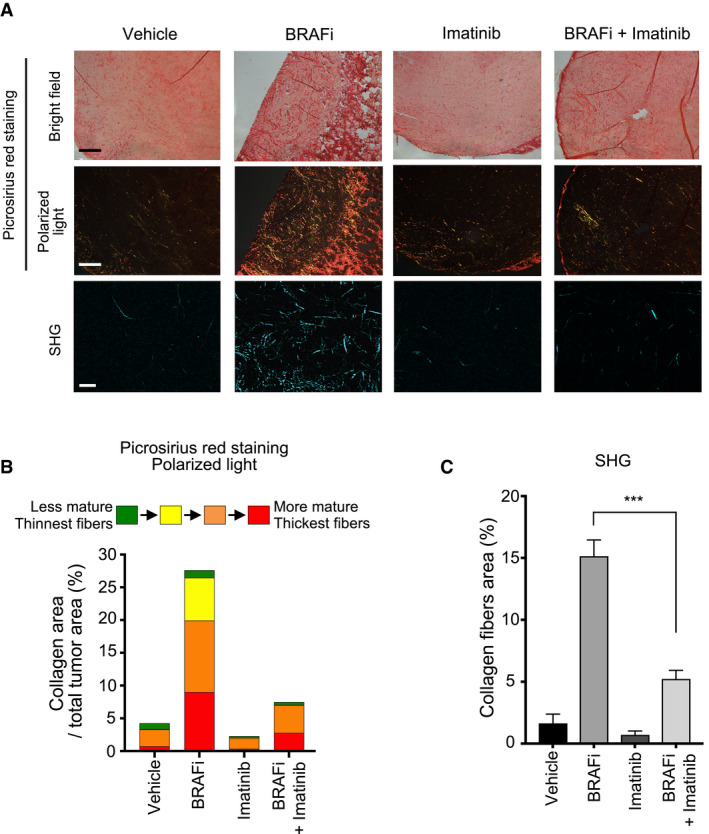
Imatinib normalizes collagen deposition and remodeling induced upon BRAFi treatment Sections of 1205Lu xenografts from Fig 6B were stained with picrosirius red and imaged under transmission light (upper panels) or polarized light (middle panels) (scale bar, 500 µm) or imaged by second harmonic generation (SHG) microscopy (lower panels) (scale bar, 50 µm) to examine collagen fiber network upon the mono‐ or combined regimens.Quantification of collagen maturity and fiber thickness in 1205Lu xenografts stained with picrosirius red using polarized light microscopy. Birefringence hue and amount of collagen fibers were quantified as a percent of total tissue area (2–4 fields per tumor section, *n* = 4 tumors per condition).Quantification of collagen fibers using SHG microscopy in tumor sections from (A). Error bars represent mean ± s.d. of 4 independent fields per section, *n* = 2 tumors per condition. ***P = 0.0002, the Mann–Whitney test.

The anti‐tumor activity of imatinib combined with the BRAFi vemurafenib was assessed in a preclinical xenograft melanoma model. BRAF‐mutated melanoma cells (1205Lu) were subcutaneously xenografted into nude mice (CDX model), which were exposed to BRAFi, imatinib, or BRAFi plus imatinib (Fig 6A). As expected, BRAF inhibition induced a rapid tumor reduction, whereas imatinib alone did not display a significant anti‐melanoma effect (Fig 6B). However, following 12 days, tumors treated with BRAFi alone had resumed growth, whereas the combination treatment with imatinib markedly delayed tumor relapse and led to a significant reduction in tumor volume (Fig 6B–D) and weight (Fig 6C). Immunohistochemical analysis of tumor samples further documented that in comparison with single regimens, the combined BRAFi and imatinib treatment dramatically reduced cell proliferation and led to apoptotic tumor cell death, as shown by the *in situ* expression of cleaved caspase‐3 and decreased Ki67 (Fig 6D). Consistent with these observations, the BRAFi and imatinib combination significantly increased the survival of melanoma‐bearing mice (Fig 6E) that were treated for 30 days, without apparent body weight loss or signs of toxicity throughout the study (Fig 6F). Seeing as increased collagen deposition has previously been described in melanoma xenografts upon treatment with BRAFi (Jenkins *et al*, 2015; Girard *et al*, 2020), we next investigated collagen content and fiber organization in tumor tissues from mice treated with the different regimens. Histochemical analysis showed that BRAFi treatment triggered a profound remodeling of the melanoma stromal ECM (Fig 7A), with marked increase in the collagen fibers’ area and thickness, which was suppressed by imatinib. Collagen color analysis under polarized light showed a decrease in mature orange and red fibers in tumors treated with the combined regimen, compared to the single‐agent treatment (Fig 7B). Finally, tumor imaging with second harmonic generation (SHG) confirmed the fibrillar nature of the collagen network that was rearranged upon BRAFi treatment but not upon the combined BRAFi and imatinib treatment (Fig 7A–C). These data suggest that treatment with imatinib counteracts the adverse effect of the targeted therapy on aberrant collagen deposition and organization, a process potentially contributing to drug resistance and relapse.

### MMDR involves a targetable pro‐survival NIK/IKKα/NF‐κB2 pathway

**Figure 8:**
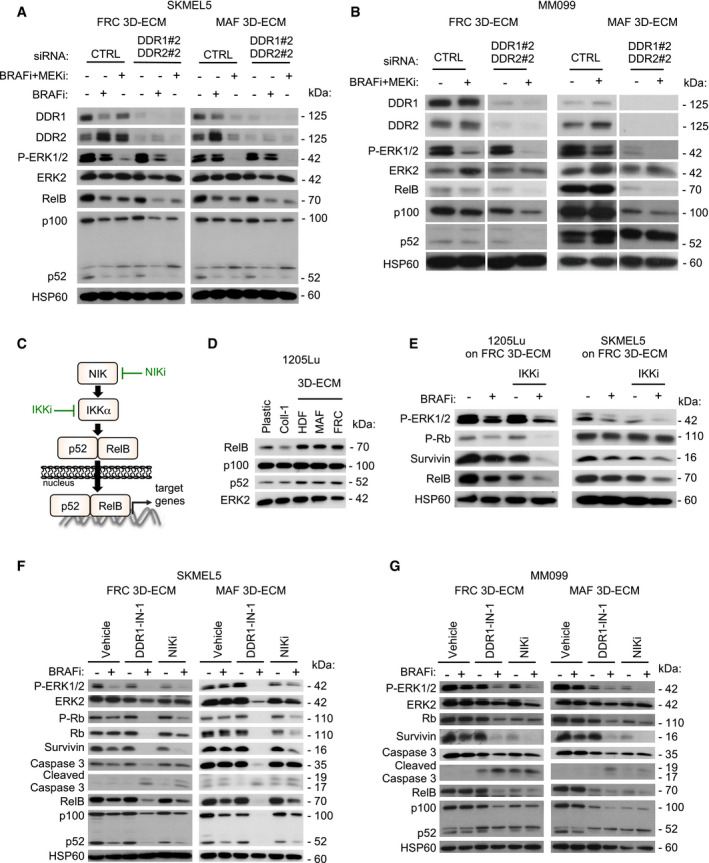
Targeting the non‐canonical NF‐κB2 pathway overcomes DDR‐dependent MMDR to BRAF^V600E^ pathway inhibition AImmunoblot analysis of protein extracts from siCTRL‐ or siDDR1/2‐transfected SKMEL5 cells plated on FRC‐ or MAF‐derived ECMs in the presence or not of 5 µM BRAFi, 2 µM BRAFi, plus 0.01 µM MEKi for 96 h using antibodies against DDR1, DDR2, P‐ERK1/2, ERK2, RelB, p100/p52, and HSP60 as loading control.BImmunoblot analysis of protein extracts from siCTRL‐ or siDDR1/2‐transfected MM099 short‐term cultures plated on FRC‐ or MAF‐derived ECMs in the presence or not of 2 µM BRAFi plus 0.01 µM MEKi for 96 h using antibodies as above. Note that data come from the same immunoblot.CIllustration of the non‐canonical p52/RelB NF‐κB2 pathway and inhibitors used in the study.DImmunoblot analysis of protein extracts from 1205Lu cells cultivated on plastic, Coll‐1, or indicated fibroblast‐derived ECMs for 48 h using antibodies against RelB, p100/p52, and ERK2 as loading control.EImmunoblot analysis of protein extracts from 1205Lu (left panels) or SKMEL5 (right panels) cells cultivated on FRC‐derived ECMs for 96 h in the presence of 5 µM BRAFi and/or a pan‐IKK inhibitor (IKKi, BMS‐345541 3 µM) using antibodies against P‐ERK1/2, P‐Rb, survivin, RelB, and HSP60 as loading control.F, GImmunoblot analysis of protein extracts obtained from SKMEL5 (F) or MM099 (G) cells plated on FRC‐ or MAF‐derived ECMs and treated with 5 µM BRAFi in combination or not with DDR1‐IN‐1 (5 µM for SKMEL5 and 3 µM for MM099) or 10 µM NIK inhibitor (NIKi) for 96 h. Antibodies against P‐ERK1/2, ERK2, P‐Rb, Rb, survivin, caspase‐3, cleaved caspase‐3, RelB, p100/p52, and HSP60 as loading control were used. Source data are available online for this figure.

**Figure EV5:**
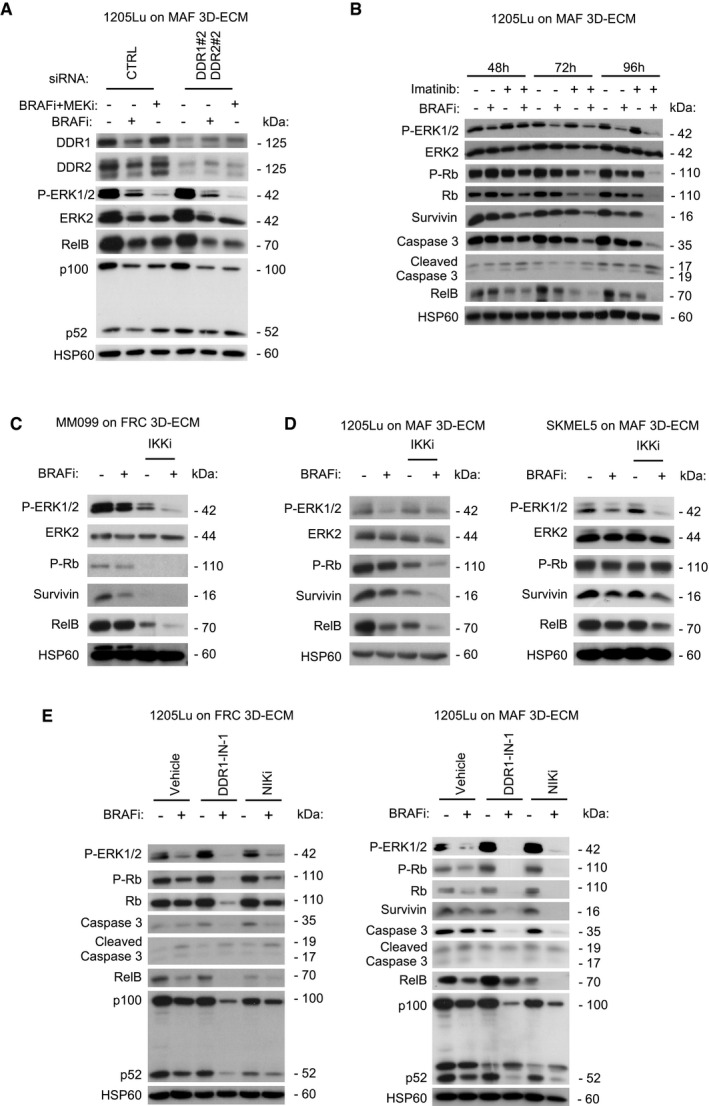
DDR and NF‐κB2 pathway targeting overcomes MMDR in response to BRAF^V600E^ inhibition Immunoblot analysis of protein extracts from siCTRL‐ or siDDR1/2#2‐transfected 1205Lu cells plated on MAF‐derived ECM in the presence or not of 5 µM BRAFi or 2 µM BRAFi and 0.01 µM MEKi for 96 h using antibodies against DDR1, DDR2, P‐ERK1/2, ERK2, RelB, and p100/p52. HSP60, loading control (*n* = 2).Immunoblotting of protein extracts from 1205Lu cells cultivated on MAF‐derived ECM treated with vehicle or 5 µM BRAFi and/or 7 µM imatinib, for the indicated time using antibodies against P‐ERK1/2, P‐Rb, Rb, survivin, caspase‐3, cleaved caspase‐3, and RelB. HSP60, loading control.Immunoblot analysis of protein extracts from MM099 melanoma short‐term cultures cultivated on FRC‐derived ECM for 96 h in the presence of BRAFi and/or a pan‐IKK inhibitor (IKKi, BMS‐345541 3 µM) using antibodies against P‐ERK1/2, P‐Rb, survivin, RelB and HSP60, loading control.Immunoblot analysis of protein extracts from 1205Lu (left panel) or SKMEL5 (right panel) cells cultivated on MAF‐derived ECM for 96 h in the presence of BRAFi and/or a pan‐IKK inhibitor (IKKi, BMS‐345541 3 µM) using antibodies against P‐ERK1/2, P‐Rb, survivin, RelB, and HSP60, loading control.Immunoblotting of protein extracts obtained from 1205Lu cells that were plated on FRC‐derived (left panel) and MAF‐derived (right panel) ECM and treated with 5 µM BRAFi in combination or not with 1 µM DDR inhibitor (DDR1‐IN‐1) or 10 µM NIK inhibitor (NIKi) for 96 h. Antibodies against P‐ERK1/2, P‐Rb, Rb, survivin, cleaved caspase‐3, RelB, p100/p52, and HSP60 as loading control were used.

Finally, we wished to better characterize at the molecular level the MMDR process that is promoted by collagen receptors DDR1 and DDR2. To identify MMDR‐related signaling pathways triggered by their tyrosine kinase activity, we performed a phospho‐kinase screening with protein extracts from melanoma cells cultured on fibroblast‐derived 3D ECMs or plastic, in the presence of BRAFi. Unfortunately, this approach did not reveal any significantly augmented kinase activity or increased phosphorylation of kinase substrates (Appendix Fig S7A and B). Moreover, consistent with the results of the phospho‐kinase screening, using immunoblot analysis, we did not observe by immunoblot analysis any changes in the phosphorylation of the AKT survival pathway in melanoma cells cultured under MMDR conditions (Appendix Fig S7C). On the contrary, compared to siCTRL cells, biochemical analysis of DDR‐silenced melanoma cells revealed that the concomitant depletion of the two receptors decreased the expression of RelB, NF‐κB2 precursor protein p100, and NF‐κB2 p52 processed form, in SKMEL5 and MM099 cells cultured in MMDR conditions, following exposure to BRAFi alone or in combination with MEKi (Fig 8A and B). A similar decrease in RelB and p100 was observed in DDR‐depleted 1205Lu cells, following treatment with targeted agents (Fig EV5A). Consistent with these results, pharmacological inhibition of DDR using imatinib that impaired melanoma MMDR by triggering cell cycle arrest and apoptosis decreased in a time‐dependent manner the expression of RelB in 1205Lu cells cultured in MMDR conditions (Fig EV5B).

**Figure 9:**
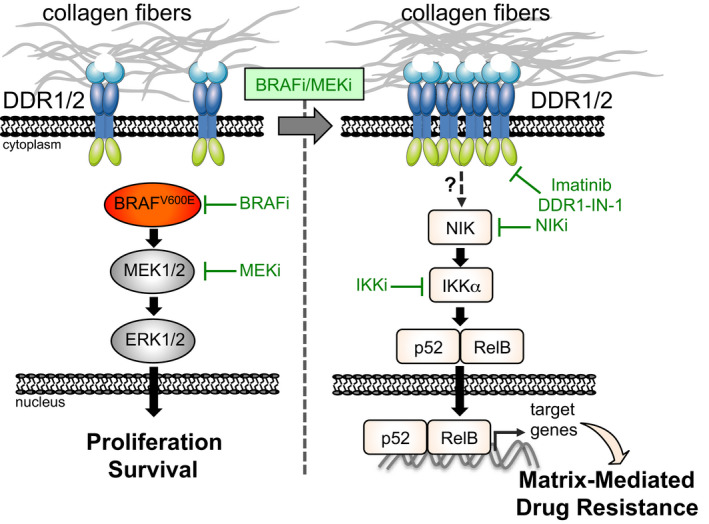
Model of DDR‐dependent matrix‐mediated drug resistance (MMDR) to MAPK‐targeting therapies in melanoma BRAF‐mutated melanoma cells adapt to BRAF/MEK inhibition by turning on a drug‐tolerant pathway that is initiated by collagen‐rich environments interacting with cancer cell DDR. Clustered DDR activates the non‐canonical NF‐κB2 (p52/RelB) pathway that is therapeutically targetable with clinically approved DDR inhibitor such as imatinib or with preclinically tested NIK inhibitors.

RelB and p52 are components of the non‐canonical NF‐κB2 pathway (Taniguchi & Karin, 2018), which is activated by the upstream kinases NIK (NF‐κB‐inducing kinase) and IKKα (IκB kinase) (Fig 8C). Interestingly, levels of RelB and p52 were found to be elevated in melanoma cells cultured on fibroblast‐derived ECMs compared to plastic and collagen‐coated dishes (Fig 8D). Using a pan‐IKK inhibitor BMS‐345541 (Figs 8E and EV5C and D) and a recently developed NIK inhibitor (NIKi; Mondragon *et al*, 2019; Figs 8F and G, and EV5E), we confirmed the implication of the NF‐κB2 pathway in MMDR, as illustrated by decreased cell cycle markers (pRb and survivin) and increased caspase‐3 cleavage, in BRAFi‐treated melanoma cells cultured on FRC‐ or MAF‐derived ECMs. Comparable decreased levels of p52 and RelB, as well as cell cycle markers, were observed following pharmacological targeting of DDR and NIK in MMDR conditions (Figs 8F and G, and EV5E). Together, our findings suggest that melanoma cell adaptation to oncogenic MAPK pathway inhibition involves the interaction of tumor cells with the mesenchymal stroma enriched in fibrillar collagens, thereby promoting DDR‐dependent activation of the NF‐κB2/RelB pathway (Fig 9).

## Discussion

Preventing melanoma resistance and relapse to targeted therapy remains a significant challenge for successful disease management. Emerging evidence suggests that stromal components of the tumor microenvironment, including the ECM, play a key role in establishing resistant niches by allowing melanoma cells to rapidly adapt and tolerate therapeutic drugs, before mutation‐driven resistance mechanisms are acquired (Meads *et al*, 2009; Smith *et al*, 2016; Rambow *et al*, 2019). Here, we describe a novel mechanism of adaptation and tolerance to oncogenic BRAF pathway inhibition, which involves the dynamic interaction of melanoma cells with the ECM. When comparing cell‐derived matrices produced by normal HDF, FRC, and MAF, we found that depending on fibroblast origin, properties of experimentally derived ECMs differ remarkably. MAF‐derived ECMs exhibit a higher level of fiber organization and increased stiffness compared to ECMs generated by normal dermal fibroblasts (HDF). This is in agreement with what has been previously demonstrated by others (Gopal *et al*, 2017; Kaukonen *et al*, 2017). Interestingly, we observed that FRC, which are resident lymph node fibroblasts, harbor some phenotypic and functional properties similar to MAF such as myofibroblast‐like properties. In this regard, FRC produce and remodel a stiff ECM enriched in fibrillar collagens. Consistent with this, we show that similar to MAF‐derived ECM, the ECM generated by FRC provides melanoma cells with increased protection from targeted drugs, compared to ECMs generated from HDF. However, it is important to note that in contrast to classic 2D culture conditions, HDF‐derived ECMs can confer protection against BRAF inhibition, indicating that MMDR is dependent on structural 3D ECM organization. Importantly, MMDR is described in three different BRAF mutant melanoma cells, regardless of their transcriptional phenotypic MITF/AXL signature.

Functionally, we demonstrate that collagen receptors DDR1 and DDR2 mediate MMDR to BRAF/MEK inhibitor therapy through a pro‐survival NIK/IKKα/NF‐κB2 pathway. We show that DDR knockdown or the inhibition of their catalytic activity impairs drug tolerance that is promoted by MAF‐ or FRC‐derived ECM and induces melanoma cell death. DDR1 and DDR2 are both expressed at different levels in the skin (Cario, 2018), particularly in the epidermis from which melanoma originates following the malignant transformation of melanocytes. Overlapping functions have been attributed to DDR1 and DDR2 in melanoma with regard to cancer cell growth and invasiveness. A recent study correlated high DDR1 expression in melanoma lesions with poor prognosis and showed that DDR1 controls melanoma cell invasion and survival (Reger de Moura *et al*, 2019). Other studies have reported that DDR2 depletion in melanoma cell lines reduced their invasive and metastatic abilities (Badiola *et al*, 2011; Poudel *et al*, 2015). Consistently, we observed augmented expression of the two receptors during the malignant transition from benign melanocytic lesions to metastatic melanoma and that their expression is linked with therapy‐resistant melanoma cell populations. Our data therefore suggest that the function of DDR1 and DDR2 also overlaps during the response to BRAF^V600^‐targeted therapy. Accordingly, knockdown of both receptors is required to fully prevent MMDR to oncogenic BRAF pathway inhibition.

Collagen abundance has recently been identified as an important contributor to melanoma cell phenotype switching through lineage‐specific microenvironment sensing (Miskolczi *et al*, 2018). Herein, we provide evidence for a role of collagen‐rich matrices during the adaptive phase of tolerance to targeted therapies. Interestingly, β1 integrin, another collagen receptor that plays a major role in EMDR (Seguin *et al*, 2015), has also been linked to adaptive responses to BRAF inhibition via fibronectin‐mediated activation of FAK, leading to MAPK pathway reactivation (Fedorenko *et al*, 2015; Hirata *et al*, 2015). Contrary to β1 integrin, engagement of DDR by collagens mediates drug tolerance in the absence of MAPK/ERK pathway reactivation. This suggests that DDR participate in the early response to BRAF inhibition through a pathway distinct from that of β1 integrin. Additionally, we show that fibroblast‐derived ECMs promote phospho‐DDR1/2 clustering into linear membrane structures aligned with collagen and resembling those described by others (Agarwal *et al*, 2007; Corcoran *et al*, 2019; Yeung *et al*, 2019). Importantly, receptor linear clustering is enhanced upon melanoma cell treatment with targeted therapies, thus making the involvement of this process during the early phase of drug adaptation highly probable. In addition, linear clustering of phospho‐DDR upon BRAF pathway inhibition is favored on MAF‐ and FRC‐derived ECMs compared to HDF‐derived ECM, correlating with the topological organization of collagen fibers and their drug‐protective effect. The exact mechanism underlying the induction of DDR clustering following MAPK pathway inhibition is, however, unknown.

Biochemical studies revealed that fibroblast‐derived matrices enhance the activation of the non‐canonical NF‐κB2 pathway that account for most of the melanoma cell tolerance to BRAF/MEK inhibition. The NF‐κB2/p52/RelB pathway represents a major alternative route of NF‐κB signaling (Taniguchi & Karin, 2018), which has been involved in drug resistance in myeloma (Landowski *et al*, 2003) and prostate cancer (Nadiminty *et al*, 2013). In melanoma, the NF‐κB2 pathway is upregulated compared to melanocytes and prevents melanoma senescence (De Donatis *et al*, 2016). In this study, we demonstrate that suppression of DDR signaling impairs MMDR by reducing NF‐κB2/p52/RelB expression. In addition, inhibition of NIK or IKKα, two upstream activators of the NF‐κB2 pathway (Taniguchi & Karin, 2018), also abrogates ECM‐mediated resistance to BRAF pathway inhibition. Further supporting a potential role for the NF‐κB2 pathway in melanoma resistance to targeted therapy is the observation that the drug‐resistant undifferentiated and neural crest‐like subpopulations display a strong enrichment for the NIK/NF‐κB signaling pathway (Tsoi *et al*, 2018). Precisely how DDR interact with the NIK/IKKα/NF‐κB2 pathway remains to be determined. The adaptive response of melanoma cells to BRAF inhibition implicates force‐induced actomyosin cytoskeletal remodeling (Kim *et al*, 2016; Girard *et al*, 2020; Orgaz *et al*, 2020). In addition, DDR1 clustering and collagen mechanical reorganization have been shown to involve interactions with myosin IIA, a key component of the actomyosin network (Coelho *et al*, 2017). In cancer cells, the classical NF‐κB pathway is activated by mechanical cues and RhoA‐ROCK‐myosin II signaling (Sero *et al*, 2015). We can therefore speculate that BRAFi/MEKi‐driven cytoskeletal reorganization and DDR linear clustering along with collagen fibrils regulate the NIK/IKKα/NF‐κB2 pathway by increasing actomyosin tension. Another possibility is the recruitment of NIK/IKKα to the DDR signaling platforms, leading to their enzymatic activation, p52/RelB regulation, and MMDR.

Our recent report revealed that increased collagen deposition and tumor stiffening is an early response to BRAF inhibition to create a drug‐protective niche (Girard *et al*, 2020). In this study, DDR inhibition by the clinically approved TKI imatinib improved the action of BRAFi on melanoma treatment by delaying tumor relapse, normalizing the collagen network, and increasing mouse survival. Thus, this combination strategy could be effective in a clinical application as it allows the suppression of the stromal fibrotic‐like reaction induced by oncogenic BRAF pathway inhibition and prevents tumor relapse. Our findings support a model in which BRAF‐targeted drugs fuel a self‐feeding mechanism involving collagen‐bound DDR signaling platforms, responsible for collagen network remodeling and drug tolerance. Another issue highlighted by our *in vivo* drug response melanoma model is the possible implication of stromal DDR in the therapeutic response. Consistent with this, a major role of CAF‐derived DDR2 in collagen fiber organization and breast cancer metastasis has been reported (Corsa *et al*, 2016). DDR signaling might therefore influence both cancer and stromal cells during tumor adaptation to BRAF inhibition.

Our work adds to the emerging notion that DDR are becoming attractive targets in anti‐cancer therapies. Inhibiting DDR1 or DDR2 with nilotinib (Jeitany *et al*, 2018), dasatinib (Hammerman *et al*, 2011; von Massenhausen *et al*, 2016), or other non‐approved inhibitors (Ambrogio *et al*, 2016; Grither & Longmore, 2018) was shown to decrease tumorigenicity, invasion, or metastasis of several carcinomas (Corsa *et al*, 2016). Importantly, a recent study in melanoma reported that targeting DDR2 with dasatinib enhances tumor response to anti‐PD‐1 immunotherapy (Tu *et al*, 2019). Together, this study and our work point to the critical role of collagen receptors DDR in regulating the immune and mesenchymal stroma and the response to current therapies in melanoma.

In summary, our findings reveal that interaction of matrix collagen fibers with DDR favors the emergence of drug‐tolerant melanoma cells, during the initial phase of adaptation to MAPK‐targeting therapies. We also provide evidence that targeting MMDR with clinically approved DDR inhibitors may represent an attractive salvage strategy to overcome resistance to oncogenic BRAF inhibition. This combinatorial approach may thus be beneficial for melanoma patients treated with targeted therapies.

## Materials and Methods

### Melanoma cells, reagents, and antibodies

Melanoma cell lines were obtained as previously described (Tichet *et al*, 2015; Didier *et al*, 2018; Rathore *et al*, 2019). Isogenic pairs of vemurafenib‐sensitive (P) and vemurafenib‐resistant (R) cells (M229, M238, M249) were provided by R.S. Lo (Nazarian *et al*, 2010). The isogenic pair of vemurafenib‐sensitive (UACC62P) and vemurafenib‐resistant (UACC62R) cells was provided by R. Neubig (Misek *et al*, 2020). Short‐term cultures of patient melanoma cells were kindly provided by J.C. Marine and are described elsewhere (Verfaillie *et al*, 2015; Wouters *et al*, 2020). Melanoma cells were cultured in Dulbecco's modified Eagle’s medium (DMEM) supplemented with 7% fetal bovine serum (FBS) (HyClone) and 1% penicillin/streptomycin solution. Cell lines were used for experiments within 6 months of thawing. To guarantee cell line authenticity, cells were expanded and frozen at the lowest possible passages and used for a limited number of passages after thawing. Cells were routinely tested for the expression of melanocyte lineage proteins such as MITF. All cell lines were routinely tested for the absence of mycoplasma using PCR. For live imaging and red nuclear labeling, 1205Lu cells were transduced with NucLight Red Lentivirus Reagent (Essen Bioscience) and selected with puromycin (1 μg/ml, Sigma‐Aldrich).

Culture reagents were purchased from Thermo Fisher Scientific. BRAFi (PLX4032, vemurafenib), MEKi (GSK1120212, trametinib, and GDC‐0973/RG7420, cobimetinib), dual RAFi/MEKi (RO5126766), DDR1‐IN‐1, nilotinib, and IKK inhibitor, BMS‐345541, were obtained from Selleckchem. Imatinib mesylate was purchased from Enzo Life Sciences. The NIK inhibitor was described before (Mondragon *et al*, 2019). FAK inhibitor PF573228 was from Tocris Bioscience. An equal amount of DMSO was used as the vehicle control. Tissue culture grade collagen I (#A1064401) used for coating wells and plates was obtained from Thermo Fisher Scientific. Collagen I (#354249) used for 3D applications was obtained from Corning. Matrigel (#E1270) and all other reagents were obtained from Sigma‐Aldrich unless otherwise stated. β1 integrin blocking antibody (clone AIIB2) and the control isotype were purchased from Merck Millipore. Information on antibodies used in this study is provided in Appendix Table S1.

### Isolation and culture of primary fibroblasts and MAFs

Human dermal fibroblasts were isolated and maintained as previously described (Robert *et al*, 2006). Human lymphatic fibroblasts (FRC#2 and FRC#5) were purchased from ScienCell Research Laboratories. Metastatic melanoma clinical specimens were obtained with written informed consent from each patient, and studies were approved by the hospital ethics committee (Nice Hospital Center and University Côte d’Azur). The experiments conformed to the principles set out in the WMA Declaration of Helsinki and the Department of Health and Human Services Belmont Report. Briefly, the tissue sample was cut into small pieces and digested using collagenase/dispase. Following filtration of the large debris, the solution was serially centrifuged and the final pellet was resuspended in DMEM supplemented with 10% FBS and seeded in a tissue culture dish. After 30 min, the fibroblasts adhered to the dish, while other cellular types remained in suspension. Verification of MAF was determined by flow cytometry using CAF markers such as β1 integrin, PDGFRβ, FAPα, and αSMA.

Fibroblasts were cultured in fibroblast medium (ScienCell) supplemented with 10% FBS, 1% fibroblast growth supplement (FGS), and 1% penicillin/streptomycin solution. All experiments were performed with fibroblasts until passage 10.

### Fibroblast‐derived 3D ECM and MMDR assay

3D de‐cellularized ECMs were generated as previously described (Beacham *et al*, 2007). Briefly, fibroblasts were seeded in gelatin‐coated tissue culture dishes, cultured for 8 days in a complete medium, and treated with 50 μg/ml ascorbic acid every 48 h. Cells were then washed with PBS, and ECMs were de‐cellularized using a prewarmed extraction buffer for 2 min (PBS 0.5% Triton X‐100, 20 mM NH_4_OH). Matrices were then gently washed several times with PBS.

For MMDR assays, melanoma cells were seeded on top of the de‐cellularized 3D ECMs for 48 h at 37°C in 5% CO_2_, and cultured in a complete medium for a further 96 h in the presence of the various inhibitors, as indicated in the figure legends. Cells were detached and fixed in 70% ethanol or lysed in lysis buffer for cell cycle and immunoblot analysis, respectively.

### Matrix‐remodeling assay

5 × 10^4^ fibroblasts were embedded in 100 µl of collagen I/Matrigel mix (final concentration of collagen I: 4 mg/ml and Matrigel: 2 mg/ml), seeded in a glass‐bottom 96‐well plate (MatTek), and maintained in DMEM supplemented with 10% FBS for 6 days. The gel area was measured using ImageJ software, and the gel contraction was calculated using the formula 100 × (well diameter−gel diameter)/well diameter as previously described (Albrengues *et al*, 2014).

### RNAi studies

Non‐targeting control, DDR1#1 (VHS50139), DDR1#2 (HSS187878), DDR2#1 (HSS107350), and DDR2#2 (HSS107352), and FAK (PTK2) siRNA duplexes were purchased from Thermo Fisher Scientific. Transfection of siRNA was carried out using Lipofectamine RNAiMAX (Thermo Fisher Scientific), at a final concentration of 50 nM. Unless stated otherwise, cells were assayed at 2 or 4 days post‐transfection.

### Immunoblot and immunoprecipitation

Whole‐cell lysates were prepared using lysis buffer containing 50 mM HEPES, 150 mM NaCl, 1.5 mM MgCl_2_, 1 mM EDTA, 10% glycerol, and 1% Triton X‐100 supplemented with protease and phosphatase inhibitors (Pierce) and briefly sonicated. Proteins were separated using SDS–PAGE and were transferred onto PVDF membranes (GE Healthcare Life Sciences) for immunoblot analysis. Membranes were incubated with the primary antibody overnight, washed, and then incubated with the peroxidase‐conjugated secondary antibody. Antibodies and working dilution used are listed in Appendix Table S1. Blots were developed with a chemiluminescence system (GE Healthcare Life Sciences).

For immunoprecipitation (IP) assays, cells treated with 10 μg/ml of collagen I for 18 h with the indicated inhibitors were lysed as described above, then incubated overnight at 4°C with rocking in lysis buffer containing Protein G Sepharose beads (Merck) and an antibody directed against DDR2. Beads were then washed 3 times with 20 mM HEPES, 150 mM NaCl, 10% glycerol, and 0.1% Triton X‐100 supplemented with protease and phosphatase inhibitors. IP products were separated using SDS–PAGE and subjected to immunoblot analysis.

### Proliferation assay

Real‐time analysis of cell growth was carried out using the IncuCyte™ ZOOM imaging system (Essen Bioscience). 1205Lu melanoma cells stably expressing the nuclear fluorescent label red NucLight reagent were seeded in triplicate in a complete medium (15 × 10^3^ cells/well in 12‐well plates) on fibroblast‐derived 3D ECM, in collagen I‐coated wells, or in uncoated plastic wells and treated with the indicated drugs. Phase‐contrast and red immunofluorescent images were taken every 6 h over a 5‐day period (4 images per well at ×4 magnification). Cell proliferation was quantified by counting the number of fluorescent nuclei over time, which was calculated as the cell growth rate. Growth curves were generated using the GraphPad Prism 8 software. Alternatively, cell proliferation was analyzed by counting cells with Hoechst‐stained nuclei.

### Flow cytometry

Cell cycle profiles were determined using flow cytometry analysis of propidium iodide (PI)‐stained cells as previously described (Didier *et al*, 2018). Melanoma cells were cultured on top of fibroblast‐derived ECMs, collagen I‐coated wells, or uncoated plastic wells and were treated with the indicated drugs for 96 h. Cells were then washed, fixed in 70% ethanol, and incubated at −20°C for 24 h. Finally, cells were stained for 20 min at 37°C in buffer containing 40 μg/ml PI and 20 μg/ml ribonuclease A. Cell cycle profiles were collected using the FACSCanto II (Becton Dickinson). Cell death was evaluated following staining with Annexin V/PI (eBioscience) and analyzed by flow cytometry as described (Didier *et al*, 2018).

### Immunofluorescence and microscopy

Cells were grown on type I collagen‐ (0.14 mg/ml) or fibroblast‐derived ECM‐coated glass coverslips. Following BRAFi or BRAFi+MEKi treatment, cells were rinsed with PBS, fixed in 4% paraformaldehyde (PFA), and incubated for 1 h in PBS containing 10% normal goat serum (Cell Signaling), and then incubated overnight with the indicated primary antibodies that were diluted in PBS containing 2% normal goat serum. F‐actin was stained with Alexa Fluor 488 phalloidin (1:100; Thermo Fisher Scientific), and nuclei were stained with DAPI. Following incubation with Alexa Fluor‐conjugated secondary antibodies, coverslips were mounted in ProLong antifade mounting reagent (Thermo Fisher Scientific). Images were captured using a wide‐field microscope (Leica DM5500B, at ×63 magnification). Cell area and globular *versus* linear clusters of cells (*n* > 20) were determined and quantified using ImageJ software. For quantification of globular versus linear clusters of phospho‐DDR1/2, a “subtract background” function of ImageJ was applied to all images. In order to quantify clusters, the IsoData threshold was used. Clusters with circularity 0.3–1 were defined as “globular” and clusters with circularity 0 to 0.29 as “linear”. Following fixation and incubation with primary antibodies and Alexa Fluor‐conjugated secondary antibodies, de‐cellularized matrices on coverslips were mounted in ProLong antifade reagent. Images were captured using a wide‐field microscope (Leica DM5500B, at ×40 magnification). The orientation of fibronectin and collagen fibers was assessed in the immunofluorescence images using ImageJ software. Data were plotted as frequency of distribution. For co‐localization analyses of collagen 1 and phospho‐DDR1/2, an anti‐collagen type I mouse monoclonal antibody (Sigma, C2456) was used.

Cleaved caspase‐3 and Ki67 stainings were assessed on 5‐μm frozen sections of cell‐derived melanoma xenografts. Samples were fixed for 30 min with 4% PFA in PBS, rehydrated for 10 min in Tris 0.1 M, permeabilized in Tris 0.1 M + 1% Triton for 1 h, and then blocked for 1 h in Tris 0.1 M containing 1% BSA, 0.3% Triton, and anti‐CD16/CD32 (FC Block, 1/100). Antibodies were diluted in Tris 0.1 M containing 1% BSA and 0.3% Triton and incubated overnight at 4°C. Following several washes with Tris 0.1 M, bound antibodies were detected using Alexa Fluor 488‐ or Alexa Fluor 594‐conjugated secondary antibody. Images were captured using a wide‐field microscope (Leica DM5500B, at ×40 magnification).

### Mass spectrometry analysis

Proteomic analysis of de‐cellularized 3D ECMs was performed as described (Gopal *et al*, 2017). Briefly, ECM proteins were solubilized in urea, reduced, and alkylated, and proteins were digested with PNGase F (New England BioLabs), endoproteinase Lys‐C (Promega), and high‐sequencing‐grade trypsin. Each sample was reconstituted in 0.1% trifluoroacetic acid (TFA) 2% acetonitrile and analyzed using liquid chromatography (LC)–tandem mass spectrometry (MS/MS) in an LTQ Orbitrap Velos (Thermo Electron, Bremen, Germany) online with a nanoLC Ultimate 3000 chromatography system (Dionex). Protein identification and estimation of abundance were determined using raw LC Orbitrap MS data that were processed using the MASCOT search engine (version 2.4.1). Spectra were searched against a SwissProt Human database. The protein abundance was calculated using the iBAQ score and represented as molar percent.

### Phospho‐kinase profiling

Phospho‐kinase screening was performed using a phospho‐kinase array (Proteome Profiler Human Phospho‐Kinase Array #ARY003B; R&D Systems) according to the manufacturer’s instructions. After cell extraction, 600 μg of protein was added per sample. Array spots were analyzed using ImageJ software.

### Atomic force microscopy

Elastic properties of fibroblast‐derived 3D ECMs were analyzed by AFM using a BioScope Catalyst operating in Point and Shoot (Bruker Nano Surfaces), coupled with an inverted optical microscope (Leica DMI6000B, Leica Microsystems Ltd.). The apparent Young’s modulus (Εapp) was measured on unfixed ECM using a silicon nitride tip (40 nm of diameter) mounted on a cantilever with a nominal spring constant of 0.03 N/m (MLCT‐D, Bruker). The force–distance curves were collected using a velocity of 4.5 μm/s, in relative trigger mode, and by setting the trigger threshold to 1 nN. Each point represents a specific Young's modulus obtained by fitting the corresponding individual force curve acquired on a determined point of the sample. Εapp values were represented as scatter dot plot using GraphPad Prism software.

### Cell line‐derived xenograft (CDX) tumor models

Mouse experiments were carried out in accordance with the Institutional Animal Care and the local ethics committee (CIEPAL‐Azur agreement NCE/2018‐483). 6‐week‐old female athymic nude nu/nu mice were purchased from Janvier Labs (France) and maintained under specific pathogen‐free conditions in our accredited animal housing facility. 1 × 10^6^ 1205Lu melanoma cells were subcutaneously implanted into both flanks. The tumor was measured with a caliper, and the volume was calculated using the formula: V = width × length2 × 0.5. When the tumor reached 75 mm^3^, mice were randomly grouped into control and test groups. Vemurafenib (35 mg/kg) and imatinib mesylate (75 mg/kg) were delivered (alone or in combination) intraperitoneally three times per week. Mice in the control group were treated with vehicle alone. Mice were treated for 30 days and followed for up to 50 days or until tumors reached a predefined volume (1,000 mm^3^). Once the animals were sacrificed, tumors were dissected, weighed, and snap‐frozen in liquid nitrogen in an optimal cutting temperature compound (OCT; Tissue‐Tek) (Gentaur). The sections were processed as described above and analyzed using immunofluorescent microscopy or formalin‐fixed and paraffin‐embedded for picrosirius red staining or SHG analysis.

### Fibrillar collagen imaging

Collagen in de‐cellularized 3D ECMs or in paraffin‐embedded melanoma tissues was stained with picrosirius red using standard protocols. Tumor sections were analyzed using polarized light microscopy as previously described (Rich & Whittaker, 2005). Images were acquired under polarized illumination using a light transmission microscope (Zeiss PALM, at 5× magnification). Fiber thickness was analyzed according to the change in polarization color. Birefringence hue and amount were quantified as a percent of total tissue area using ImageJ software. SHG imaging of paraffin‐embedded melanoma tissues was recorded on a Zeiss 510 NLO microscope (Carl Zeiss Microscopy) with the Mai Tai HP DeepSee (Newport Corporation) and 360‐ to 440‐nm band‐pass filter.

### Tissue microarray analysis

Immunohistochemistry analysis of DDR1 and DDR2 expression was assessed on TMA sections (US Biomax, #ME1004h) using VECTASTAIN Elite ABC Kit (Vector Laboratories) with the DAB reagent (Vector Laboratories) and counterstained with hematoxylin (Fisher Scientific), according to the manufacturer’s instructions. Images were captured using a bright‐field microscope (Nikon, at 20× magnification). Histological scoring of the samples was performed in a blinded fashion.

### Analysis of gene expression from public databases

Publicly available gene expression datasets of human melanoma cell lines were used to analyze *DDR1*, *DDR2*, *MITF*, *AXL,* and *SOX10* levels according to the differentiation status. Cell states were classified into four differentiation states (undifferentiated, neural crest‐like, transitory, and melanocytic) as previously described (Tsoi *et al*, 2018) and using GSE80829 and https://systems.crump.ucla.edu/dediff/. *DDR1*, *DDR2*, *MITF*, and *AXL* expression was analyzed in human melanoma samples within the Mannheim (GSE4843), Philadelphia (GSE4841), and Zurich (GSE4840) cohorts (Widmer *et al*, 2012). Proliferative (MITF^high^ AXL^low^) and invasive (MITF^low^ AXL^high^) melanoma subgroups were defined as previously described (Widmer *et al*, 2012; Rathore *et al*, 2019). Genomic alterations in *DDR1* and *DDR2* from the skin melanoma TCGA database were retrieved using cBioPortal (http://www.cbioportal.org/). Correlation between *DDR1* genomic alterations and expression and the activity (−log_10_[IC50 M]) of BRAFi (dabrafenib, vemurafenib) and MEKi (trametinib) were performed across the GDSC (Genomic of Drug Sensitivity in Cancer) melanoma cell line pharmacogenomic data using CellMinerCDB (https://discover.nci.nih.gov/cellminercdb; Luna *et al*, 2021).

### Statistical analysis

Unless otherwise stated, all experiments were repeated at least three times and representative data/images are shown. Statistical data analysis was performed using GraphPad Prism 8 software. The unpaired two‐tailed Mann–Whitney test was used for statistical comparisons between two groups. The Kruskal–Wallis test with the indicated post‐tests or two‐way ANOVA test with Sidak’s, Dunnett’s, or Tukey’s post‐tests was used to compare three or more groups. A chi‐square test was performed for statistical analysis of tissue microarray stainings. A log‐rank (Mantel–Cox) test was applied to the Kaplan–Meier survival curves. Data represent biological replicates (n) and are depicted as mean values ± SEM as indicated in the figure legends.

## Author contributions

ST‐D and MD designed the study, analyzed and interpreted data, ensured financial support, and wrote the paper with inputs from IB, AP, VP, and CAG. IB performed the majority of the experiments and analyzed the data with the help of ML, AC, AM, SD, MO, CR, VP, AP, and CAG. FL performed the flow cytometry analyses. SP performed the AFM analyses. SA performed the mass spectrometry analyses. CG and TP contributed to reagents and expertise.

## Conflict of interest

T.P. is the co‐founder of Yukin Therapeutics. The remaining authors declare that they have no conflict of interest.

## Supporting information



Appendix

Expanded View Figures PDF

Source Data for Figure 2

Source Data for Figure 3

Source Data for Figure 4

Source Data for Figure 5

Source Data for Figure 8

## Data Availability

The mass spectrometry proteomics data have been deposited to the ProteomeXchange Consortium (http://www.proteomexchange.org) via the PRIDE partner repository with the dataset identifier PXD026645 (http://www.ebi.ac.uk/pride/archive/projects/PXD026645).
