# Supplementary material for: Targeting Discoidin Domain Receptors DDR1 and DDR2 overcomes matrix‐mediated tumor cell adaptation and tolerance to BRAF‐targeted therapy in melanoma
Source: EMBO Mol Med. 2021 Dec 27;14(2):e11814. doi: 10.15252/emmm.201911814 (PMC8819497; doi:10.15252/emmm.201911814)
Supplement: Supplementary file 1 — Appendix [file EMMM-14-e11814-s005.pdf]

Appendix

Appendix Figure S1. Quantitative mass spectrometry analysis of fibroblast-derived ECM.

Appendix Figure S2. Blocking β1 integrin/FAK pathway does not inhibit ECM-mediated resistance to BRAF inhibition.

Appendix Figure S3. Appendix Figure S3. Higher magnifications of IHC images shown in Figure 3.

Appendix Figure S4. Correlation between DDR1 and DDR2 expression and the melanocytic proliferative $MITF^{high}$ and dedifferentiated invasive $MITF^{low}$ cell states of melanoma.

Appendix Figure S5. Correlation between DDR1 expression and the activity of BRAFi and MEKi.

Appendix Figure S6. Impact of BRAF and MEK inhibition on DDR phosphorylation.

Appendix Figure S7. Analysis of the phospho-proteome of BRAFi-treated melanoma cells cultivated on 3D-ECM.

Appendix Table S1. List of antibodies used in this study.

Collagens (molar %)

Gene names	HDF	MAF	FRC2	FRC5
COL1A1	1.52	0.88	3.62	3.26
COL1A2	2.67	1.20	2.90	2.60
COL3A1	0.35	0.63	4.09	2.89
COL4A1	0.15	0.00	4.44	3.86
COL4A2	0.10	0.00	4.70	3.76
COL5A1	2.37	2.61	2.27	2.53
COL5A2	0.98	1.62	3.12	3.88
COL5A3	7.89	2.61	0.05	0.00
COL6A1	2.73	3.13	2.31	1.80
COL6A2	3.00	2.88	2.18	1.77
COL6A3	3.09	3.35	1.95	1.50
COL8A1	0.93	0.87	2.56	2.30
COL12A1	1.96	2.15	2.52	1.95
COL14A1	0.00	4.81	0.07	0.84
COL15A1	3.19	3.08	0.00	0.36
COL16A1	1.24	2.28	2.70	2.30
COL18A1	0.30	0.06	4.94	4.16

ECM Glycoproteins (molar %)

Gene names	HDF	MAF	FRC2	FRC5
FN1	0,57	1,45	3,59	2,92
TGFBI	0,97	3,17	2,54	2,15
EMILIN2	7,07	4,02	0,00	0,00
TNC	1,72	5,17	2,17	1,05
FBN1	2,13	0,33	3,59	3,42
FBN2	6,05	0,10	2,82	1,81
FBLN1	1,19	6,02	0,15	1,51
FBLN2	2,71	3,14	1,38	1,68
FBLN5	0,27	1,80	4,19	2,73
THBS1	1,45	1,61	2,06	1,67
THBS2	0,04	0,00	4,79	4,95
DPT	4,44	5,25	0,39	0,28
CYR61	0,94	0,17	4,35	3,15
EFEMP2	1,26	2,14	3,40	2,30
EFEMP1	0,44	0,00	3,05	3,28
NID1	0,39	0,07	4,41	3,48
NID2	0,26	0,32	4,39	3,38
MFAP2	2,09	0,57	3,84	3,48
IGFBP3	4,78	3,36	0,00	0,00
IGFBP5	1,01	0,00	5,11	4,87
IGFBP7	0,00	0,00	10,37	0,74
LTBP2	2,66	0,59	3,85	3,34
LTBP1	0,26	0,03	5,29	4,17
MXRA5	4,13	6,33	0,00	0,00
TNXB	5,14	1,98	0,04	0,20
ELN	7,78	1,65	0,00	0,00
AEBP1	4,03	3,97	1,56	1,23
LAMB2	2,17	0,55	1,21	0,21
EMILIN1	2,23	2,46	2,52	2,23
THSD4	2,25	0,05	3,68	3,01
ECM1	1,46	3,09	3,48	2,85
LAMA5	0,17	0,00	0,00	0,72
SBSPON	0,00	0,00	0,00	0,00
SPARC	0,03	0,03	7,16	3,26
HMCN1	0,00	11,11	0,00	0,00
PCOLCE	1,95	1,35	5,72	1,07
ABI3BP	0,00	0,18	0,00	0,00
CTGF	0,00	0,00	8,14	2,01
LAMC1	0,27	0,07	3,77	2,40
PXDN	0,28	0,00	5,48	3,35
LAMB4	0,35	0,05	3,88	3,19
SPON2	0,00	0,00	6,42	4,69
SRPX2	0,07	0,00	5,61	4,58
LAMA4	0,13	0,02	4,07	2,21
MFGE8	0,03	0,00	6,96	3,62
TINAGL1	0,01	0,00	3,04	3,26
LAMB1	0,02	0,00	4,20	2,48

Proteoglycans (molar %)

Gene names	HDF	MAF	FRC2	FRC5
BGN	0,95	0,56	3,59	2,96
HSPG2	0,44	1,75	3,99	3,28
DCN	4,09	1,77	2,52	1,95
LUM	5,63	0,81	2,39	1,69
VCAN	0,96	0,31	2,59	3,26
PRELP	10,34	0,77	0,00	0,00
FMOD	10,78	0,34	0,00	0,00

ECM Regulators (molar %)

Gene names	HDF	MAF	FRC2	FRC5
LOX	0,04	0,67	5,29	3,40
LOXL1	1,95	1,89	3,91	3,10
LOXL2	0,01	0,15	5,44	4,06
LOXL3	0,00	0,00	0,00	0,00
LOXL4	0,64	2,13	5,23	2,02
TGM2	1,89	0,61	4,08	2,36
HTRA1	0,97	2,15	1,88	1,49
SERPINH1	0,27	0,17	5,95	3,61
CSTB	1,27	7,01	2,53	0,30
TIMP3	3,48	2,23	0,93	2,91
CD109	3,64	7,47	0,00	0,00
CTSB	0,38	0,85	6,41	3,09
MMP14	0,00	0,00	8,86	2,25
ADAMTSL1	0,00	0,59	6,61	2,93
ADAMTSL4	7,92	0,88	0,33	0,55
SERPINE2	0,82	0,18	3,63	3,17
SERPINE1	0,00	0,00	5,11	3,71
CTSK	0,86	10,25	0,00	0,00
P4HA2	0,00	0,00	8,30	2,56
P4HA1	0,00	0,00	6,87	3,47
PLOD2	0,00	0,00	8,57	1,96
PLOD1	0,00	0,00	7,47	3,45
HTRA3	4,76	6,35	0,00	0,00
F13A1	2,18	0,41	3,38	2,19
TIMP1	0,00	0,00	8,45	2,66
LEPRE1	0,00	0,00	6,78	3,97
SULF1	0,00	0,00	6,57	4,13

ECM-affiliated proteins (molar %)

Gene names	HDF	MAF	FRC2	FRC5
LGALS1	1,00	1,18	5,47	2,91
LGALS8	0,36	1,63	7,84	1,04
LGALS3	0,40	2,82	5,66	2,20
ANXA2;ANXA2P2	1,21	1,07	4,75	2,47
ANXA1	0,66	0,27	5,88	2,83
ANXA6	0,12	0,05	8,59	2,03
ANXA5	0,18	0,22	7,66	2,69
GREM1	0,18	0,49	4,57	2,41

Secreted Factors (molar %)

Gene names	HDF	MAF	FRC2	FRC5
S100A4	9,17	1,94	0,00	0,00
S100A6	3,80	1,10	3,12	2,00
S100A9	1,95	3,39	1,42	1,83
S100A10	1,04	0,45	6,38	3,10
S100A11	2,02	0,21	5,49	2,79
S100A13	1,28	5,46	3,44	0,80
CXCL12	5,80	4,96	0,16	0,14
GDF15	0,15	0,00	4,72	4,51
WNT5A	9,64	0,00	0,97	0,14
FGF2	0,58	0,70	3,63	3,00
ANGPTL4	0,24	0,00	5,21	3,80
ANGPTL2	8,42	2,69	0,00	0,00

Appendix Figure S1. Quantitative mass spectrometry analysis of fibroblast-derived ECM. Complete list of core matrisome and matrisome-associated proteins detected by mass spectrometry of fibroblast-derived ECM produced by HDF, skin MAF or by two lymphatic FRCs (FRC#2 and FRC#5). The table provides the Mascot results and the molar % (iBAQ score) for identified proteins at 1% FDR. Identified proteins were matched with the human matrisome database (Naba A, Ding H, Whittaker CA, Hynes RO. http://matrisomeproject.mit.edu) to retrieve the division and the category. The blue histogram and the values correspond to the molar % calculated for the entire dataset or for the different matrisome categories. Data represent the mean of molar % from 3 independent experiments.

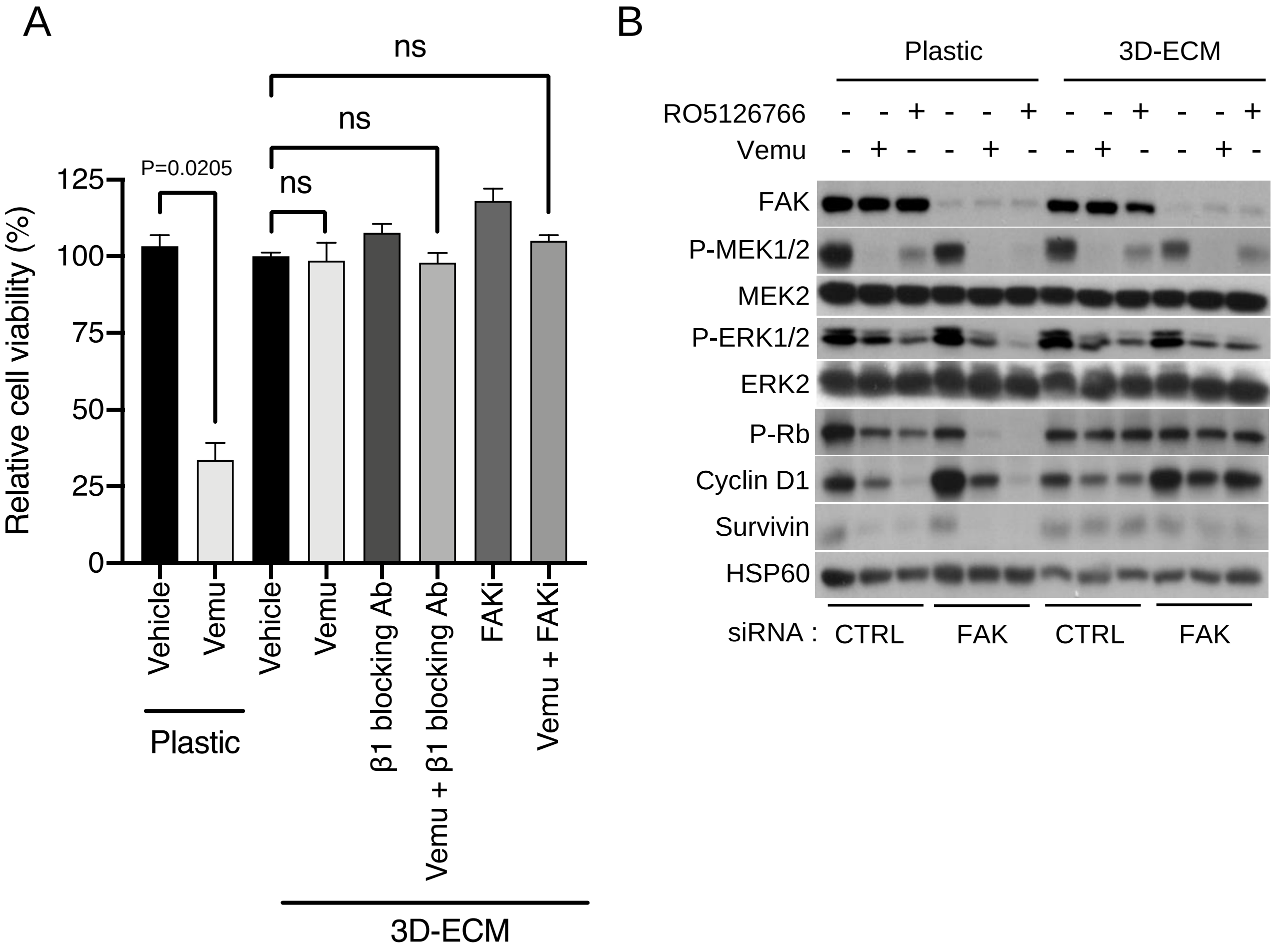

Appendix Figure S2. Blocking β1 integrin/FAK pathway does not inhibit ECM-mediated resistance to BRAF pathway inhibition.

A Quantification of 501MEL melanoma cell proliferation following 48 h of culture on plastic or FRC-derived ECM prior to treatment with 2 µM Vemurafenib in the presence or not of β1 integrin blocking antibodies (10 µg/ml) or FAK inhibitor (5 µM) for 7 h. ns, non significant, *P<0.05, Kruskal-Wallis test.

B Immunoblotting of protein extracts from siCTRL- or siFAK-transfected melanoma cells plated on FRC-derived ECM treated with vehicle or 2 µM BRAFi Vemurafenib or 1 µM dual RAF/MEK inhibitor RO5126766 for 72 h, using antibodies against FAK, P-MEK1/2, P-ERK1/2, P-Rb, Cyclin D1 or survivin. HSP60, loading control.

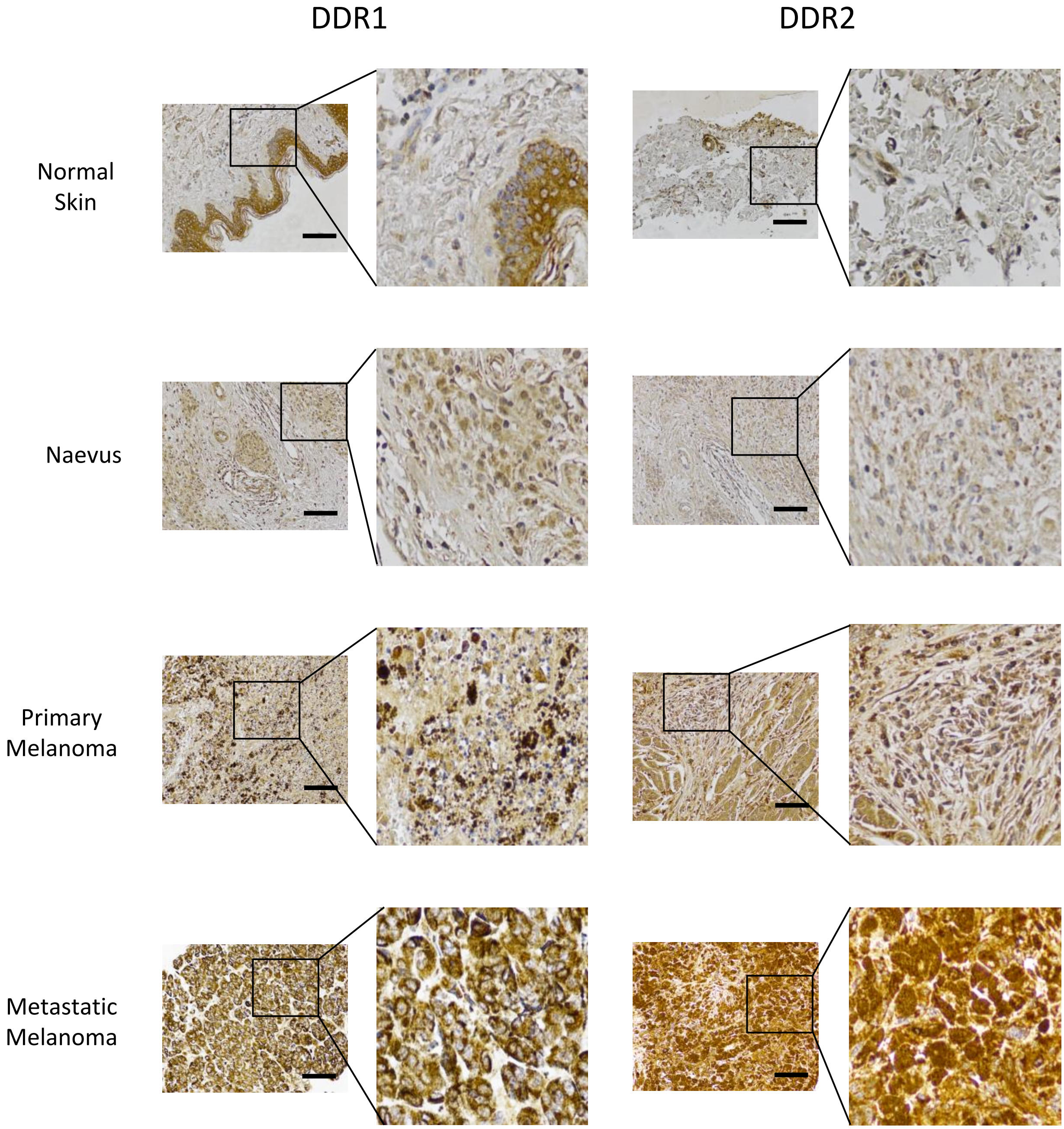

Appendix Figure S3. Higher magnification of IHC images shown in Figure 3.

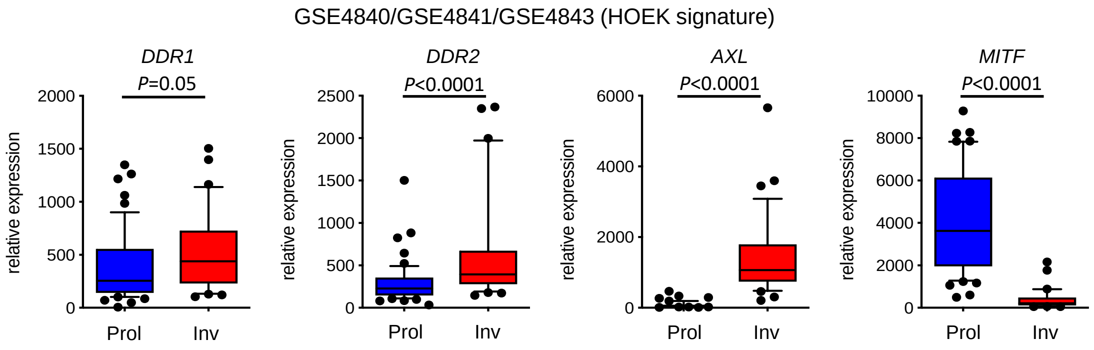

Appendix Figure S4. Correlation between DDR1 and DDR2 expression and the melanocytic proliferative MITF^{high} and dedifferentiated invasive MITF^{low} melanoma cell states.

Box and whisker plots (10th to 90th percentile) show *DDR1* and *DDR2* expression across proliferative (Prol, n=53) *versus* invasive (Inv, n=33) cell states of melanoma cultures within the Mannheim, Philadelphia, and Zurich cohorts (GSE4840, GSE4841 and GSE4843, respectively data sets). The expression of *AXL* and *MITF* is shown as differentiation control markers. n, number of melanoma cell cultures representative of each cell state. p, two-tailed Mann Whitney test.

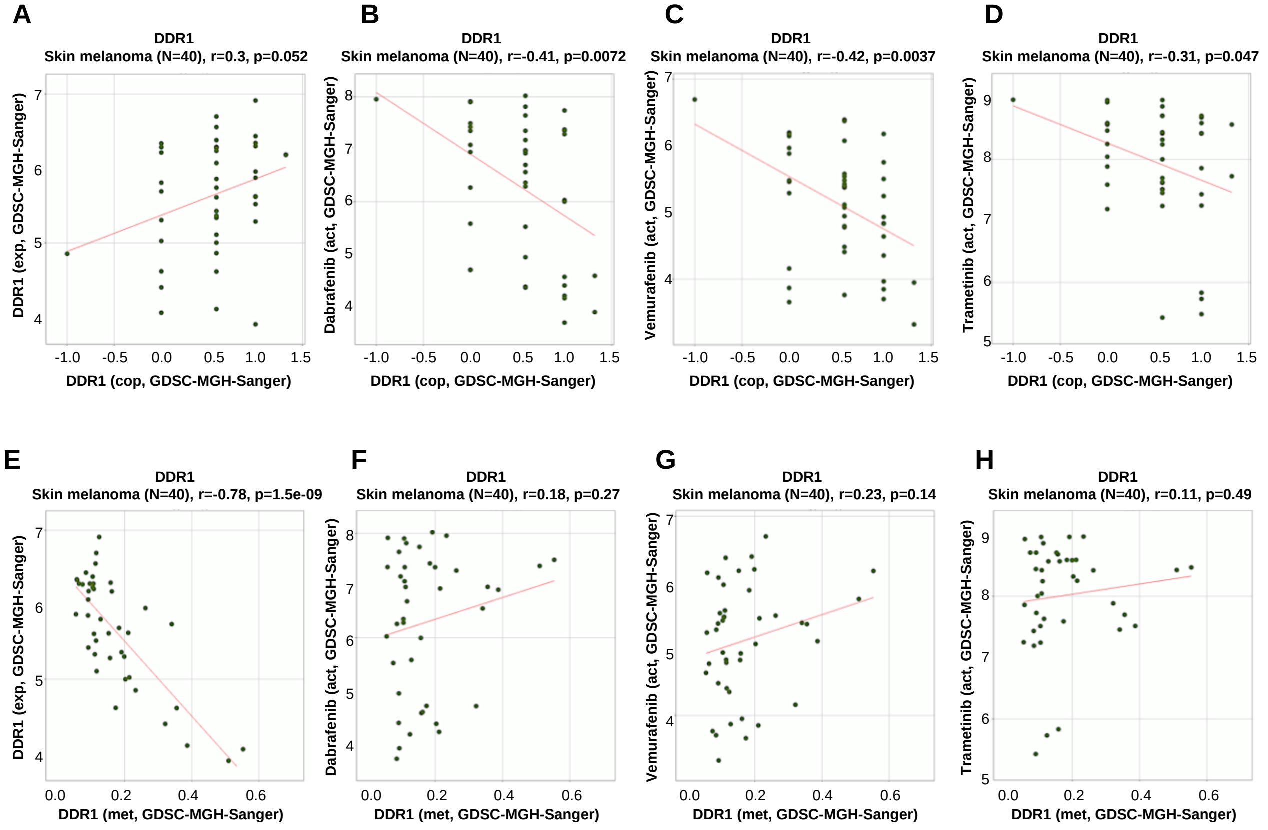

Appendix Figure S5. Correlation between DDR1 expression and the activity of BRAF and MEK inhibitors.

A Analysis of GDSC data (using the webtool https://discover.nci.nih.gov/cellminercdb) indicates that DNA copy number (cop) regulates *DDR1* transcript expression (exp) in melanoma cell lines (n=40).

B-D Activity of BRAFi Dabrafenib or Vemurafenib, and MEKi Trametinib is negatively correlated with *DDR1* DNA copy number in the GDSC. Activity (act), -log10[IC50M].

E Analysis of GDSC data (using the webtool https://discover.nci.nih.gov/cellminercdb) indicates that promoter methylation (met) regulates *DDR1* transcript expression (exp) in melanoma cell lines (n=40).

F-H Activity of BRAFi Dabrafenib or Vemurafenib, and MEKi Trametinib is correlated with *DDR1* promoter methylation. Activity (act), -log10[IC50M].

p values were obtained from Pearson's correlation coefficients (r).

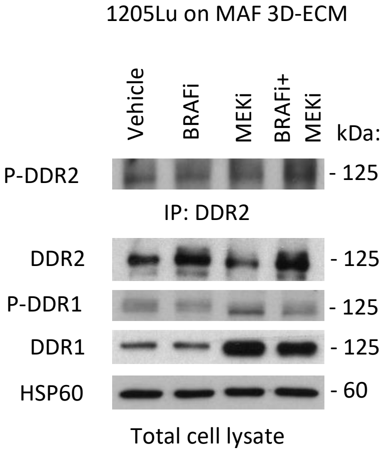

Appendix Figure S6. Impact of BRAF and MEK inhibition on DDR phosphorylation.
1205Lu cells were cultivated on MAF-derived matrices and treated with vehicle, 5 µM BRAFi, 0.01 µM MEKi or 2 µM BRAFi plus 0.01 µM MEKi. Protein extracts were then immunoblotted using antibodies against P-DDR1, DDR1, DDR2. A fraction of the lysates was immunoprecipitated with an antibody against DDR2 and immunoblotted using anti P-DDR1/P-DDR2. HSP60, loading control.

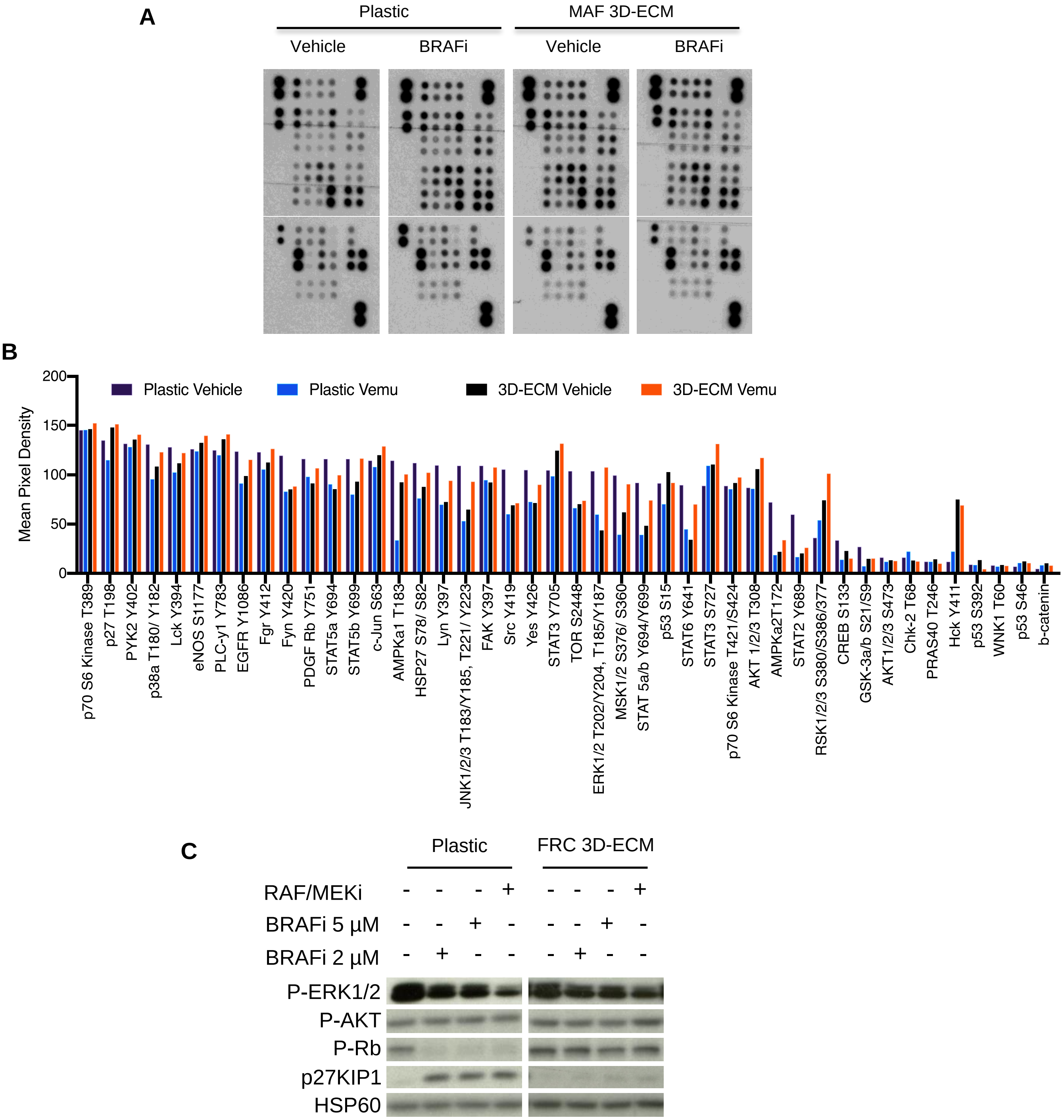

Appendix Figure S7. Analysis of the phospho-proteome of BRAFi-treated melanoma cells cultured on 3D-ECM.

A Kinases and phosphorylated substrates were detected using an immunoblot array (Proteome Profiler Human phospho-kinase array kit). Cells were cultured on plastic or MAF 3D-ECM for 48 h prior treatment with 2 µM of the BRAFi Vemurafenib for additional 2 days before preparation of cell lysates and immunoblot array analysis following manufacturer's instructions.

B Mean pixel density was analyzed using Image J software.

C Protein extracts from 1205Lu cells plated on FRC-derived ECM treated with vehicle, 2 or 5 µM BRAFi Vemurafenib or 1 µM dual RAF/MEK inhibitor RO5126766 for 72 h were immunoblotted using anti P-ERK1/2, P-AKT, P-Rb and p27KIP1. HSP60, loading control.

Appendix Table S1. List of antibodies used in this study

Primary Antibody	Company	Catalog number	Dilutions
AXL (C89E7)	Cell Signaling	8661	WB 1:1000
Caspase 3	Cell Signaling	9662	WB 1:500
Cleaved Caspase 3	Cell Signaling	9661	IF 1:100
Collagen I	Abcam	ab34710	IF 1:500
Cyclin D1 (DCS-6)	BD Biosciences	556470	WB 1:1000
DDR1 (D1G6) XP®	Cell Signaling	5583	WB 1:1000 IHC 1:100
DDR2	Cell Signaling	12133	WB 1:1000
DDR2 (3B11E4)	Santa Cruz	sc-81707	IHC 1:50
E2F1	Cell Signaling	3742	WB 1:1000
ERK2	Santa Cruz	sc-1647	WB 1:1000
FAK	Upstate	05-182	WB 1:1000
Fibronectin (EP5)	Santa Cruz	sc-8422	IF 1:200
HSP60 (K19)	Santa Cruz	sc-1722	WB 1:1000
Ki67	Abcam	ab16667	IF 1:250
MEK2	GeneTex	GTX630542	WB 1:1000
MITF (C5)	Invitrogen	MA5-14146	WB 1:1000
NFkB2 p100/p52 (18D10)	Cell Signaling	3017	WB 1:1000
p53 (DO-1)	Santa Cruz	sc-126	WB 1:1000
p-AKT (Ser473)	Cell Signaling	9271	WB 1:1000
p-DDR1 (Tyr792)	Cell Signaling	11994	WB 1:1000 IF 1:100
p-DDR1 (Tyr796)/p-DDR2 (Tyr740)	R&D System	MAB25382	WB 1:1000 IF 1:100
p-ERK1/2 (Thr202/ Tyr204)	Cell Signaling	9101	WB 1:1000
p-FAK (Tyr397)	Cell Signaling	3283	WB 1:1000
p-MEK1/2 (Ser 221) (166F8)	Cell Signaling	2338	WB 1:1000
p-Rb (Ser795)	Cell Signaling	9301	WB 1:1000
p27Kip1 (D69C12)	Cell Signaling	3686	WB 1:1000
Rb (4H1)	Cell Signaling	9309	WB 1:1000
RelB (C1E4)	Cell Signaling	4922	WB 1:1000
SOX10	Abcam	ab155279	WB 1:1000
Survivin	Cell Signaling	2808	WB 1:1000

Secondary Antibody	Company	Catalog number	Dilutions
Anti-mouse IgG, HRP-linked Antibody	Cell Signaling	7076	WB 1:2000
Anti-rabbit IgG, HRP-linked Antibody	Cell Signaling	7074	WB 1:2000
Goat anti-Rabbit, Alexa Fluor® 488	Invitrogen	A11034	IF 1:200
Goat anti-Rabbit, Alexa Fluor® 594	Invitrogen	A11012	IF 1:200
mouse anti-goat IgG-HRP	Santa Cruz	sc-2354	WB 1:5000
