# Supplementary material for: Targeting Discoidin Domain Receptors DDR1 and DDR2 overcomes matrix‐mediated tumor cell adaptation and tolerance to BRAF‐targeted therapy in melanoma
Source: EMBO Mol Med. 2021 Dec 27;14(2):e11814. doi: 10.15252/emmm.201911814 (PMC8819497; doi:10.15252/emmm.201911814)
Supplement: Supplementary file 2 — Expanded View Figures PDF [file EMMM-14-e11814-s001.pdf]

# Expanded View Figures

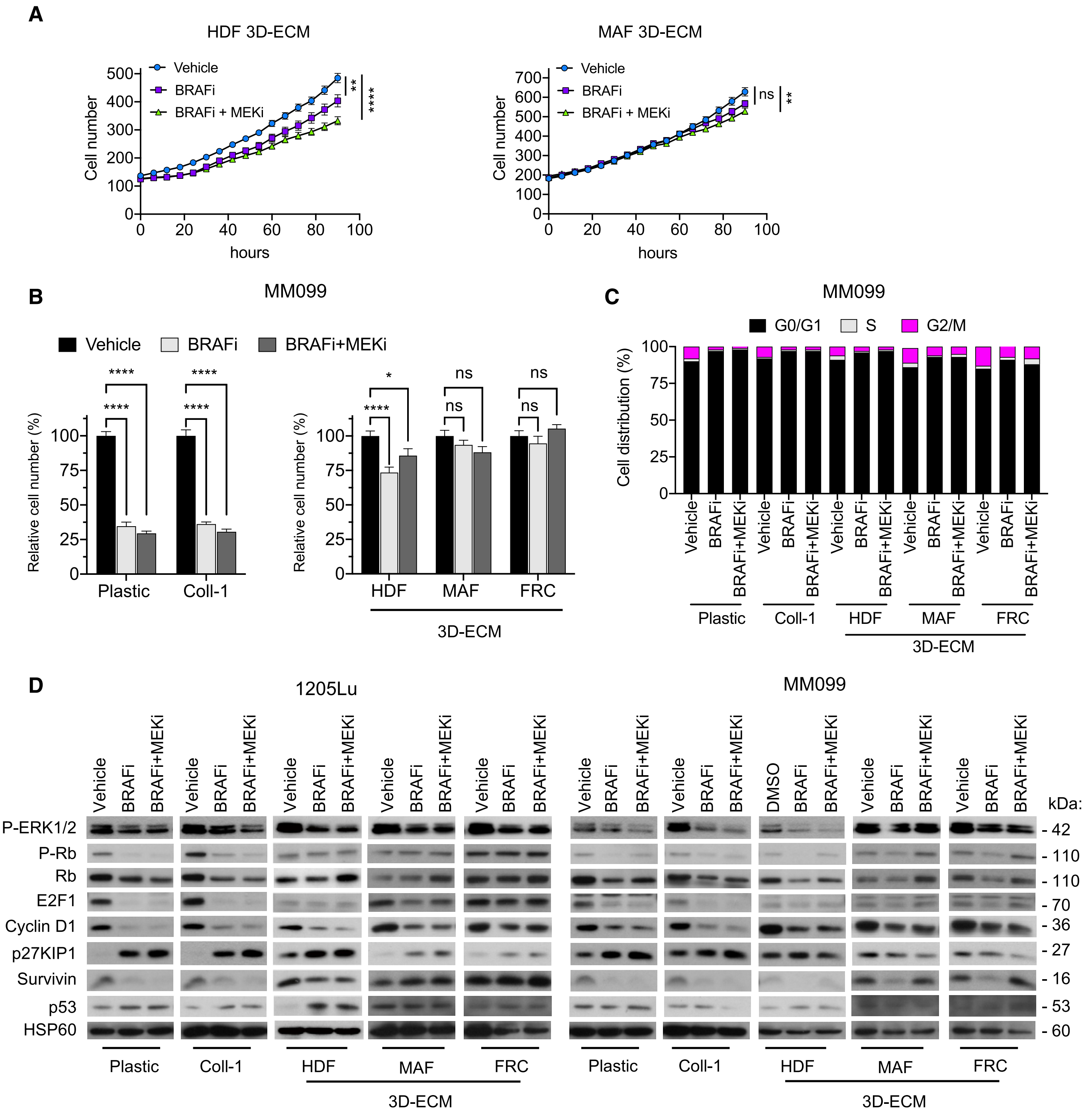


**Figure EV1.**

**Figure EV1. Fibroblast-derived 3D ECM confers drug-protective action to melanoma cells against anti-BRAF$^{V600E}$ therapies.**

A Time-lapse imaging of proliferation of NucLight-labeled 1205Lu cells using the IncuCyte ZOOM system. Cells were plated for 48 h on HDF- or MAF-derived ECMs prior to a 96-h treatment with vehicle, 5 μM BRAFi, or 2 μM BRAFi plus 0.01 μM MEKi. Each data point represents the mean of NucLight red nuclear objects per field ± SEM. ***P* = 0.0079 (left panel), ***P* = 0.0012 (right panel), and *****P* < 0.0001, two-way ANOVA followed by Dunnett's multiple comparisons test. Data are representative of *n* = 3 independent experiments.

B Quantification of proliferation of MM099 short-term melanoma cell cultures plated for 48 h on plastic, Coll-1, or the indicated fibroblast-derived ECMs prior to a 96-h treatment with vehicle, 5 μM BRAFi, or 2 μM BRAFi plus 0.01 μM MEKi. Cells were counted by Hoechst-labeled nucleus staining. Data are represented as bar plots with mean ± SEM normalized to vehicle of 3 independent experiments. **P* = 0.0416 and *****P* < 0.0001, two-way ANOVA followed by Dunnett's multiple comparisons test.

C Flow cytometry analysis of cell cycle distribution of MM099 cells cultured on indicated substrates and treated with vehicle, 5 μM BRAFi, or 2 μM BRAFi plus 0.01 μM MEKi. The percentage of cells in different phases of the cell cycle is indicated.

D Immunoblotting of protein extracts from 1205Lu cells (left panel) and MM099 cells (right panel) cultivated on indicated substrates in the presence or not of 5 μM BRAFi or 2 μM BRAFi plus 0.01 μM MEKi for 96 h, using antibodies against P-ERK1/2, ERK2, P-Rb, Rb, E2F1, survivin, p27KIP1, cyclin D1, and p53. HSP60, loading control.

**Figure EV2. Knockdown and pharmacological inhibition of DDR1 and DDR2 abrogate ECM-mediated resistance to BRAF$^{V600E}$ pathway inhibition.**

A Immunoblotting of protein extracts from 1205Lu and MM099 cells cultivated on HDF- or MAF-derived matrices using antibodies against P-DDR1, P-DDR1/P-DDR2, DDR1, and DDR2. β-actin, loading control.

B Immunoblotting of protein extracts of siCTRL-, siDDR1#2-, siDDR2#2-, or siDDR1#2/siDDR2#2-transfected 1205Lu cells plated on FRC-derived ECM and treated or not with 5 μM BRAFi for 96 h, using antibodies against DDR1, DDR2, P-MEK1/2, P-ERK1/2, ERK2, P-Rb, and survivin. HSP60, loading control.

C Immunoblotting of protein extracts of siCTRL-, siDDR1#1/siDDR2#1, or siDDR1#2/siDDR2#2-transfected MM099 melanoma short-term cultures plated on FRC-derived ECM and treated with vehicle or 2 μM BRAFi combined with 0.01 μM MEKi for 96 h, using antibodies against DDR1, DDR2, P-ERK1/2, ERK2, and cleaved caspase-3. HSP60, loading control.

D Immunoblot analysis of collagen I-induced DDR1 and DDR2 tyrosine phosphorylation. 1205Lu cells were incubated with 10 μg/ml of Coll-I (collagen I) in the presence or not of 7 μM imatinib or 1 μM DDR1-IN-1 for 18 h. After cell lysis, DDR2 phosphorylation was analyzed with anti-P-DDR2 following immunoprecipitation (IP) with anti-DDR2 antibodies. DDR1 phosphorylation was analyzed in total cell lysates with anti-P-DDR1. HSP60, loading control.

E Time-lapse imaging of proliferation of NucLight-labeled 1205Lu cells plated for 48 h on FRC- or MAF-derived ECMs prior to treatment with 5 μM BRAFi in the presence or not of 7 μM imatinib (left panels) or 1 μM DDR1-IN-1 (right panels) for the indicated times. Each data point represents the mean of NucLight red nuclear objects per field ± SEM. One experiment representative of 3 independent experiments is shown. *****P* < 0.0001, 2-way ANOVA followed by Dunnett's multiple comparisons test.

F Quantification of proliferation of SKMEL5 cells plated for 48 h on FRC- (left panel) or MAF-derived ECMs (right panel) prior to a 96-h treatment with vehicle or 5 μM BRAFi in the presence or not of 10 μM imatinib or 5 μM DDR1-IN-1. Cells were counted by Hoechst-labeled nucleus staining. Data are represented as bar plots with mean ± SEM normalized to vehicle. ****P* = 0.0002, the Mann–Whitney test (*n* = 3).

G Quantification of proliferation of MM099 melanoma short-term cultures plated for 48 h on FRC- (left panel) or MAF-derived ECMs (right panel) prior to a 96-h treatment with vehicle or 5 μM BRAFi in the presence or not of 10 μM imatinib or 3 μM DDR1-IN-1. Cells were counted by Hoechst-labeled nucleus staining. Data are represented as bar plots with mean ± SEM normalized to vehicle. ****P* = 0.0002, the Mann–Whitney test (*n* = 3).


© 2021 The Authors

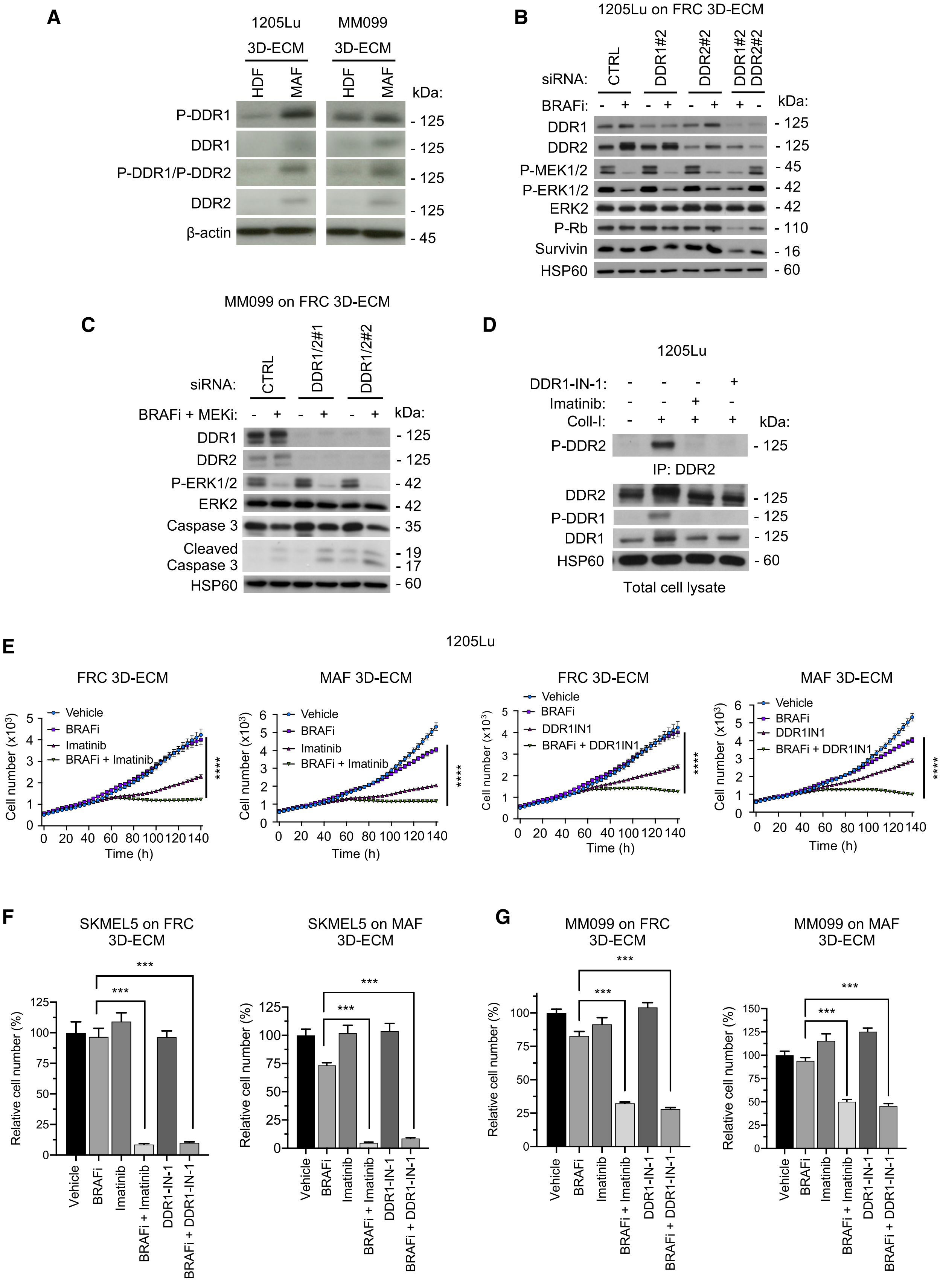
A
1205Lu 3D-ECM
MM099 3D-ECM
HDF MAF HDF MAF
kDa:
P-DDR1 - 125
DDR1 - 125
P-DDR1/P-DDR2 - 125
DDR2 - 125
β-actin - 45
B
1205Lu on FRC 3D-ECM
siRNA: CTRL DDR1#2 DDR2#2 DDR1#2 DDR2#2
BRAFi: - + - + - + + -
kDa:
DDR1 - 125
DDR2 - 125
P-MEK1/2 - 45
P-ERK1/2 - 42
ERK2 - 42
P-Rb - 110
Survivin - 16
HSP60 - 60
C
MM099 on FRC 3D-ECM
siRNA: CTRL DDR1/2#1 DDR1/2#2
BRAFi + MEKi: - + - + - +
kDa:
DDR1 - 125
DDR2 - 125
P-ERK1/2 - 42
ERK2 - 42
Caspase 3 - 35
Cleaved Caspase 3 - 19 - 17
HSP60 - 60
D
1205Lu
DDR1-IN-1: - - - +
Imatinib: - - + -
Coll-I: - + + +
kDa:
P-DDR2 - 125
IP: DDR2
DDR2 - 125
P-DDR1 - 125
DDR1 - 125
HSP60 - 60
Total cell lysate
E
1205Lu
FRC 3D-ECM
MAF 3D-ECM
FRC 3D-ECM
MAF 3D-ECM
Vehicle
BRAFi
Imatinib
BRAFi + Imatinib
DDR1IN1
BRAFi + DDR1IN1
Cell number (x10³)
Time (h)
****
F
SKMEL5 on FRC 3D-ECM
SKMEL5 on MAF 3D-ECM
G
MM099 on FRC 3D-ECM
MM099 on MAF 3D-ECM
Relative cell number (%)
***
Vehicle
BRAFi
Imatinib
BRAFi + Imatinib
DDR1-IN-1
BRAFi + DDR1-IN-1

**Figure EV2.**

**Figure EV3. Pharmacological inhibition of DDR abrogates ECM-mediated resistance to $BRAF^{V600E}$ pathway inhibition and induces cell death.**

A Immunoblotting of protein extracts from 1205Lu cells cultivated on MAF-derived ECM, treated or not with 5 μM BRAFi and/or 7 μM imatinib using antibodies against P-Rb, P-ERK1/2, survivin, or cleaved caspase-3. HSP60, loading control.

B Immunoblotting of protein extracts from SKMEL5 cells (left panel) and MM099 melanoma short-term cultures (right panel) cultivated on MAF-derived ECM, treated or not with 5 μM BRAFi in combination with imatinib (10 μM) or DDR1-IN-1 (5 μM for SKMEL5 and 3 μM for MM099) using antibodies against P-ERK1/2, ERK2, P-Rb, Rb, E2F1, survivin, or cleaved caspase-3. HSP60, loading control.

C Immunoblotting of protein extracts from 1205Lu cells cultivated on FRC-derived ECM for 96 h in the presence of 5 μM BRAFi, 0.01 μM MEKi, or the combination of 2 μM BRAFi and 0.01 μM MEKi, in the presence or not of 10 μM imatinib or 5 μM nilotinib using anti-P-MEK1/2, P-ERK1/2, P-Rb, E2F1, survivin, or cleaved caspase-3 antibodies ($n$ = 2). HSP60, loading control.

D Flow cytometry analysis of cell death (Annexin V/PI labeling) in SKMEL5 cells (left) and MM099 cells (right) plated on FRC-derived ECM and treated by the indicated drugs as described above. Data show the percentage of the different forms of cell death based on Annexin V/PI positivity.


© 2021 The Authors

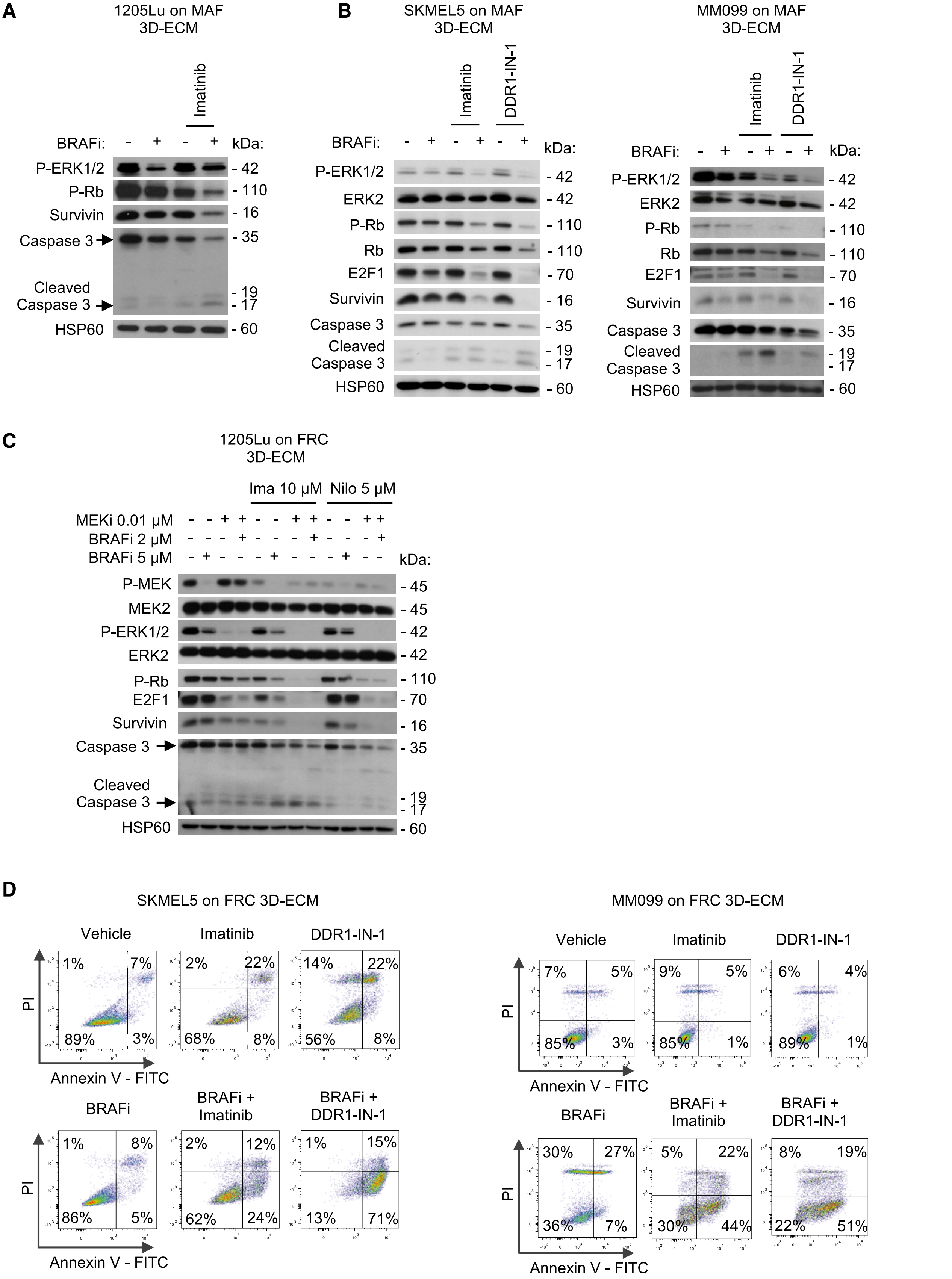
A
1205Lu on MAF 3D-ECM
Imatinib
BRAFi:
kDa:
P-ERK1/2
P-Rb
Survivin
Caspase 3
Cleaved Caspase 3
HSP60
B
SKMEL5 on MAF 3D-ECM
MM099 on MAF 3D-ECM
Imatinib
DDR1-IN-1
ERK2
Rb
E2F1
C
1205Lu on FRC 3D-ECM
Ima 10 µM
Nilo 5 µM
MEKi 0.01 µM
BRAFi 2 µM
BRAFi 5 µM
P-MEK
MEK2
D
SKMEL5 on FRC 3D-ECM
MM099 on FRC 3D-ECM
Vehicle
BRAFi
BRAFi + Imatinib
BRAFi + DDR1-IN-1
PI
Annexin V - FITC

**Figure EV3.**

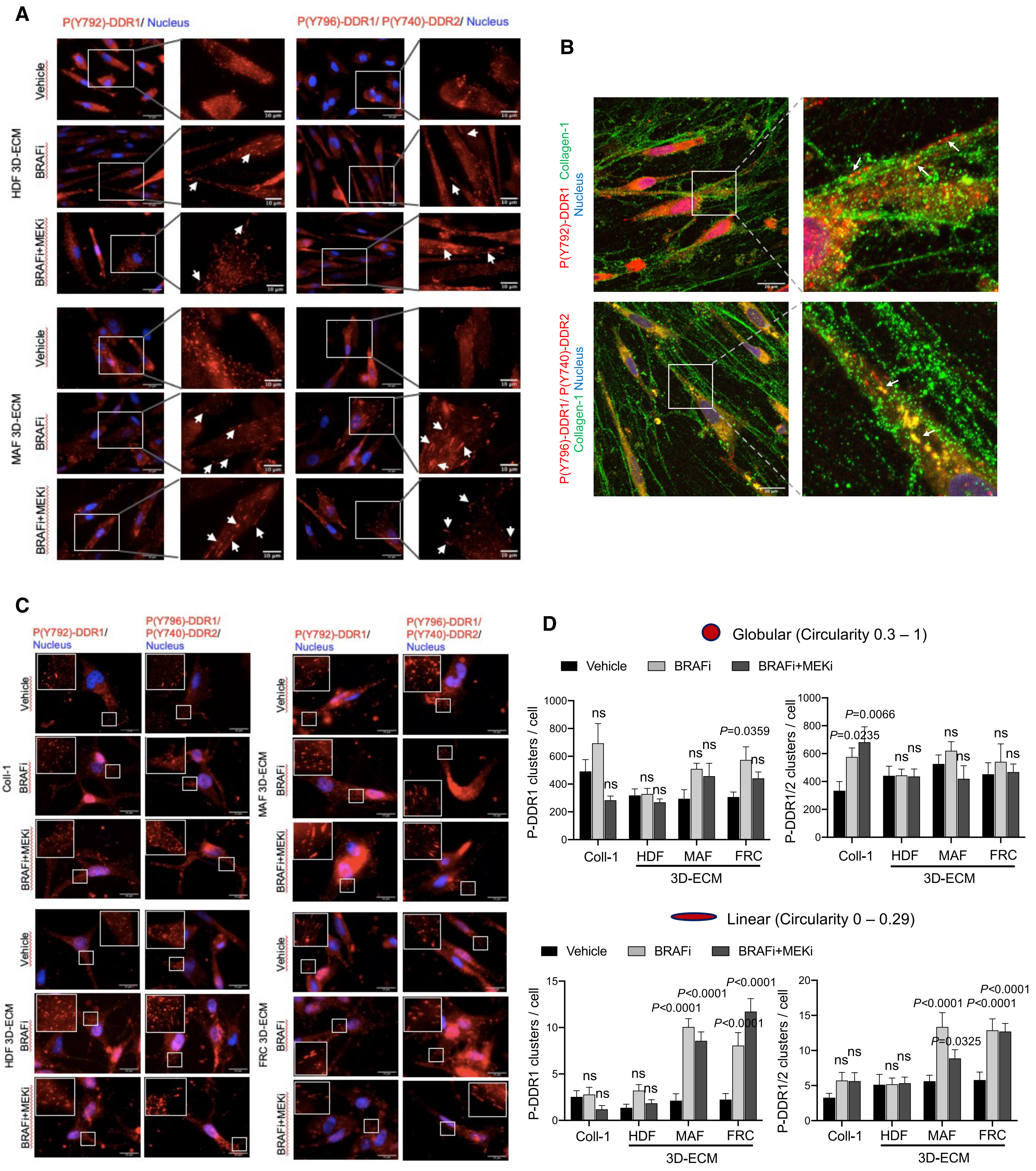


**Figure EV4.**

 © 2021 The Authors

**Figure EV4. Interaction of melanoma cells with 3D ECM induces the clustering of phosphorylated DDR upon BRAFi or BRAFi/MEKi treatment.**

A Representative images of 1205Lu cells cultivated on HDF- or MAF-derived ECM for 48 h prior to treatment with vehicle, 5 μM BRAFi, or 2 μM BRAFi combined with 0.01 μM MEKi for 96 h. Immunofluorescence for phospho-DDR1 (P(Y792)-DDR1) (red; left panels) or phospho-DDR1/2 (P(Y796)-DDR1/P(Y740)-DDR2) (red; right panels) is shown. Nuclei (blue) were stained with DAPI. Enlarged images of P-DDR1 and P-DDR1/2 immunostaining are shown. White arrows indicate P-DDR1 and P-DDR1/2 cell membrane linear clustering. Scale bar = 25 μm (enlarged images, scale bar = 10 μm).

B Analysis of co-localization of phospho-DDR with collagen 1 in 1205Lu cells cultivated on MAF-derived ECM and treated with BRAFi/MEKi. Immunofluorescence for phospho-DDR1 (P(Y792)-DDR1) (red; upper panels) and phospho-DDR1/2 (P(Y796)-DDR1/P(Y740)-DDR2) (red; lower panels), collagen 1 (green), and nuclei (blue) is shown. Enlarged images are shown. White arrows indicate co-localization (yellow fluorescence). Images were captured on Nikon Eclipse Ti confocal microscope at 60x magnification. Scale bar, 20 μm.

C Representative images of SKMEL5 cells cultivated on collagen I (Coll-I) or on indicated fibroblast-derived ECMs for 48 h prior to treatment with vehicle or 5 μM BRAFi or 2 μM BRAFi combined with 0.01 μM MEKi for 96 h. Immunofluorescence for phospho-DDR1 (P(Y792)-DDR1) (red; left panels) or phospho-DDR1/2 (P(Y796)-DDR1/P(Y740)-DDR2) (red; right panels) is shown. Nuclei (blue) were stained with DAPI. Enlarged images of P-DDR1 and P-DDR1/2 immunostaining are shown. Scale bar = 25 μm (enlarged images, scale bar = 10 μm).

D Quantification of globular (left panels) and linear (right panels) clusters of phospho-DDR1 and phospho-DDR1/2 from immunofluorescence staining shown in (B) using ImageJ software. Prior to the quantification of DDR clusters, a "subtract background" function of ImageJ has been applied to all images. In order to quantify clusters, the IsoData threshold has been used. Clusters with circularity 0.3–1 have been defined as "globular", and clusters with circularity 0–0.29 have been defined as "linear". Data are from > 20 individual cells. Error bars reflect mean ± s.d. Values for each treated condition are compared to the vehicle control. 2-way ANOVA followed by Dunnett's multiple comparisons test.

**Figure EV5. DDR and NF-κB2 pathway targeting overcomes MMDR in response to BRAF$^{V600E}$ inhibition.**

A Immunoblot analysis of protein extracts from siCTRL- or siDDR1/2#2-transfected 1205Lu cells plated on MAF-derived ECM in the presence or not of 5 μM BRAFi or 2 μM BRAFi and 0.01 μM MEKi for 96 h using antibodies against DDR1, DDR2, P-ERK1/2, ERK2, RelB, and p100/p52. HSP60, loading control ($n$ = 2).

B Immunoblotting of protein extracts from 1205Lu cells cultivated on MAF-derived ECM treated with vehicle or 5 μM BRAFi and/or 7 μM imatinib, for the indicated time using antibodies against P-ERK1/2, P-Rb, Rb, survivin, caspase-3, cleaved caspase-3, and RelB. HSP60, loading control.

C Immunoblot analysis of protein extracts from MM099 melanoma short-term cultures cultivated on FRC-derived ECM for 96 h in the presence of BRAFi and/or a pan-IKK inhibitor (IKKi, BMS-345541 3 μM) using antibodies against P-ERK1/2, P-Rb, survivin, RelB and HSP60, loading control.

D Immunoblot analysis of protein extracts from 1205Lu (left panel) or SKMEL5 (right panel) cells cultivated on MAF-derived ECM for 96 h in the presence of BRAFi and/or a pan-IKK inhibitor (IKKi, BMS-345541 3 μM) using antibodies against P-ERK1/2, P-Rb, survivin, RelB, and HSP60, loading control.

E Immunoblotting of protein extracts obtained from 1205Lu cells that were plated on FRC-derived (left panel) and MAF-derived (right panel) ECM and treated with 5 μM BRAFi in combination or not with 1 μM DDR inhibitor (DDR1-IN-1) or 10 μM NIK inhibitor (NIKi) for 96 h. Antibodies against P-ERK1/2, P-Rb, Rb, survivin, cleaved caspase-3, RelB, p100/p52, and HSP60 as loading control were used.

© 2021 The Authors

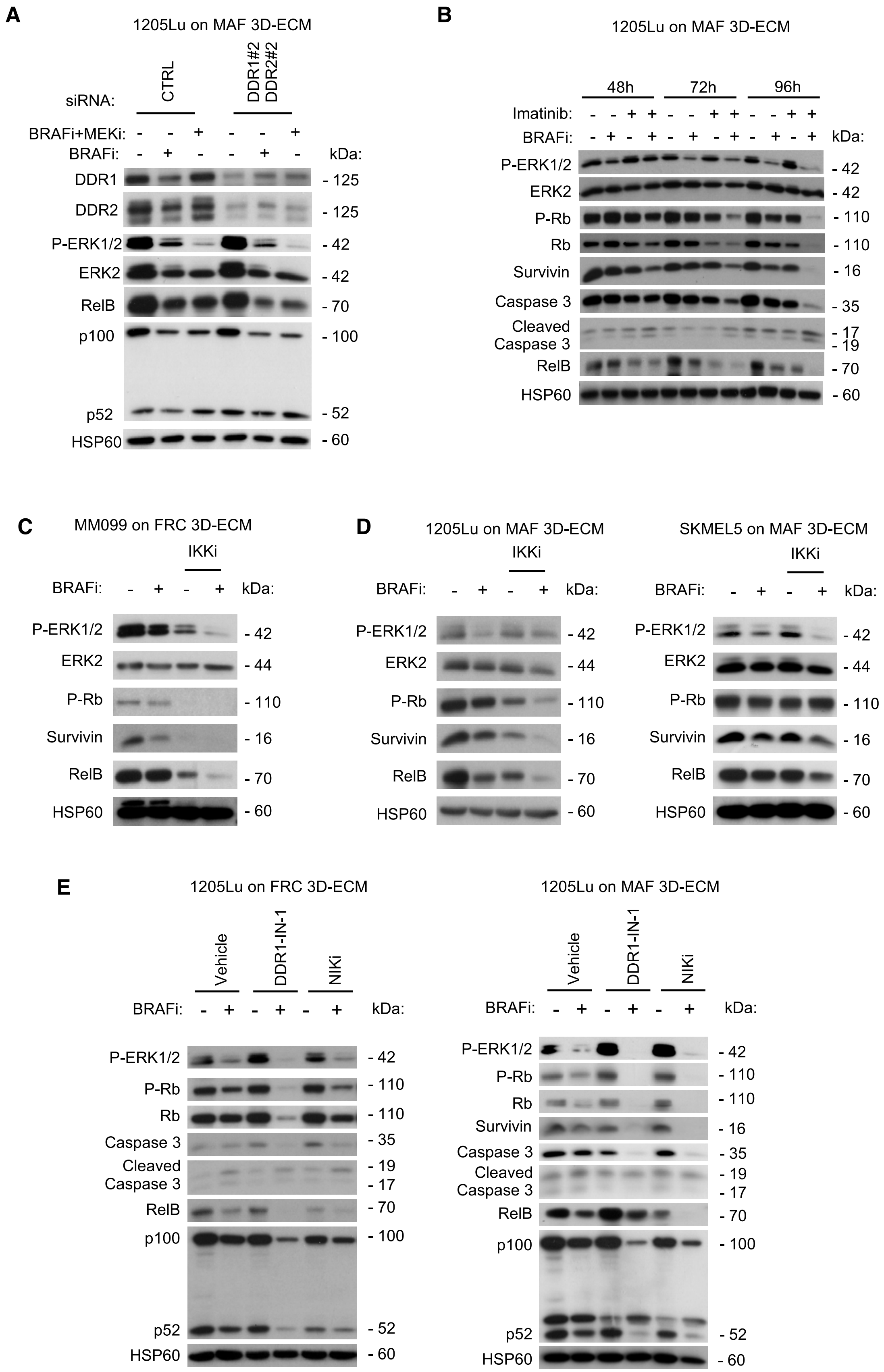


Figure EV5.



© 2021 The Authors
