# Supplementary material for: Targeting Discoidin Domain Receptors DDR1 and DDR2 overcomes matrix‐mediated tumor cell adaptation and tolerance to BRAF‐targeted therapy in melanoma
Source: EMBO Mol Med. 2021 Dec 27;14(2):e11814. doi: 10.15252/emmm.201911814 (PMC8819497; doi:10.15252/emmm.201911814)
Supplement: Supplementary file 3 — Source Data for Figure 2 [file EMMM-14-e11814-s002.zip › Source_data_Figure_2/Source_data_Fig_2G.pptx]

## Slide 1
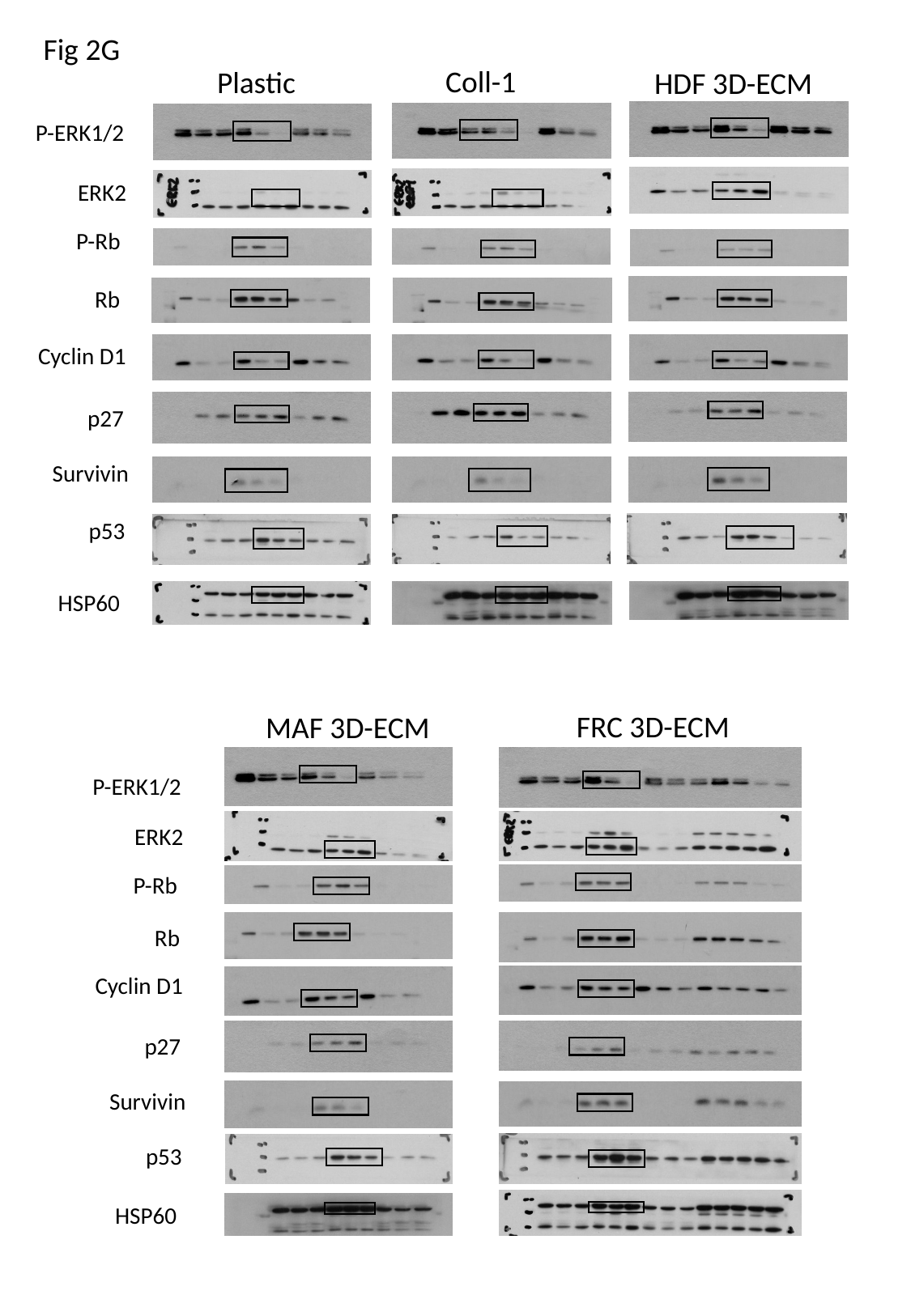

Fig 2G
Coll-1
Plastic
HDF 3D-ECM
P-ERK1/2
ERK2
P-Rb
Rb
Cyclin D1
p27
Survivin
p53
HSP60
FRC 3D-ECM
MAF 3D-ECM
P-ERK1/2
ERK2
P-Rb
Rb
Cyclin D1
p27
Survivin
p53
HSP60
