# Supplementary figures and images for: Targeting Discoidin Domain Receptors DDR1 and DDR2 overcomes matrix‐mediated tumor cell adaptation and tolerance to BRAF‐targeted therapy in melanoma
Source: EMBO Mol Med. 2021 Dec 27;14(2):e11814. doi: 10.15252/emmm.201911814 (PMC8819497; doi:10.15252/emmm.201911814)

## Slide 1
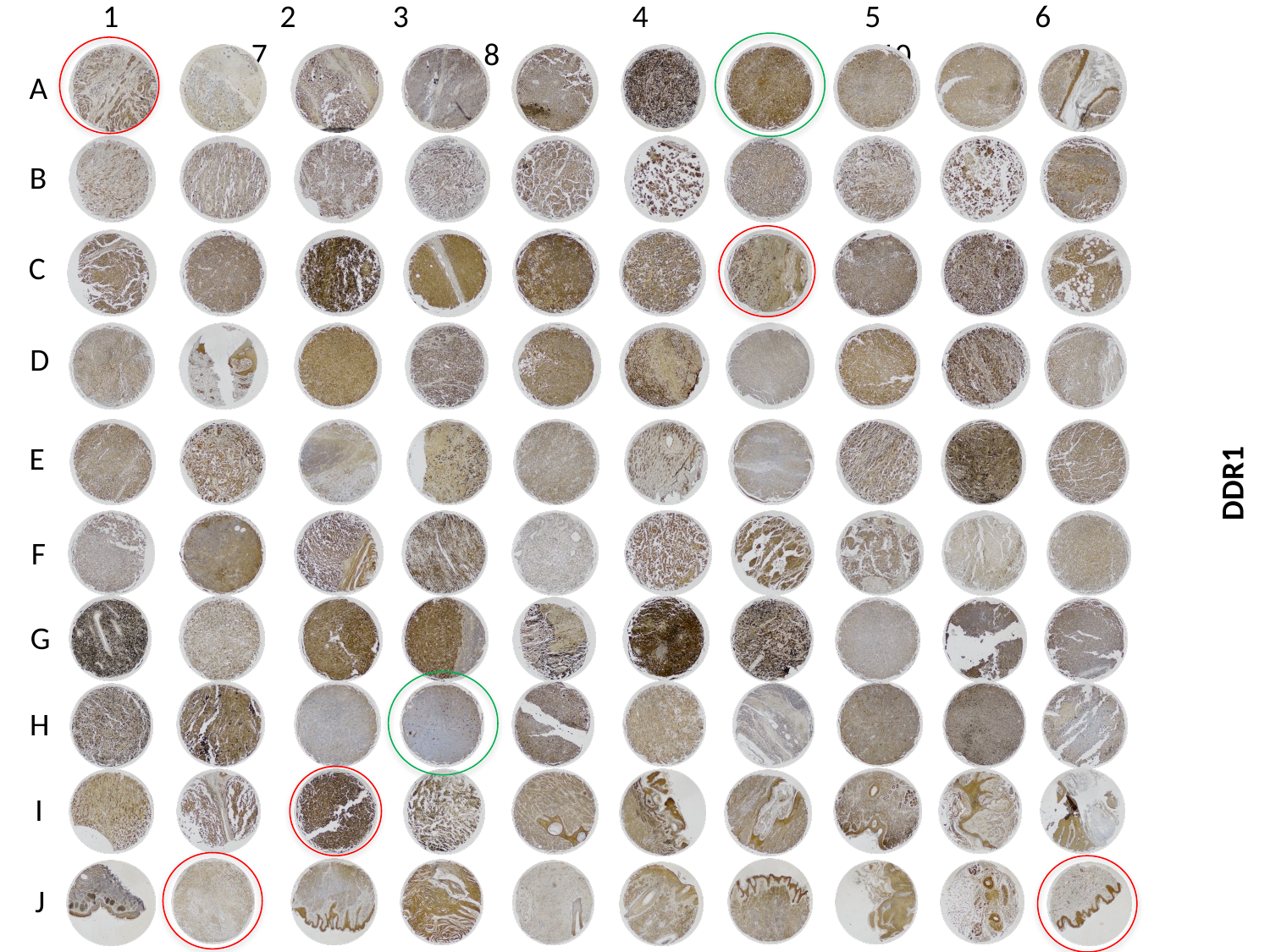

1	 2	 3		 4		5	 6		 7		8	 9		 10
A
B
C
D
E
DDR1
F
G
H
I
J

## Slide 2
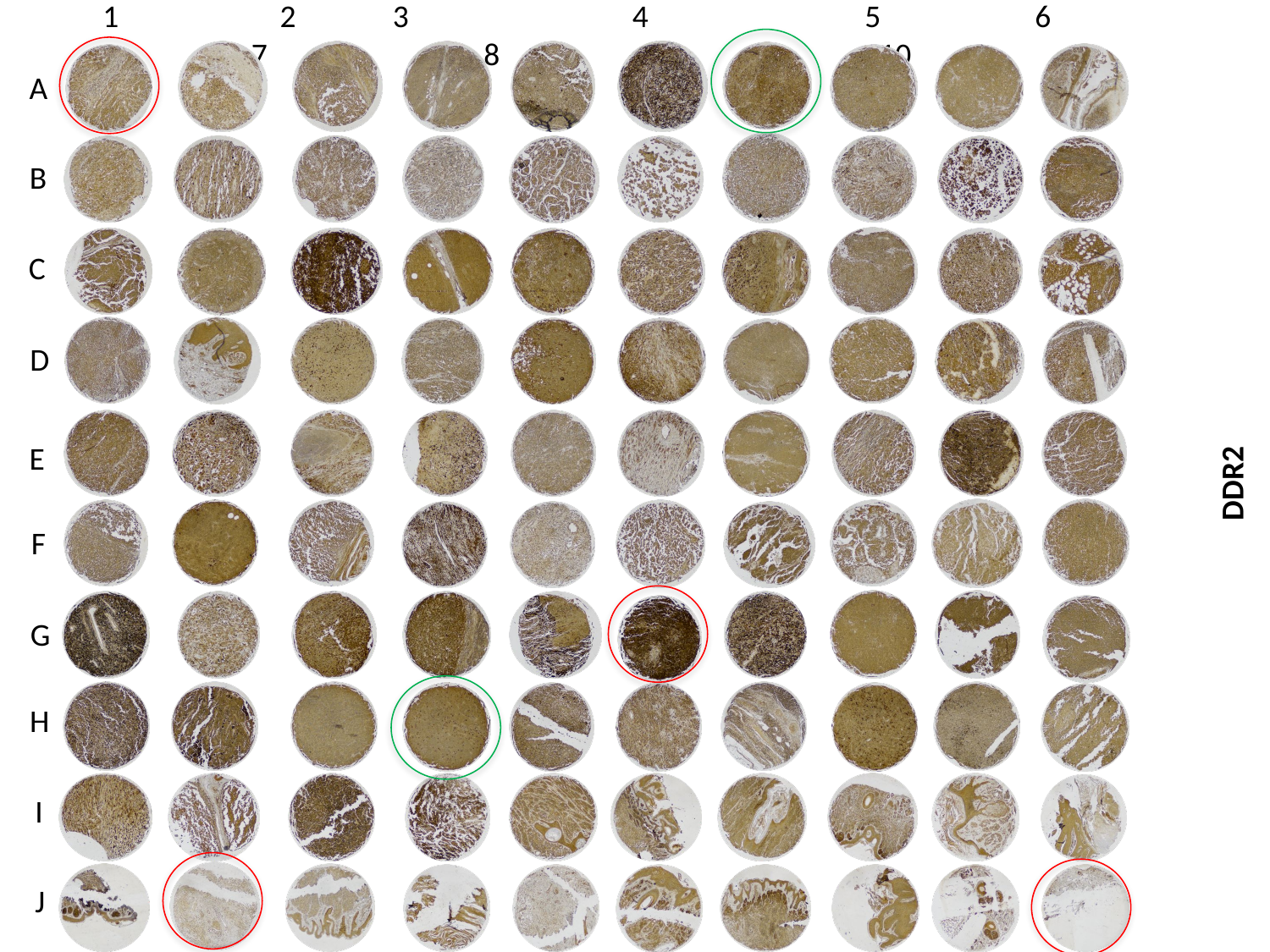

1	 2	 3		 4		5	 6		 7		8	 9		 10
A
B
C
D
E
DDR2
F
G
H
I
J

## Slide 3
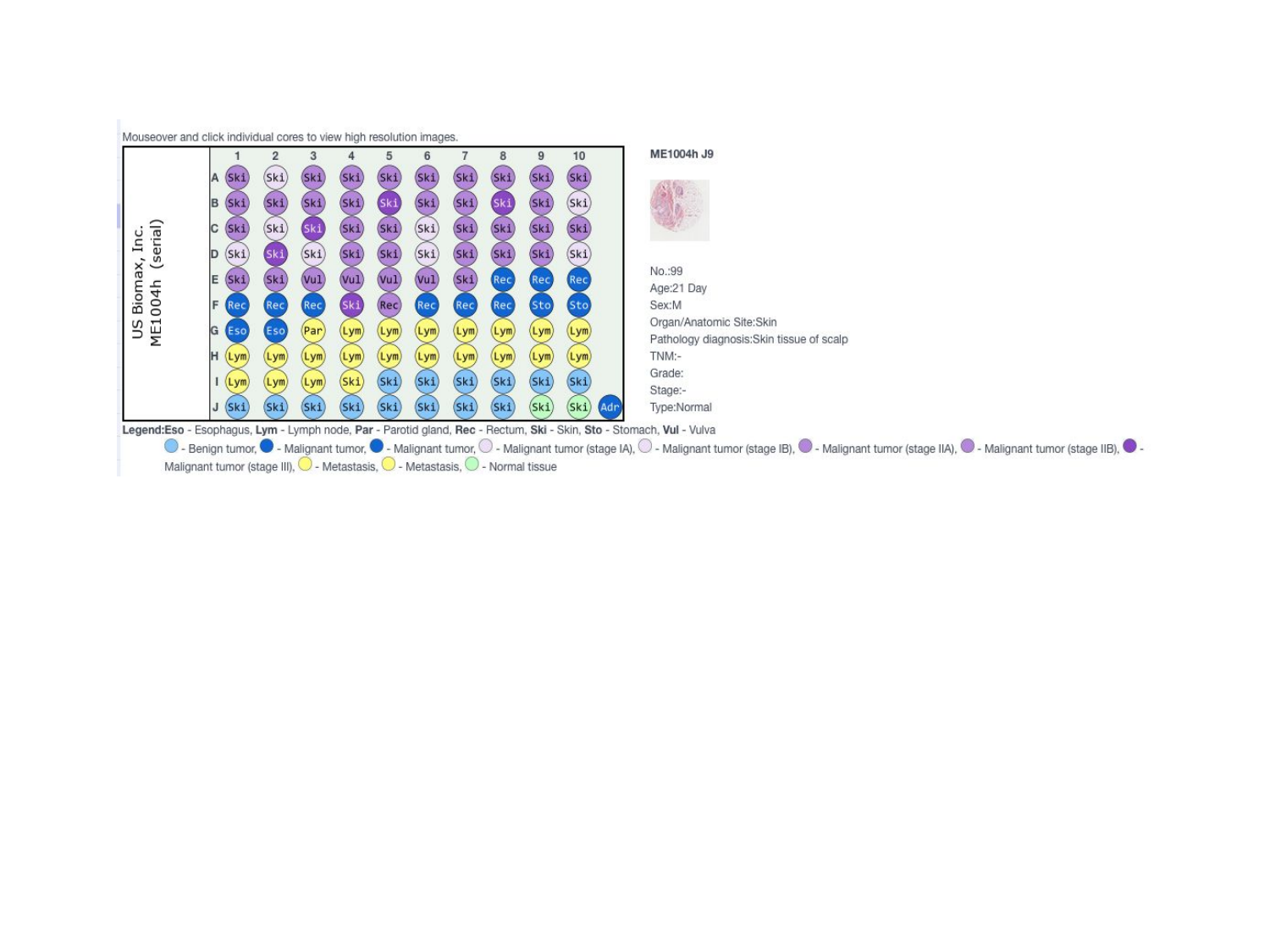

Supplement: Supplementary file 4 — Source Data for Figure 3 [file EMMM-14-e11814-s007.zip › Source_data_Figure_3/Source_data_Fig_3B.pptx]

## Slide 1
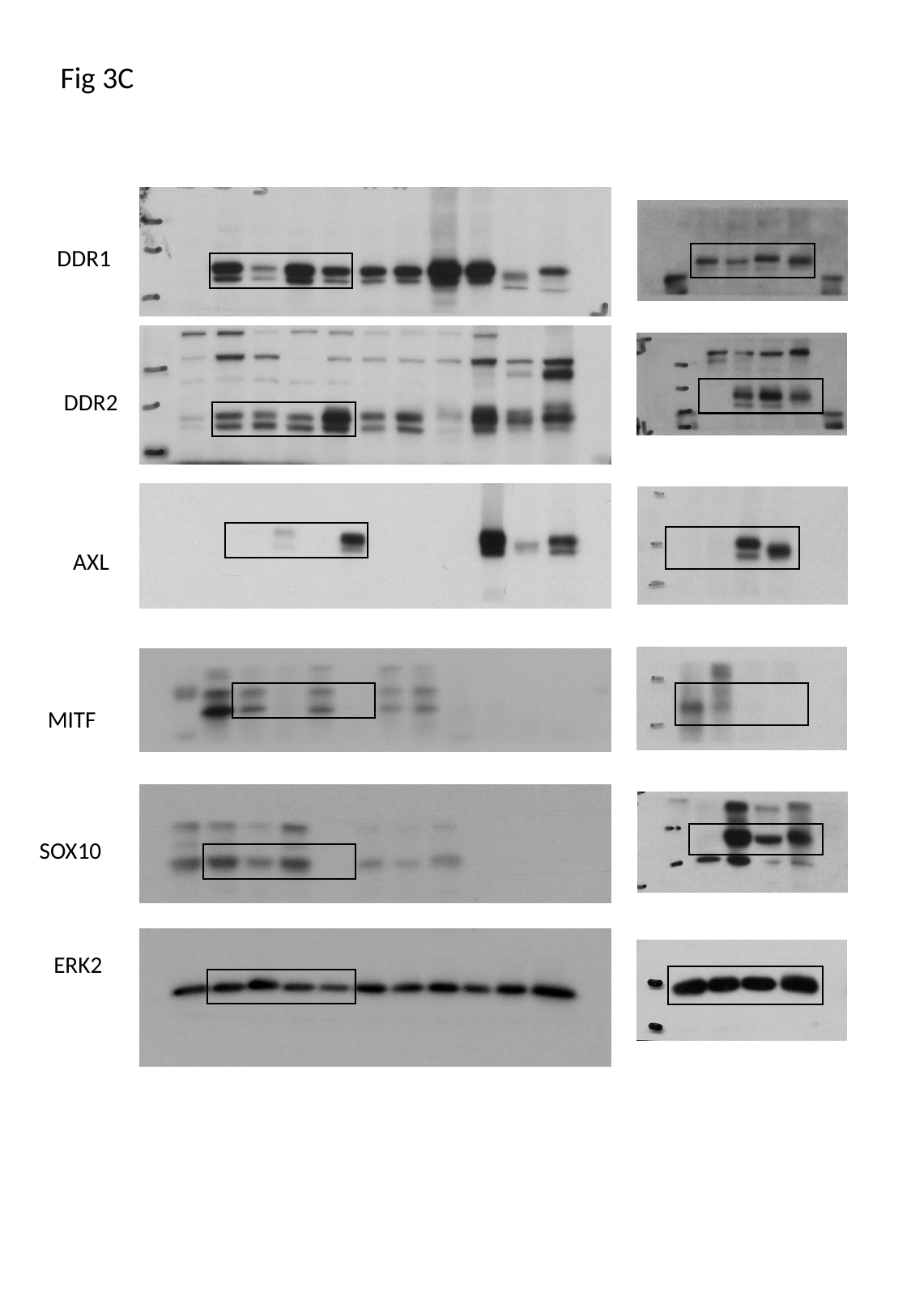

Fig 3C
DDR1
DDR2
AXL
MITF
SOX10
ERK2

Supplement: Supplementary file 4 — Source Data for Figure 3 [file EMMM-14-e11814-s007.zip › Source_data_Figure_3/Source_data_Fig_3C.pptx]

## Slide 1
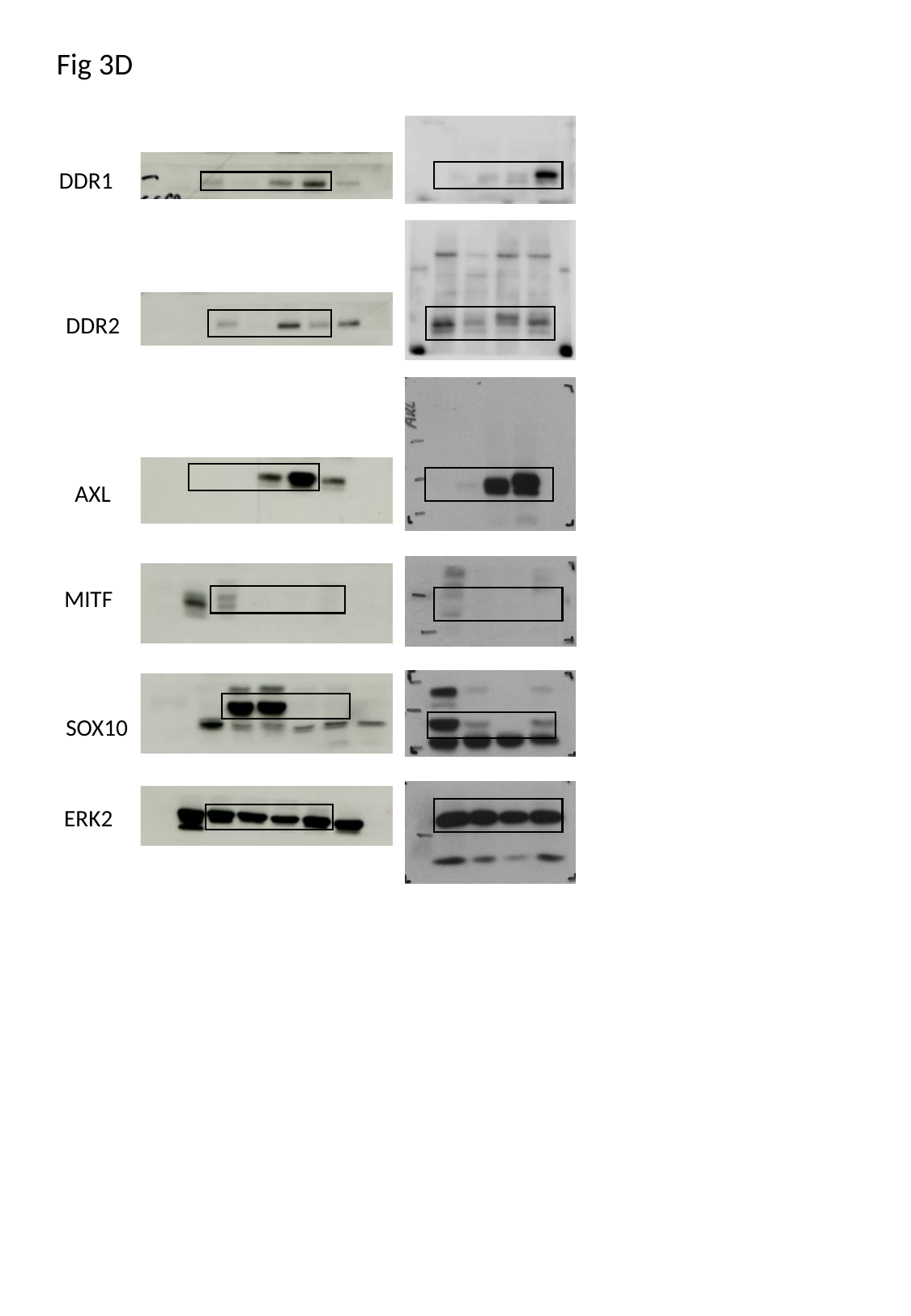

Fig 3D
DDR1
DDR2
AXL
MITF
SOX10
ERK2

Supplement: Supplementary file 4 — Source Data for Figure 3 [file EMMM-14-e11814-s007.zip › Source_data_Figure_3/Source_data_Fig_3D.pptx]

## Slide 1
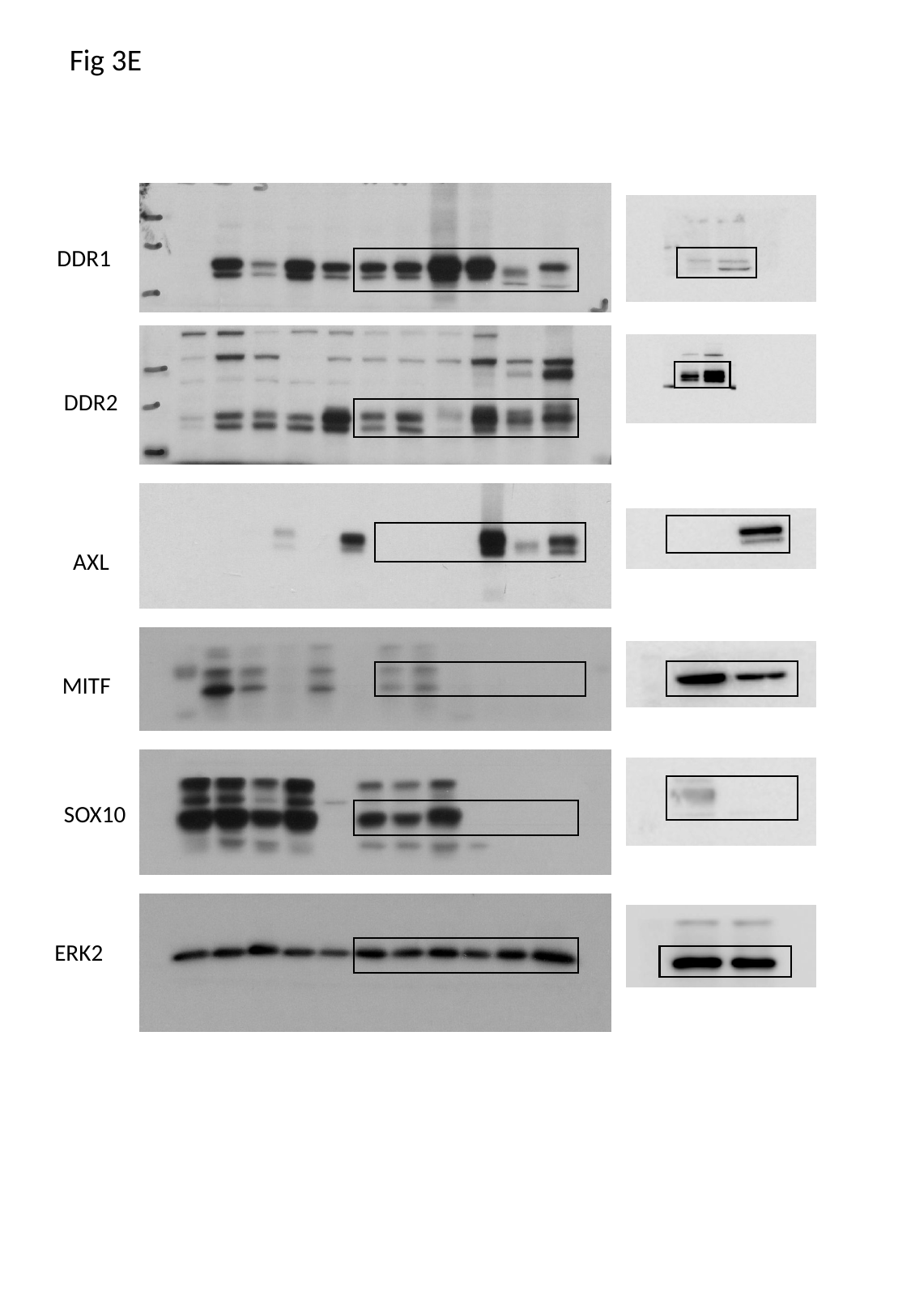

Fig 3E
DDR1
DDR2
AXL
MITF
SOX10
ERK2

Supplement: Supplementary file 4 — Source Data for Figure 3 [file EMMM-14-e11814-s007.zip › Source_data_Figure_3/Source_data_Fig_3E.pptx]

## Slide 1
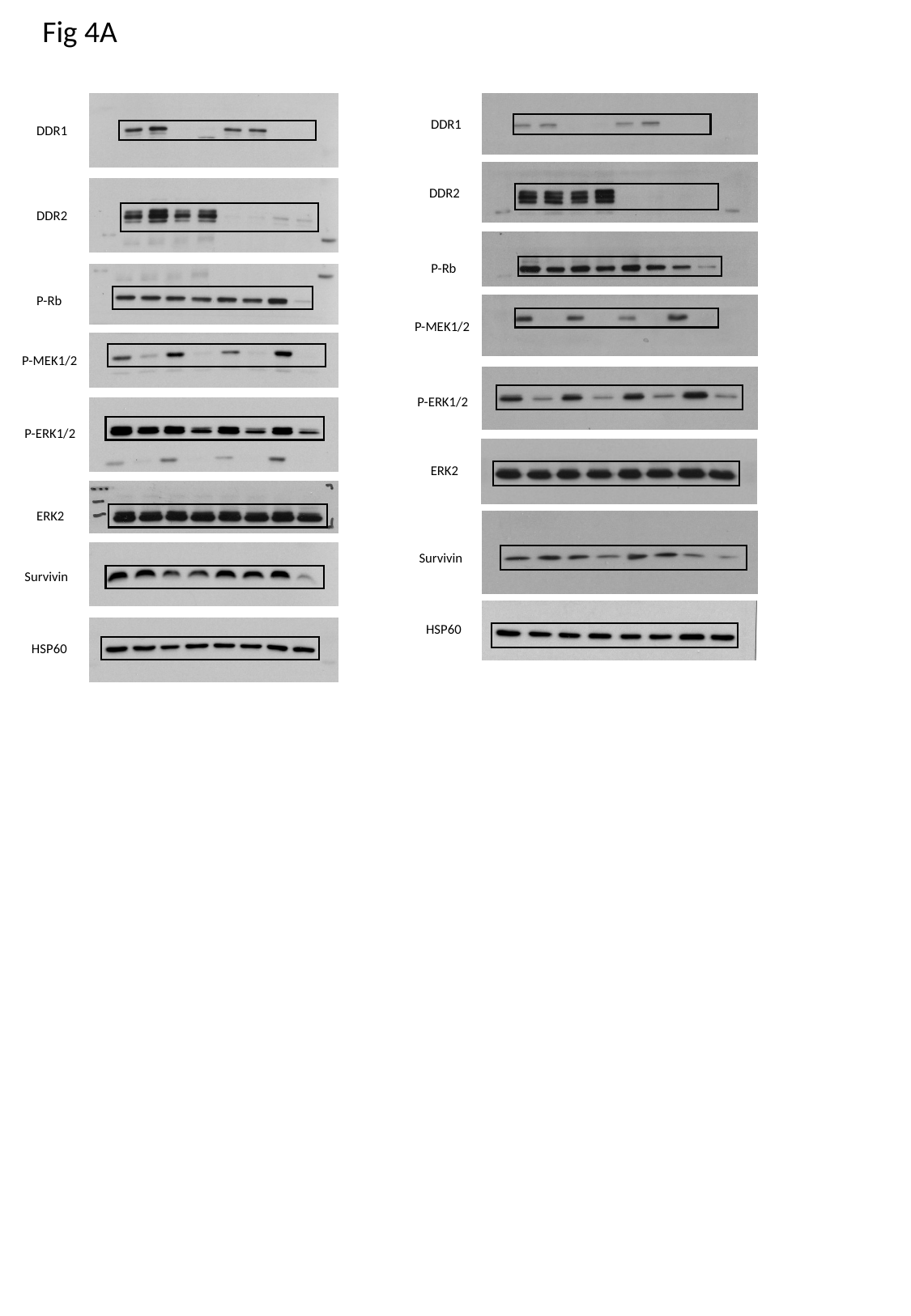

Fig 4A
DDR1
DDR2
P-Rb
P-MEK1/2
P-ERK1/2
ERK2
Survivin
HSP60
DDR1
DDR2
P-Rb
P-MEK1/2
P-ERK1/2
ERK2
Survivin
HSP60

Supplement: Supplementary file 5 — Source Data for Figure 4 [file EMMM-14-e11814-s006.zip › Source_data_Figure_4/Source_data_Fig_4A.pptx]

## Slide 1
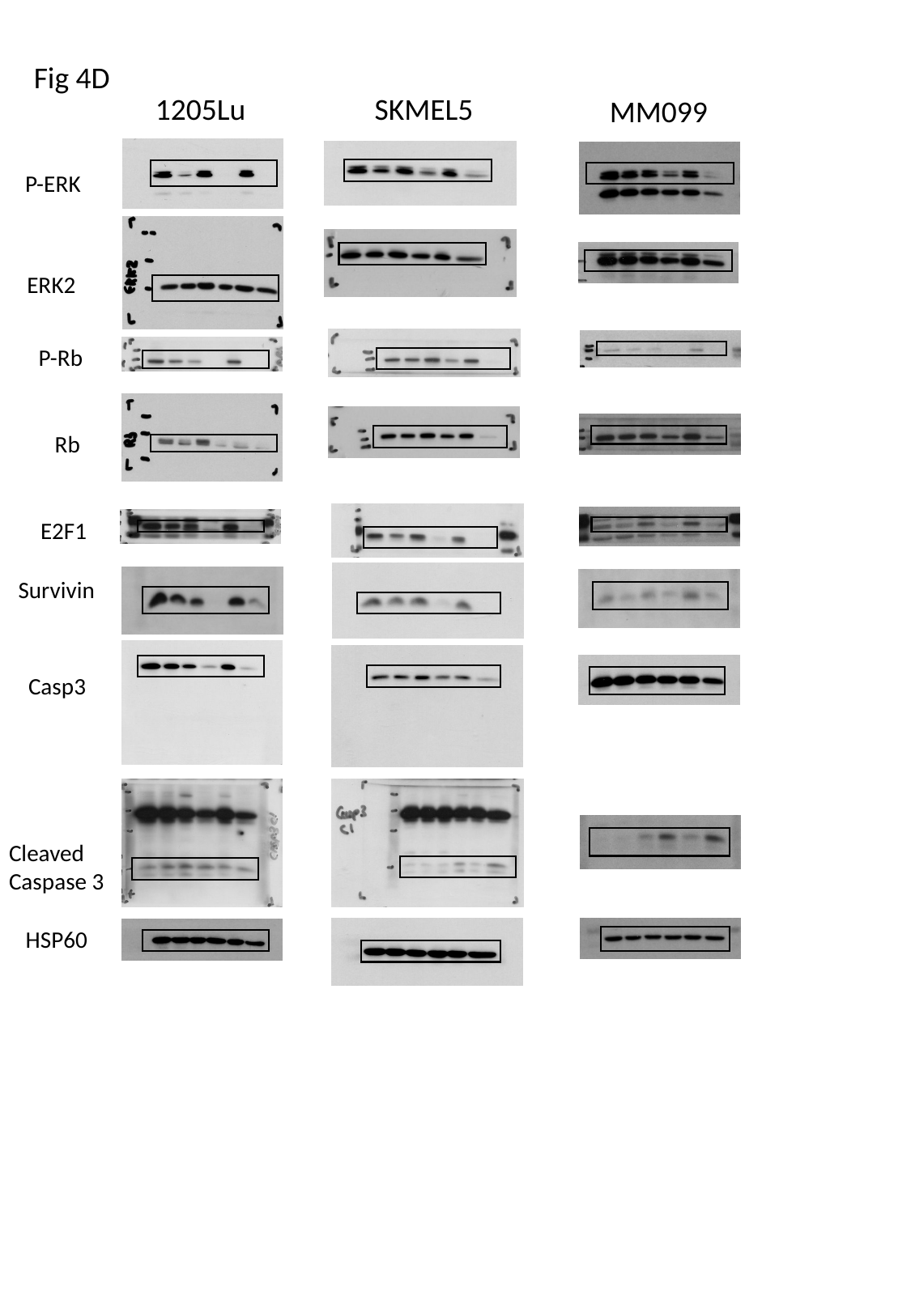

Fig 4D
1205Lu
SKMEL5
MM099
P-ERK
ERK2
P-Rb
Rb
E2F1
Survivin
Casp3
Cleaved
Caspase 3
HSP60

Supplement: Supplementary file 5 — Source Data for Figure 4 [file EMMM-14-e11814-s006.zip › Source_data_Figure_4/Source_data_Fig_4D.pptx]

## Slide 1
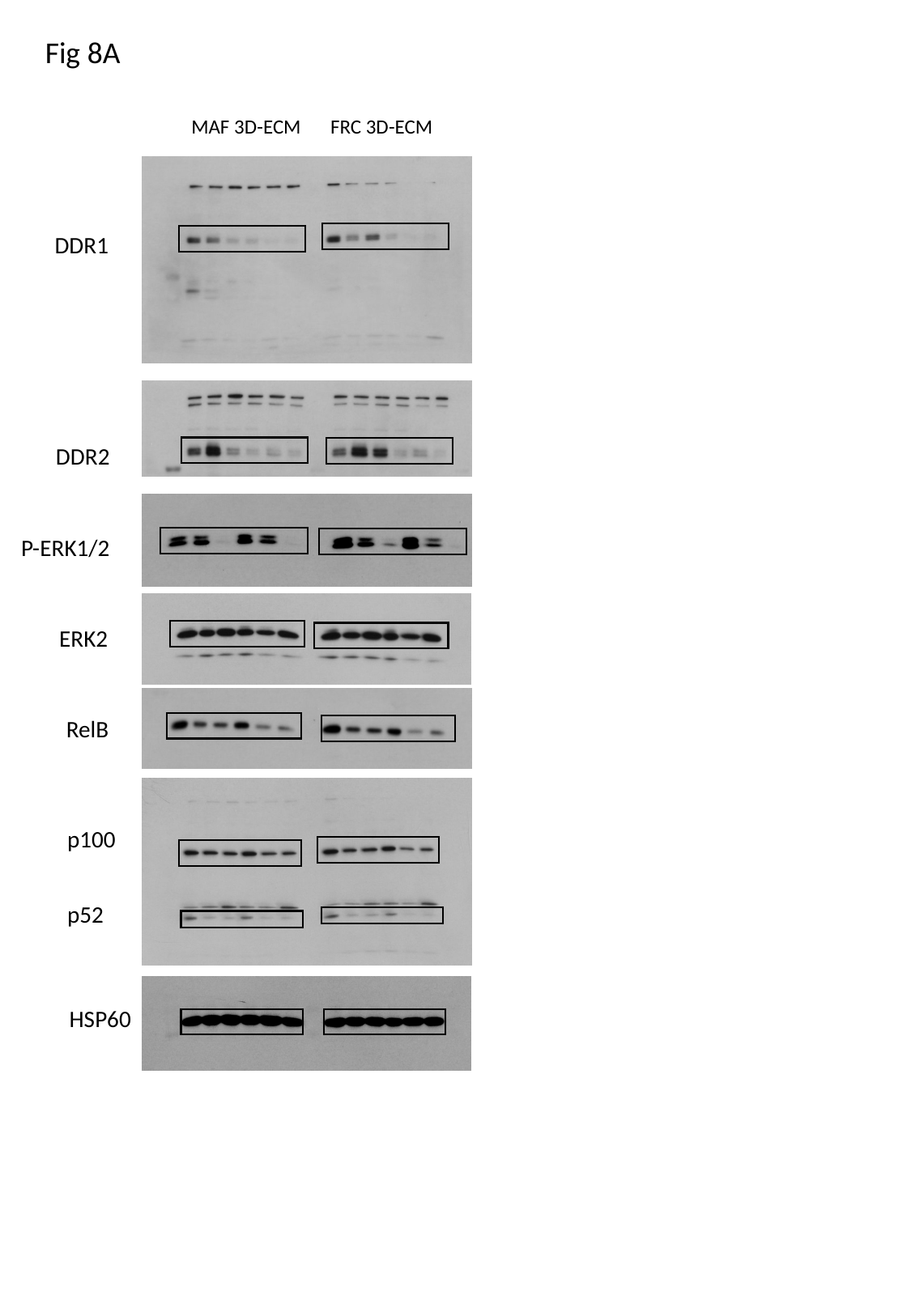

Fig 8A
MAF 3D-ECM
FRC 3D-ECM
DDR1
DDR2
P-ERK1/2
ERK2
RelB
p100
p52
HSP60

Supplement: Supplementary file 7 — Source Data for Figure 8 [file EMMM-14-e11814-s003.zip › Source_data_Figure_8/Source_data_Fig_8A.pptx]

## Slide 1
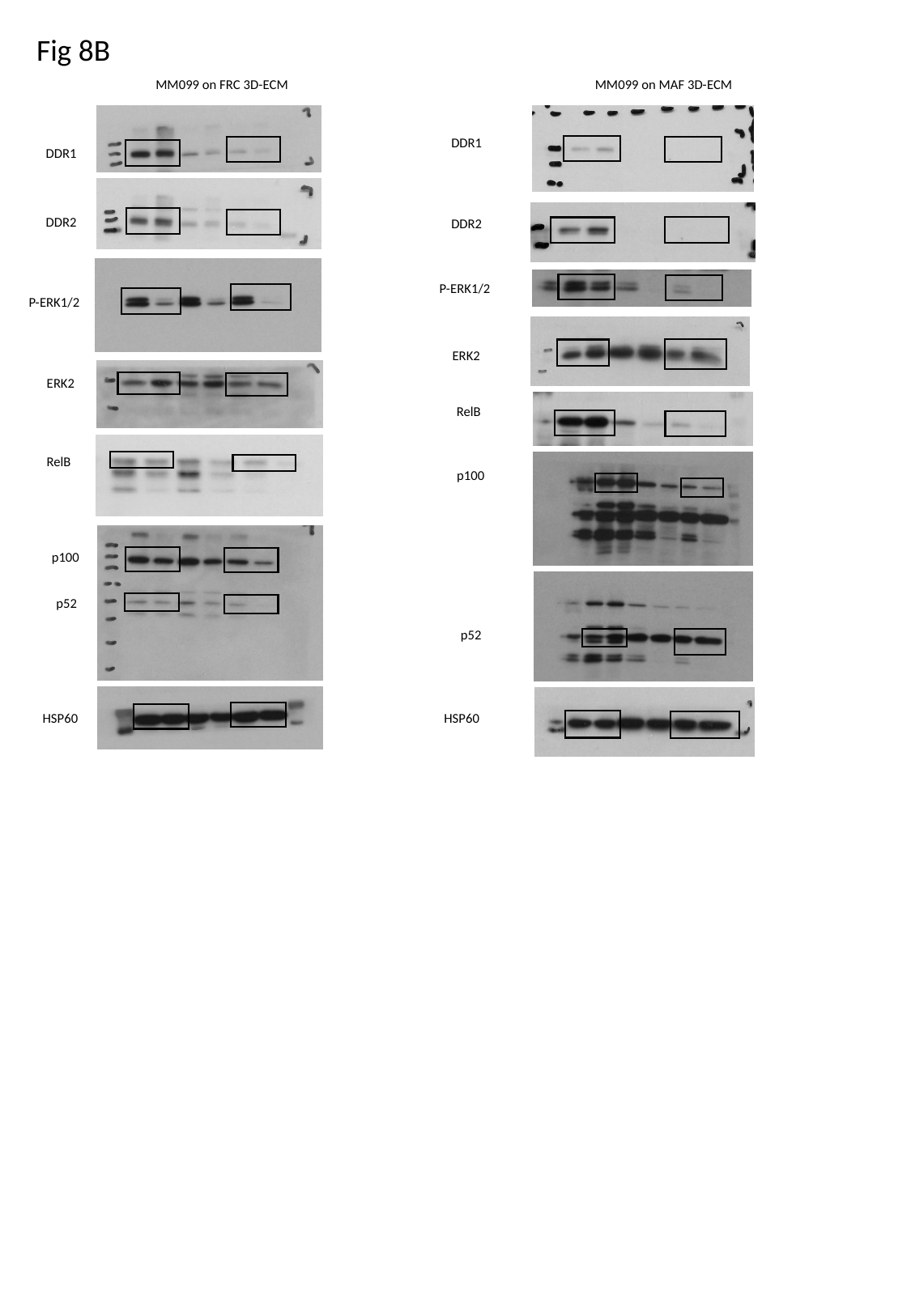

Fig 8B
MM099 on FRC 3D-ECM
MM099 on MAF 3D-ECM
DDR1
DDR1
DDR2
DDR2
P-ERK1/2
P-ERK1/2
ERK2
ERK2
RelB
RelB
p100
p100
p52
p52
HSP60
HSP60

Supplement: Supplementary file 7 — Source Data for Figure 8 [file EMMM-14-e11814-s003.zip › Source_data_Figure_8/Source_data_Fig_8B.pptx]

## Slide 1
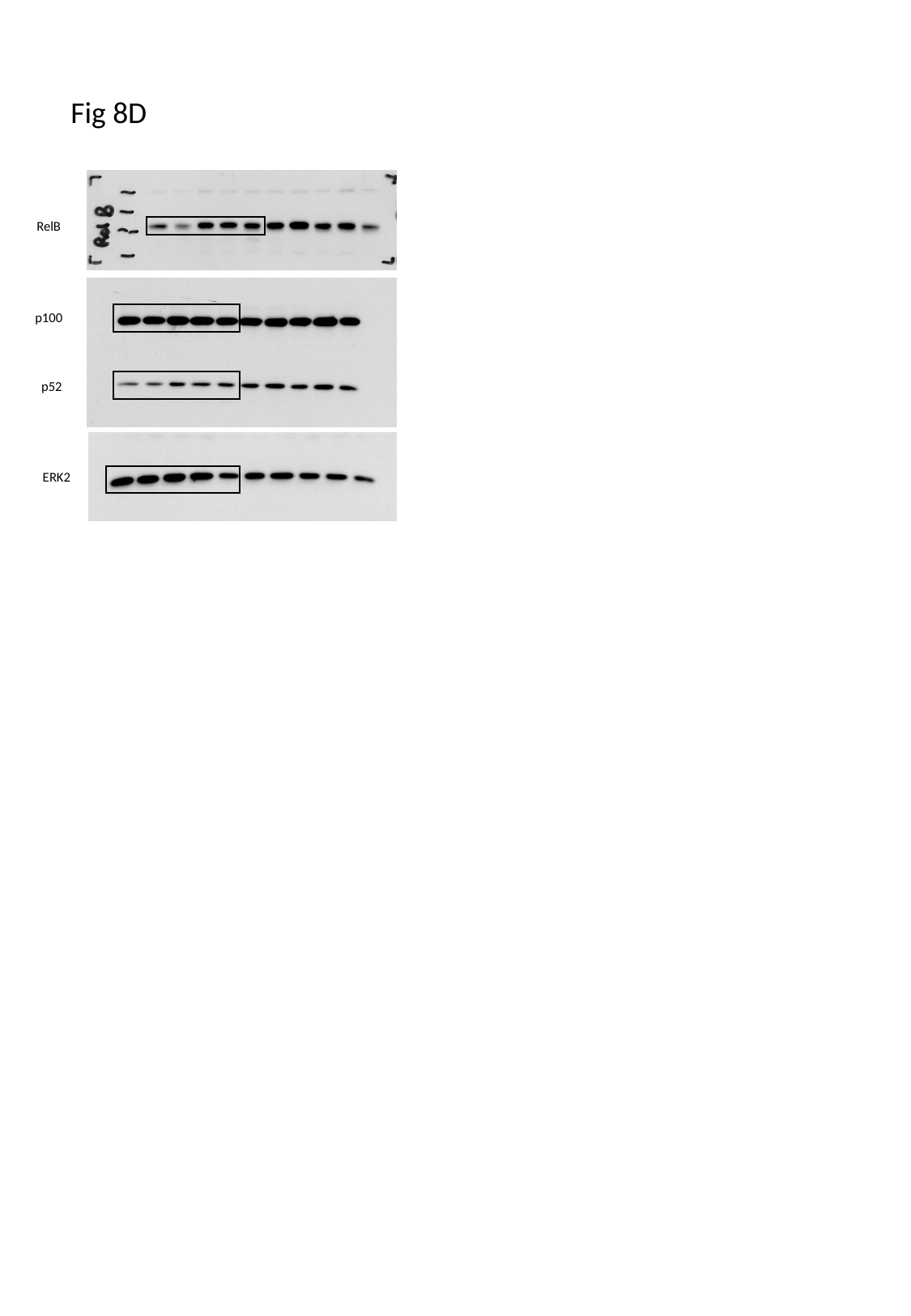

Fig 8D
RelB
p100
p52
ERK2

Supplement: Supplementary file 7 — Source Data for Figure 8 [file EMMM-14-e11814-s003.zip › Source_data_Figure_8/Source_data_Fig_8D.pptx]

## Slide 1
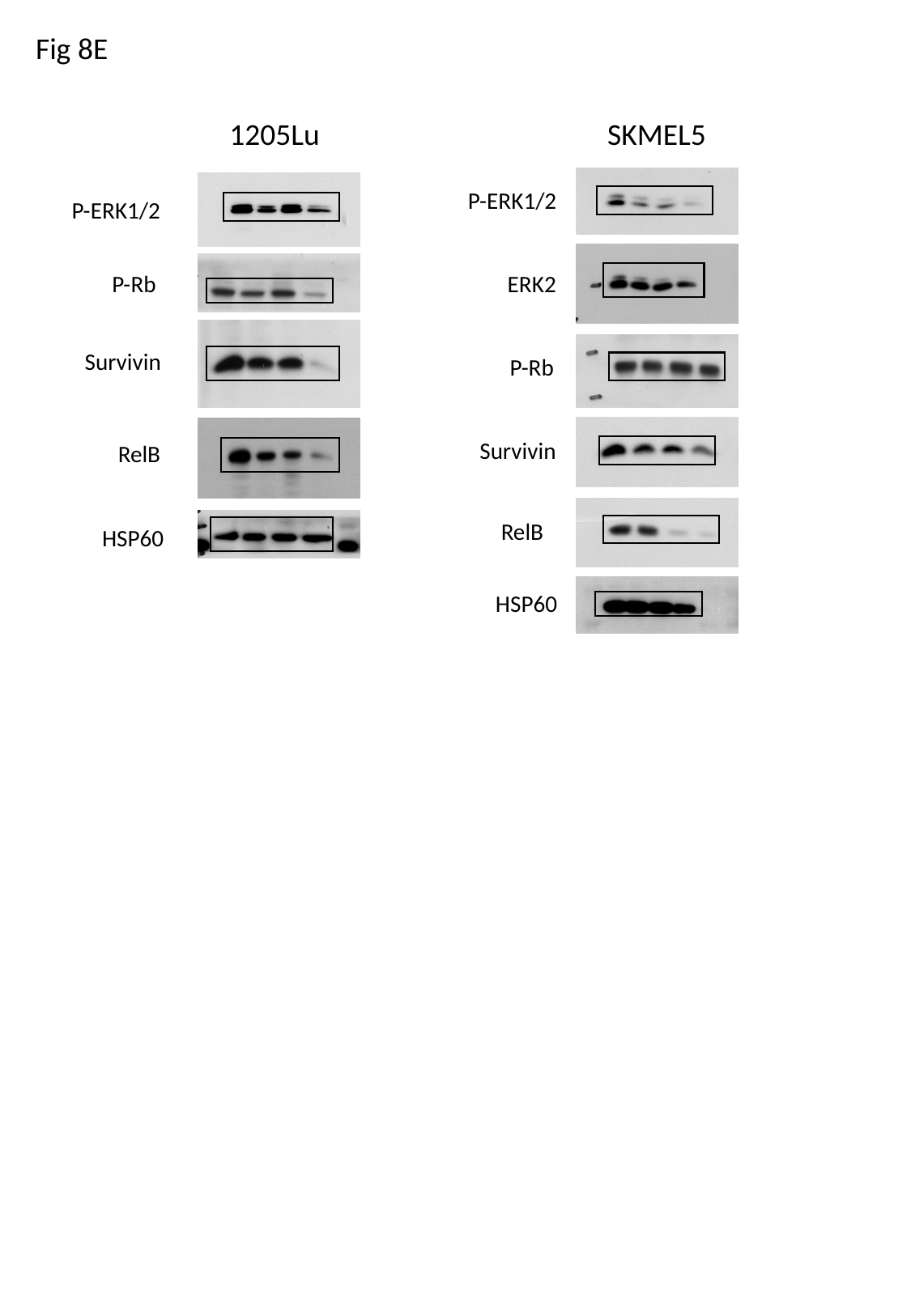

Fig 8E
1205Lu
SKMEL5
P-ERK1/2
P-ERK1/2
P-Rb
ERK2
Survivin
P-Rb
Survivin
RelB
RelB
HSP60
HSP60

Supplement: Supplementary file 7 — Source Data for Figure 8 [file EMMM-14-e11814-s003.zip › Source_data_Figure_8/Source_data_Fig_8E.pptx]

## Slide 1
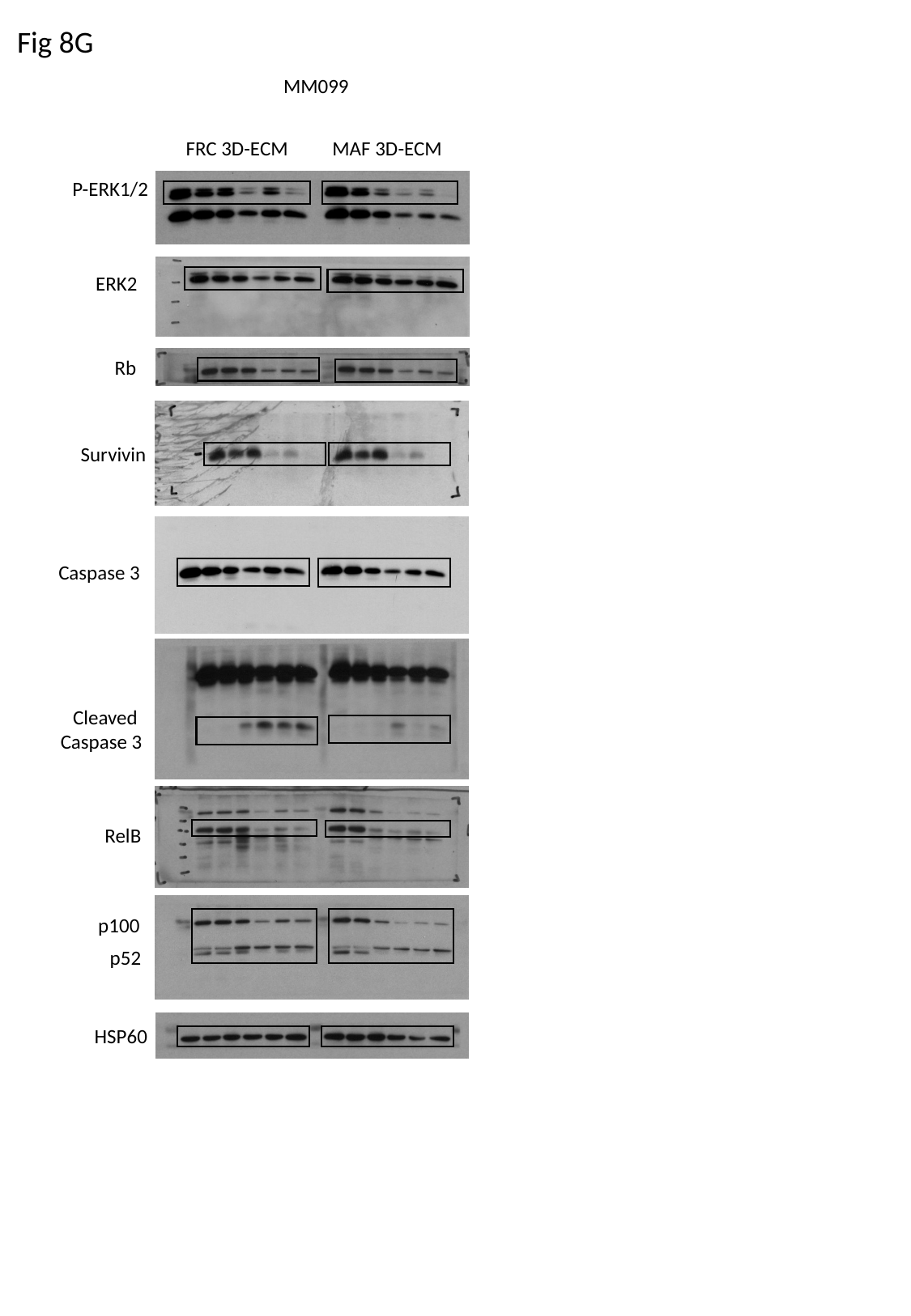

Fig 8G
MM099
MAF 3D-ECM
FRC 3D-ECM
P-ERK1/2
ERK2
Rb
Survivin
 Caspase 3
Cleaved
Caspase 3
RelB
p100
p52
HSP60

Supplement: Supplementary file 7 — Source Data for Figure 8 [file EMMM-14-e11814-s003.zip › Source_data_Figure_8/Source_data_Fig_8G.pptx]
