# Supplementary material for: Targeting Discoidin Domain Receptors DDR1 and DDR2 overcomes matrix‐mediated tumor cell adaptation and tolerance to BRAF‐targeted therapy in melanoma
Source: EMBO Mol Med. 2021 Dec 27;14(2):e11814. doi: 10.15252/emmm.201911814 (PMC8819497; doi:10.15252/emmm.201911814)
Supplement: Supplementary file 5 — Source Data for Figure 4 [file EMMM-14-e11814-s006.zip › Source_data_Figure_4/Source_data_Fig_4B.pptx]

## Slide 1
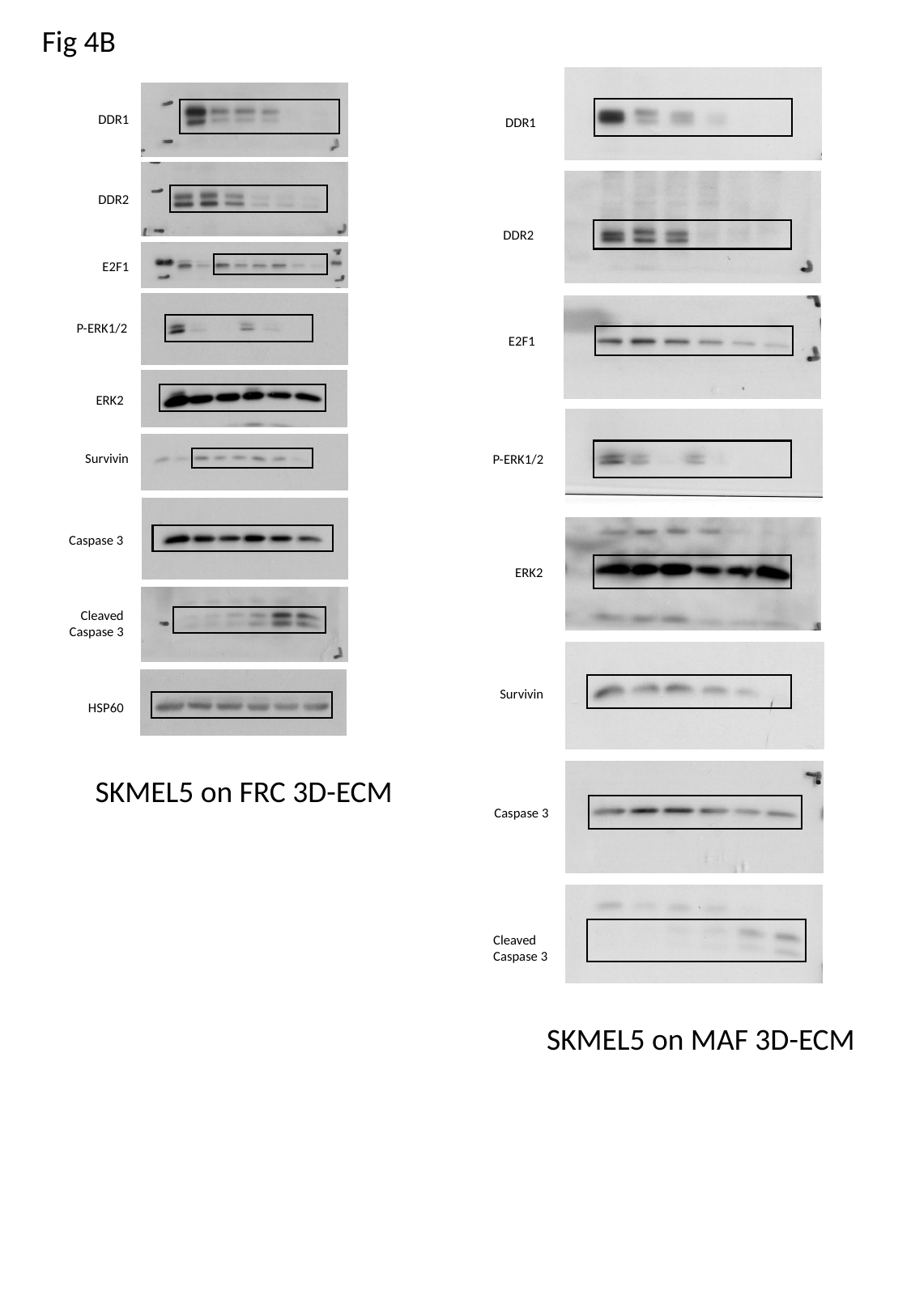

Fig 4B
DDR1
DDR2
E2F1
P-ERK1/2
ERK2
Survivin
Caspase 3
Cleaved
Caspase 3
DDR1
DDR2
E2F1
P-ERK1/2
ERK2
Survivin
Caspase 3
Cleaved
Caspase 3
HSP60
SKMEL5 on FRC 3D-ECM
SKMEL5 on MAF 3D-ECM
