# Supplementary material for: Targeting Discoidin Domain Receptors DDR1 and DDR2 overcomes matrix‐mediated tumor cell adaptation and tolerance to BRAF‐targeted therapy in melanoma
Source: EMBO Mol Med. 2021 Dec 27;14(2):e11814. doi: 10.15252/emmm.201911814 (PMC8819497; doi:10.15252/emmm.201911814)
Supplement: Supplementary file 7 — Source Data for Figure 8 [file EMMM-14-e11814-s003.zip › Source_data_Figure_8/Source_data_Fig_8F.pptx]

## Slide 1
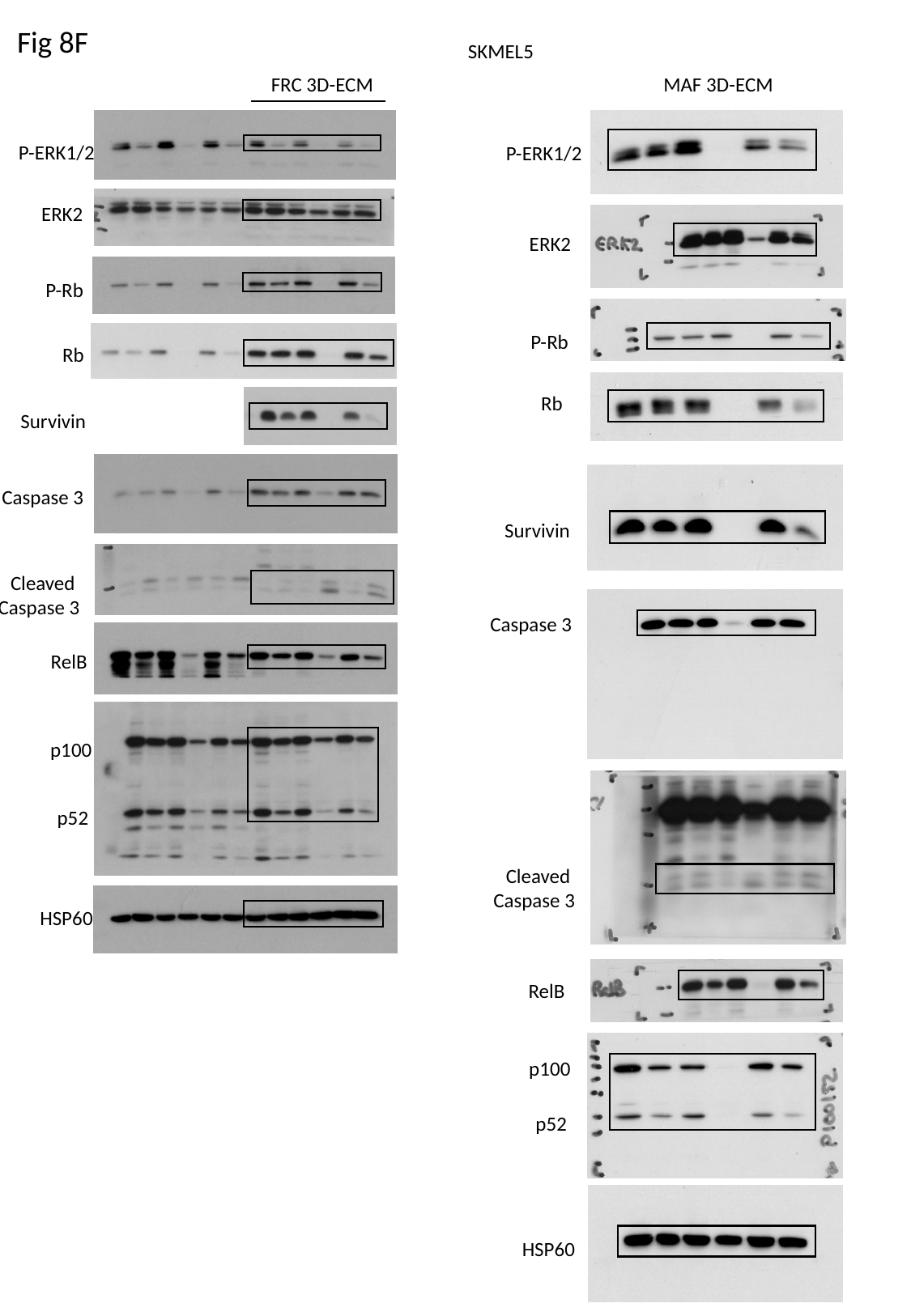

Fig 8F
SKMEL5
FRC 3D-ECM
MAF 3D-ECM
P-ERK1/2
P-ERK1/2
ERK2
ERK2
P-Rb
P-Rb
Rb
Rb
Survivin
 Caspase 3
Survivin
Cleaved
Caspase 3
 Caspase 3
RelB
p100
p52
Cleaved
Caspase 3
HSP60
 RelB
p100
p52
HSP60
